# Synthesis of *N*-Tosyl Allylic Amines from Substituted Alkenes via Vanadoxaziridine Catalysis

**DOI:** 10.1021/acs.joc.3c02859

**Published:** 2024-02-26

**Authors:** Rufai Madiu, Erin L. Doran, Jenna M. Doran, Ali A. Pinarci, Kiran Dhillon, Dominic A. Rivera, Amari M. Howard, James L. Stroud, Dylan A. Moskovitz, Steven J. Finneran, Alyssa N. Singer, Morgan E. Rossi, Gustavo Moura-Letts

**Affiliations:** Department of Chemistry and Biochemistry, Rowan University, 201 Mullica Hill Rd., Glassboro, New Jersey 08028, United States

## Abstract

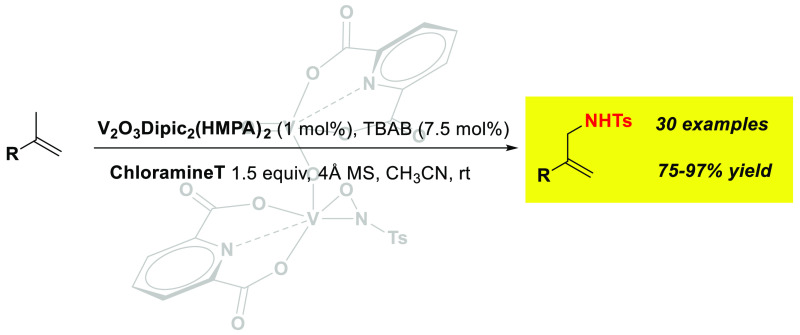

Herein, we report the catalytic allylic amination of α-methylalkenes with V_2_O_3_Dipic_2_(HMPA)_2_ and chloramine T as the quantitative source of *N*. The reaction works with high yields and stereoselectivities for α-methylalkenes. A proposed tosylnitrene-free catalytic cycle involving the formation of vanadoxaziridine complex **1** as the active catalyst and aminovanadation across the substrate as the rate-determining step has been proposed. Initial kinetic and competition experiments provide evidence for the proposed mechanism.

## Introduction

**Figure 1:**
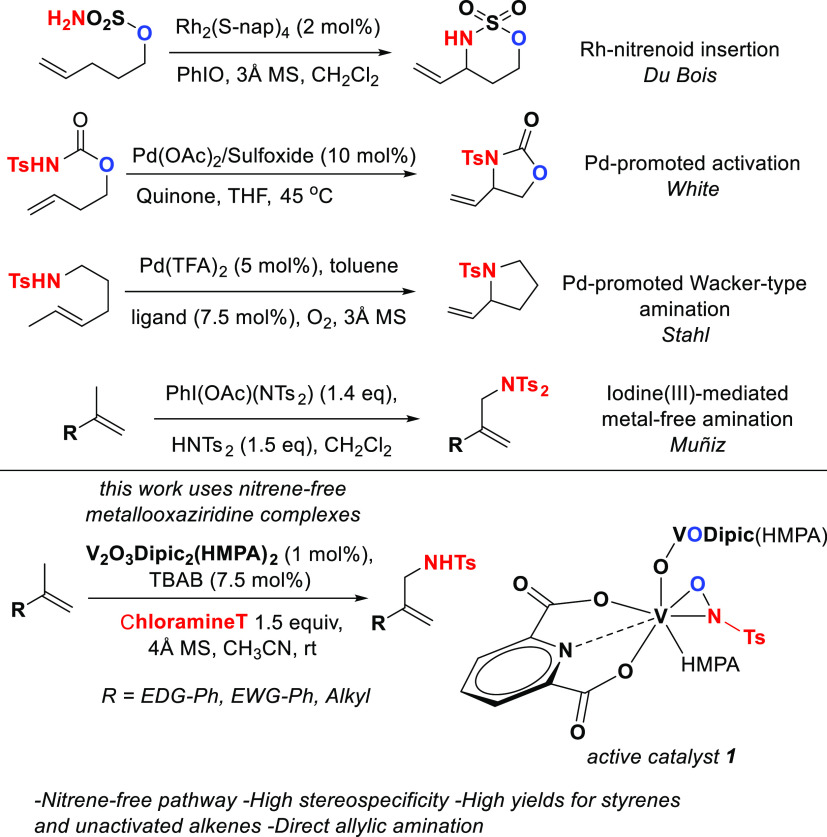
Advances in stereoselective allylic amination.

Nitrogen in the form of amines is found in agrochemicals, drug molecules, and synthetic materials.^1^ Nitrogen atoms also allow for molecules to have valuable pharmaceutical profiles due to enhanced physiological parameters.^2^ Thus, the continuous development of methods for amination reactions is of high significance. Among these, the direct allylic amination of alkenes signifies an ideal strategy by allowing atom-economical and single-step processes to access the desired nitrogen-containing molecules.^3^ Many efforts have been focused on the development of transition metal-mediated allylic amination reactions.^4^ Interestingly, most of these rely on the formation of metal nitrenoids in the form of metal imido or nitrene intermediates to achieve the activation process.^5^ On the other hand, transition metal activation followed by nitrogen addition and Wacker-type oxidative processes have also received significant attention.^6^ Moreover, metal-free methods involving formal C–H functionalization have also been studied (Figure 1).^7^ These reactions have become ubiquitous in organic chemistry to the extent of being extensively used in late-stage functionalization of complex molecules and in many total syntheses.^8^

However, most suffer from poor regioselectivities or rely on intramolecular methods that often hinder access to the targeted functionality. Thus, direct access through allylic amination processes is of great value in the field of organic chemistry.

Metallooxaziridines were discovered by reacting metal oxides and *N*-containing molecules for the functionalization of alkenes.^9^ Their properties are comparable to metal peroxo complexes used in *O*-transfer reactions.^10^ The chemistry of these metallooxaziridines has not been fully developed; however, we recently reported that zirconoxaziridines are suitable catalysts for the highly stereoselective and stereospecific aziridination of unactivated alkenes.^11^ Moreover, the combination of high-oxidation state metal oxides, tridentate ligands, and phenyl hydroxylamines is known to provide metallooxaziridines and in some examples reactive intermediates for *N*-transfer reactions.^12^ Some early work by Sharpless led to some of the unique properties of these complexes as he found that *Mo*-oxaziridines were able to activate π-systems.^13^ The Moura-Letts laboratory is focused on developing novel methods for the synthesis of complex nitrogen-containing molecules.^14^

Thus, we envisioned creating a well-defined catalytic system for the allylic amination of alkenes *via* the formation of metallooxaziridines as the active catalyst for *N*-transfer reactions. To the best of our knowledge, this would be the first report for such catalytic process.

## Results and Discussion

Based on our current efforts toward metallooxaziridine-mediated aziridination and oxyamination reactions, we discovered that other potential transformations could become synthetically useful upon changing the transition metal in the metallooxaziridine center. The high chemoselectivity observed for LZrON-Ts in the aziridination of alkenes was in part due to the group IV transition metal Lewis acidity and the geometry of the respective lowest unoccupied molecular orbital (LUMO) across the *Zr–N* bond. On the other hand, group VI complexes (LWO_2_NTs) with increased π-acidity lead to oxyamination products with high chemoselectivity. Thus, it was hypothesized that group V complexes (L_2_V_2_O_3_NTs) with decreased π-acidity would provide LUMOs, leading to predictable aminovanadation pathways.^15^ Fortunately, it was discovered that while L_2_V_2_O_3_NTs provided aziridine in very low yield, it also provided *N*-tosyl allylic amine **3a** with high selectivity.

The basic premise behind this study was to develop a simple and efficient allylic amination process with a high selectivity for alkenes (Table 1). Initial results using α-methylstyrene indicated that metal oxides at 5 mol % with a phase-transfer catalyst (CTAB, 7.5 mol %) and excess chloramine T in CH_3_CN promoted allylic amination in low yields, with a clear indication that *V* was able to provide the expected product with synthetically useful selectivities (entry 2). When Dipic was used as the ligand, it was found that V_2_O_3_Dipic_2_(HMPA)_2_ improved the reaction yield to 64% with 12:1:0 **3a**:**4a**:**5a** product ratio (entry 6); *W* or *Mo* was not as successful at improving the reaction yield (entries 5 and 7). The nature of the PTC was tested, and it was found that TBAB provided a significant increase in reaction performance (91% yield, 16:1:0 ratio, entry 8). Due to the different catalytic steps taking place in different phases of the reaction, effective PTCs improve the transfer of insoluble chloramine T across the reaction phases. Other catalysts failed to improve the reaction yield beyond the efficiency obtained with TBAB (entries 9 and 10). Interestingly, PTC loading at 15% had a diminishing effect on the reaction conversion (79%, entry 11).

**Table 1:**
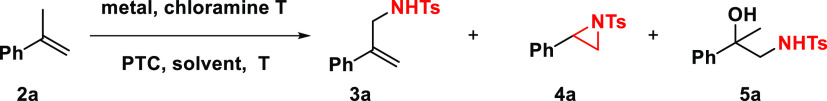
Reaction Optimization

entry	metal	PTC	solvent	temperature	yield (%)^a^^,^^b^	**3a**:**4a**:**5a**
1	MoO_3_^c^	CTAB	CH_3_CN	rt	12	1:1:1
2	V_2_O_5_^c^	CTAB	CH_3_CN	rt	24	4:1:0
3	WO_3_^c^	CTAB	CH_3_CN	rt	8	0.5:2:1
4	ZrO_2_^c^	CTAB	CH_3_CN	rt	12	0:1:0
5	WO_2_Dipic^c^^,^^d^	CTAB	CH_3_CN	rt	10	0:0.5:8
6	V_2_O_3_Dipic_2_^c^^,^^e^	CTAB	CH_3_CN	rt	64	12:1:0
7	MoO_2_Dipic^c^^,^^f^	CTAB	CH_3_CN	rt	35	2:1:0
8	V_2_O_3_Dipic_2_^c^^,^^e^	TBAB	CH_3_CN	rt	91	16:1:0
9	V_2_O_3_Dipic_2_^c^^,^^e^	TBAI	CH_3_CN	rt	82	16:1:0
10	V_2_O_3_Dipic_2_^c^^,^^e^	TBACl	CH_3_CN	rt	62	16:1:0
11	V_2_O_3_Dipic_2_^c^^,^^e^	TBAB^g^	CH_3_CN	rt	79	16:1:0
12	V_2_O_3_Dipic_2_^e^ (10 mol %)	TBAB^g^	CH_3_CN	rt	75	16:1:0
13	V_2_O_3_Dipic_2_^e^ (1 mol %)	TBAB	CH_3_CN	rt	95	20:1:0
14	V_2_O_3_Dipic_2_^e^ (1 mol %)	TBAB	DCE	rt	88	16:1:0
15	V_2_O_3_Dipic_2_^e^ (1 mol %)	TBAB	CH_2_Cl_2_	rt	73	16:1:0
16	V_2_O_3_Dipic_2_^e^ (1 mol %)	TBAB	DMF	rt	35	16:1:0
17	V_2_O_3_Dipic_2_^e^ (1 mol %)	TBAB	CH_3_CN	0 °C	80	20:1:0
18	V_2_O_3_Dipic_2_^e^ (1 mol %)	TBAB	CH_3_CN	60 °C	24	8:1:2

a Isolated yields.

b To a solution of chloramine T (1.5 equiv) in CH_3_CN, V_2_O_3_Dipic_2_(HMPA)_2_, 4Å MS (200 mg/mmol), TBAB, and alkene are added and stirred at rt for 12 h.

c 5 mol % of metal and 7.5 mol % of PTC.

d WO_2_Dipic(H_2_O), Dipic = dipicolinic acid.

e V_2_O_3_Dipic_2_(HMPA)_2_.

f MoO_2_Dipic(HMPA).

g 15 mol %.

To further improve the reaction performance, catalyst loading was increased to 10 mol %, but a complex mixture and lower chemoselectivity due to catalyst decomposition was observed (entry 12); however, when loading was reduced to 1 mol %, the yield and product ratio increased to 95% and 20:1:0, respectively (entry 13). The reaction solvent was also examined, and CH_3_CN remained optimal (entries 14–16). Coordinating solvents (CH_3_CN) are known to accelerate *N*-Ts bond transfer reactions. The reaction at different temperatures also failed to provide improved yields and selectivities (entries 17 and 18).

Given the results observed for the allylic amination of α-methylstyrene, this study focused on addressing the generality across α-methylstyrenes with electron-donating groups (EDGs) and electron-withdrawing groups (EWGs) as a means to activate or deactivate the allylic amination pathway (Table 2). 4-Methyl-α-methylstyrene worked in a similar yield at a significantly faster observable rate (93%, 6 h, entry 2). Moreover, 4-ethyl, 4-isopropyl, 4-tbutyl, 4-isobutyl, and 4-phenyl all provided the corresponding allylic amine **3** in great yields and 3–4 h reaction times (entries 3–7). 4-MeO-α-methylstyrene was also very successful at furnishing **3**, but reaction completion was observed at 1.5 h (entry 8). Interestingly, 3-MeO and 2-MeO were obtained in equally high yields, but full reaction completion was only achieved after 2 and 4 h, respectively (entries 9 and 10). Efforts to expand the scope with other EDGs found that 4-ethoxy, 4-thiomethyl, and 2,4-dimethoxy achieved full conversion at fast rates, but isolated yields were lower due to apparent decomposition during purification (2–3 h, entries 11–13). We anticipated that halogenated α-methylstyrenes would react with equal efficiencies but at slower rates, and we found that 4-bromo, 3-bromo, and 2-bromo reacted with high yields but slower observable rates (10, 12, and 72 h, respectively, entries 14–16). 4-Fluoro, 4-chloro, 4-iodo, 4-trifluoromethyl, and 4-*p*-bromophenyl all proceeded with high yields and similar reaction rates (9–11 h, entries 17–21). The scope was completed with the study of α-methylstyrenes with EWGs: 4-nitro, 3-nitro, 2-nitro, and 4-cyano provided the corresponding allylic amine **3** in synthetically useful yields but at much slower observable rates (20, 24, 48, and 18 h, respectively, entries 22–25). The reaction across α-methylstyrenes proved to be very successful, and kinetic data indicate a clear reaction acceleration with EDGs and reaction deceleration with EWGs.

**Table 2:**
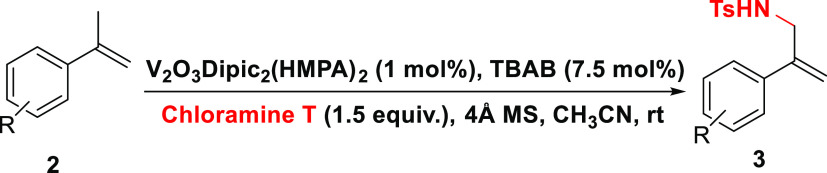

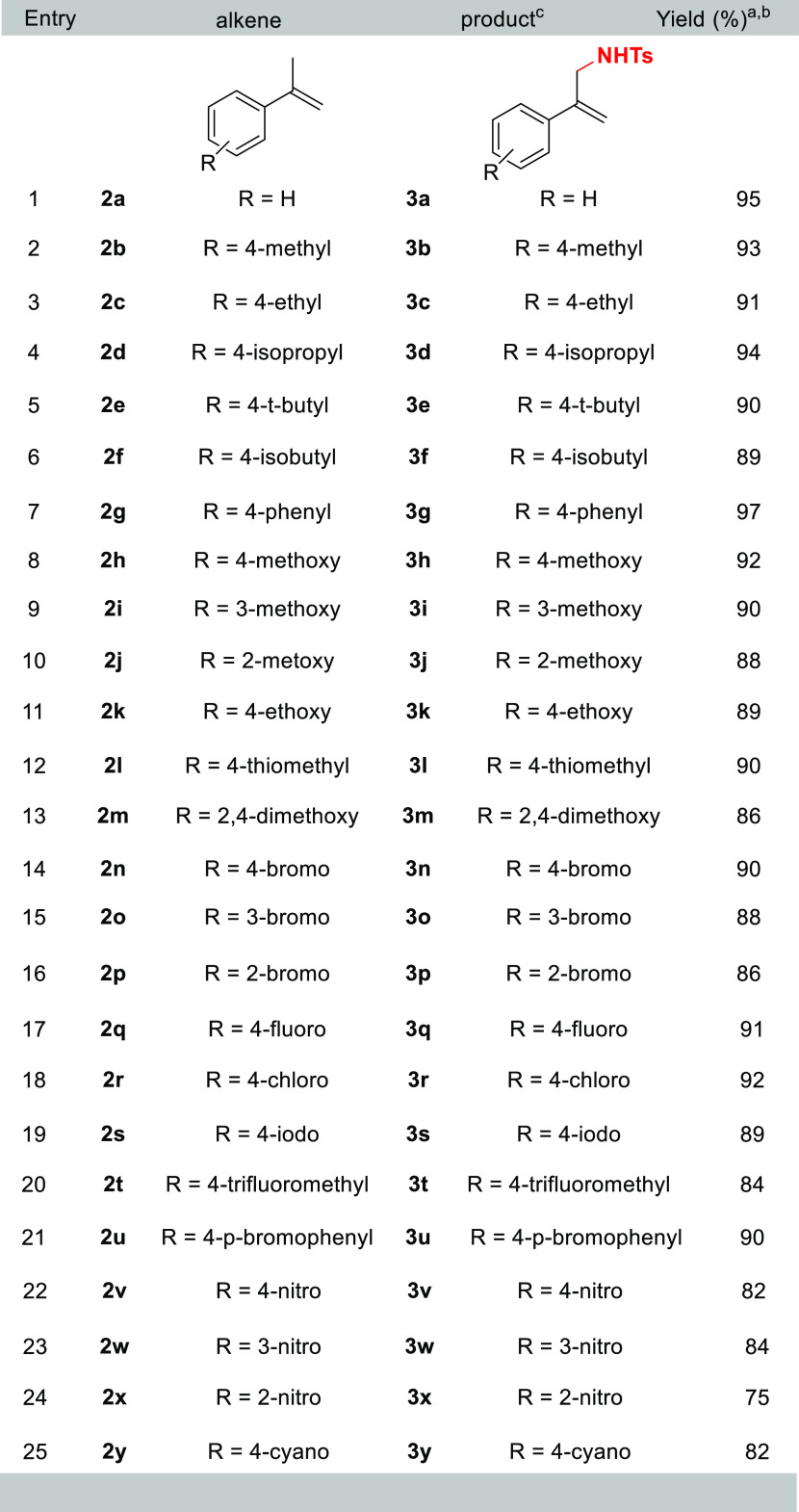
Reaction Scope

a Conditions: To a solution of chloramine T in CH_3_CN, 4Å MS, TBAB, and alkene are added and reaction stirred at rt for 0.5–20 h.

b Isolated yields.

c The reaction was purified by standard silica gel chromatography.

The focus then turned to addressing the scope across unactivated alkenes. The potential for multiple product formation was anticipated due to the availability of multiple elimination sites for these substrates (Table 3). However, we were surprised to find that 2,3-dimethylbutene reacted to provide allylic amine **3z** in 91% yield as a single alkene isomer. The reaction was significantly slower than the α-methylstyrene counterparts (18 h, entry 1). 2-Methylpentene, 2-methylhexene, and 2-methylheptene confirmed the reaction trend by furnishing allylic amine **3** as single isomers (18–22 h, entries 2–4). Finally, 2-methyl-3-*(p-*MeO-phenyl)-propene also provided **3** in high yield and as a single isomer (15 h, entry 5). Activation of alkenes, depending upon the nature of the *N*-transfer reagent, often suffers from poor selectivities due to the formation of nitrene reactive intermediates that lead to stereochemical erosion.^16^ Analogous to the aziridination reaction, high specificity was expected due to the formation of a potential metalloheterocyclic intermediate rather than nitrene species. Alkene oxidations with similar vanadium (V) oxide catalysts are widely known, and Mimoun has reported vanadoheterocyclic intermediates for their reaction mechanisms.^ ^15a,17^^ The clear preference for a CH_3_-selective elimination pathway was further verified when cyclohexene and methylenecyclohexane failed to achieve any conversion at room temperature. These results together with initial kinetic data indicate that the key elimination step is very fast and therefore not the rate-determining step.

**Table 3:**
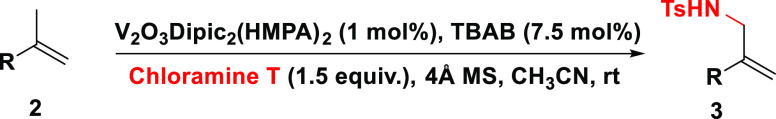

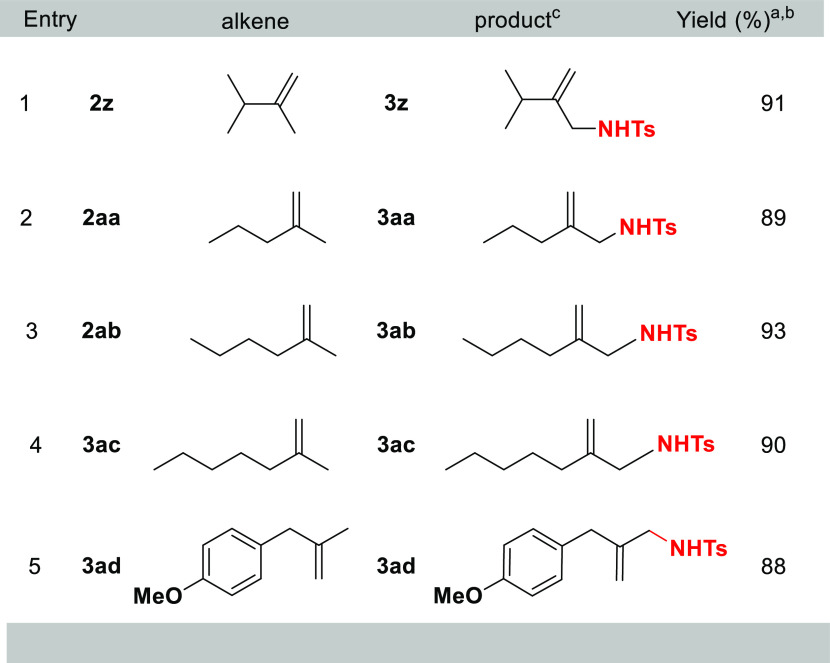
Reaction Scope

a Conditions: To a solution of chloramine T in CH_3_CN, 4Å MS, TBAB, and alkene are added and reaction stirred at rt for 24 h.

b Isolated yields.

c The reaction was purified by standard silica gel chromatography.

The mechanism studies for this transformation are based on the foundational knowledge discovered for the zirconoxaziridine-mediated aziridination of alkenes. Thus, kinetic and model reaction studies were designed based on that effort.

Due to the prevalence of nitrene-mediated allylic amination pathways, we first wanted to determine if the reaction proceeded via radical abstraction. Control experiments using radical scavengers (TEMPO and BHT) failed to inhibit the reaction. Moreover, experiments with deuterated substrates showed that **2a2-*****d***_***3***_ reacted to provide **3a2-*****d***_***2***_ without any deuterium scrambling, thus both experiments confirming that a radical-mediated process was unlikely to be the predominant pathway and that a metalloheterocyclic intermediate was the predominant pathway. We also wanted to address if **3** formed through a pseudoallylic amination process by the elimination of aziridine **4** under the reaction conditions, and we found that **4a** does not form **3a** under the reaction conditions. Other control experiments showed that V_2_O_3_Dipic_2_(HMPA)_2_ is crucial for reaction conversion; thus, no halogen-mediated activation pathway is taking place and that the reaction also proceeds under stoichiometric amounts of **1** (low conversion, details in the Supporting Information (SI)).

**Figure 2:**
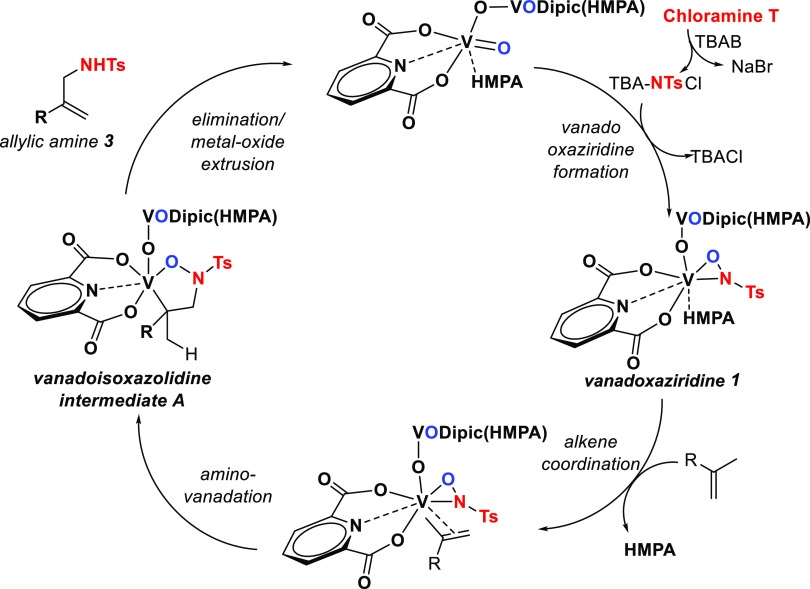
Proposed catalytic cycle.

Thus, we propose a catalytic cycle (Figure 2) in which **l** forms within minutes and essentially quantitatively at rt. **1** can also be efficiently isolated by reacting V_2_O_3_Dipic_2_(HMPA)_2_ and chloramine T in MeOH, and its presence can be detected in the catalytic reaction mixture spectroscopically.^18^ Followed by ligand exchange at the vanadoxaziridine metal center, this step allows for alkene coordination and the proper alignment of the reactive MOs on the metal complex to efficiently react across the *V–N* bond. Intermediate **A** then forms via *syn*-aminovanadation across the alkene system, as the rate-determining step. Intermediate **A** then undergoes fast CH_3_-selective elimination to provide allylic amine **3** and V_2_O_3_Dipic_2_(HMPA)_2_.^19^

**Figure 3:**
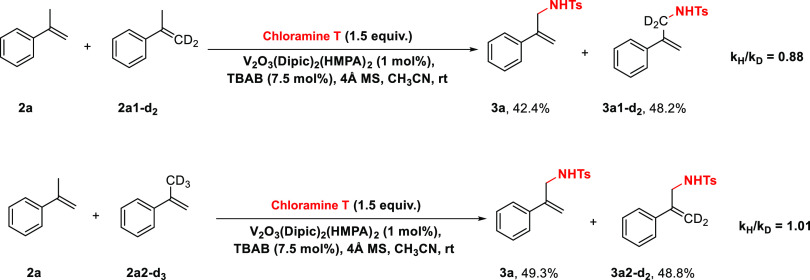
Deuterium labeling competition studies.

Further analysis of the reaction mechanism determined that the elimination step is exclusively selective for CH_3_ and alkenes with different substitution patterns do not provide allylic amination products (details in the SI), thus validating a fast elimination step. Experimental verification was obtained by further deuterium labeling competition studies (Figure 3). The competition studies showed an inverse secondary kinetic isotope effect (KIE) when reacting a 1:1 mixture of **2a/2a1-*****d***_***2***_ (*k*_H_/*k*_D_ = 0.88) and no primary kinetic isotope effect (*k*_H_/*k*_D_ = 1.01) when reacting a 1:1 mixture of **2a/2a2-*****d***_***3***_. The presence of an inverse secondary KIE correlates well with a slow, rate-determining step, aminovanadation, while no primary KIE correlates with fast CH_3_ elimination. The proposed mechanism is in further agreement with a Hammett correlation study employing α-methylstyrenes (2a, b, e, h, q–s). These results show a ρ-value of −2.15 and hence demonstrate an enhanced reactivity for α-methylstyrenes with EDGs as a positive charge develops in the transition state (details in the SI). Thus, these results are also in agreement with the proposed aminovanadation step as the rate-determining step for this catalytic cycle.

## Conclusions

In summary, these efforts have discovered a novel vanadoxaziridine-mediated catalytic allylic amination of alkenes. The transformation works with high efficiency and selectivity for alkenes with diverse substitution patterns and for styrenes with a collection of functional groups. The reaction mechanism involves formation of *N*-Ts vanadoxaziridine active catalyst **1**, followed by delivery of *N*-Ts through the formation of intermediate **A** and by elimination and vanadium oxide extrusion to provide allylic amine **3**. Further characterization experiments and computational studies to better understand the catalytic cycle are ongoing, and a follow up article is in preparation.

## Data Availability

The data underlying this study are available in the published article and its Supporting Information.

## Supporting Information Available

Experimental protocols and spectroscopic data for each allylic amine (PDF)
