# Supplementary material for: Synthesis of N-Tosyl Allylic Amines from Substituted Alkenes via Vanadoxaziridine Catalysis
Source: J Org Chem. 2024 Feb 26;89(6):4001–8. doi: 10.1021/acs.joc.3c02859 (PMC10949238; doi:10.1021/acs.joc.3c02859)
Supplement: Supplementary file 1 — jo3c02859_si_001.pdf [file jo3c02859_si_001.pdf]

Synthesis of N-Tosyl Allylic Amines from Substituted Alkenes via Vanadoxaziridine Catalysis

Rufai Madiu, Erin L. Doran, Jenna M. Doran, Ali A. Pinarci, Kiran Dhillon, Dominic A. Rivera, Amari M. Howard, James L. Stroud, Dylan A. Moskovitz, Steven J. Finneran, Alyssa N. Singer, Morgan E. Rossi and Gustavo Moura-Letts*

moura-letts@rowan.edu
Department of Chemistry and Biochemistry, College of Science and Mathematics
Rowan University
201 Mullica Hill Rd, Glassboro, NJ, 08028

Supporting Information

A. Materials and Methods S1
B. Synthesis of Vanadoxaziridine **1** S9
C. Synthesis of allylic amines from **Table 2-3** S10
D. ^{1}H-NMR and ^{13}C-MR spectra S22

A. MATERIALS AND METHODS

Reagents were obtained from Aldrich Chemical (www.sigma-aldrich.com), Acros Organics (www.us.vwr.com) or Alfa Aesar (www.us.vwr.com) and used without further purification. Solvents were obtained from EMD Miliphore DrySol (www.us.vwr.com) and degassed with N_2. Solution phase reactions were performed in glass vials or round bottom flasks with inert atmosphere and magnetic stirring. Cold baths were generated as follows: 0 °C, wet ice/water; –10 °C, ice/acetone; –20 °C, dry ice/isopropanol monitored with a thermometer; –44 °C, dry ice/CH_3CN; –63 °C, dry ice/chloroform; –78 °C, dry ice/acetone; –100 °C, liquid nitrogen - hexanes/Et_2O. Heated reactions were performed using IKA heating blocks. TLC was performed on 0.25 mm E. Merck silica gel 60 F254 plates and visualized under UV light and/or the following stain solutions: cerium ammonium molybdate (CAM), phosphomolybdic acid (PMA), iodine (I_2), or *p*-anisaldehyde. Silica flash chromatography was performed on E. Merck 230–400 mesh silica gel 60. Automated chromatography was performed on an ISOLERA Prime instrument with 10 g. SNAP silica gel normal phase cartridges using a flow rate of 12.0 mL/min and a gradient of 0–100% EtOAc in heptanes over 20 min with UV detection at 254 nm. NMR spectra were recorded on a Bruker Avance Neo 400 MHz Spectrometer at 24 °C in $CDCl_3$ unless otherwise indicated. Chemical shifts are expressed in ppm relative to TMS (^1H, 0 ppm) or solvent signals: $CDCl_3$ (^1H, 7.23 ppm; ^{13}C, 77.0 ppm); coupling constants are expressed in Hz. Low- and high-resolution mass spectroscopy was performed on an Agilent 6230 Accurate-Mass Time-of-Flight 1290 Infinity UHPLC/MS.

All α-methyl-alkene substrates were commercially available and used as received. Substrates **2c**, **2d**, **2e**, **2f**, **2g**, **2h**, **2i**, **2j**, **2k**, **2m**, **2n**, **2o**, **2q**, **2r**, **2s**, **2t**, **2u**, **2y**, and **2ad** were synthesized according to literature procedures.[1] The ^1H-NMR spectra for these are included in this document and match the ones previously reported.

[1] S. H. Pine, G. S. Shen, H. Hoang, Ketone Methylenation using the Tebbe and Wittig Reagents-a Comparison *Synthesis* **1991**, 165.

General method for the synthesis of α-methyl-alkene 2: Potassium tert-butoxide (8.3 mmol, 1.3 equiv.) was added to the suspension of methyltriphenylphosphonium bromide (8.3 mmol, 1.3 equiv.) in dry THF (0.2 M, 30 mL) at -78°C condition. After 1 h stirring at -78°C condition, a solution substituted acetophenone (6.4 mmol, 1 equiv.) in THF (1.3 M, 5 ml) was added dropwise. Then the reaction mixture was stirred at room temperature for 1 h. The crude was then filtered by a 1:1 silica gel/celite pad and the resulting mixture was then purified by silica gel chromatography to provide the corresponding α-Methyl-alkene **2**.

General method for the synthesis of allylic amine 3: In a 16 mL oven-dried and flushed with nitrogen vial packed with a magnetic stirrer, dry chloramine T (1.5 mmol, 1.5 equiv.) was mixed in CH_3CN (0.125M, 8 mL) and then 4Å MS (200 mg, 200mg/mmol), TBAB (0.075 mmol, 7.5 mol%), $V_2O_3Dipic_2(HMPA)_2$ (0.01 mmol, 1 mol%), and alkene **2** (1 mmol, 1 equiv.) were added. The reaction was then quickly flashed with nitrogen and allowed to stir at rt for 20 hours or until disappearance of alkene by TLC. The crude was then filtered by a 1:1 silica gel/celite pad and the resulting mixture was then purified by silica gel chromatography to provide the corresponding allylic amine **3**. **It is fundamental that chloramine T is dried on the same day at 80 °C under vacuum for 4h.*

Mechanism experiments:

Radical trapping experiment: This experiment aimed at addressing the potential formation of any transient radical species by a control experiment using TEMPO and BHT as radical traps. Using the same reaction as described in the general method (styrene as substrate), two reactions were set up: one with the addition of 1 equiv. of TEMPO and one with the addition of 1 equiv. of BHT. We observed no deviation on the conversion to the respective allylic amine. These results clearly indicate that no radical species actively participate in the allylic amination of alkenes using vanadoxaziridines.

Kinetic experiments:

Kinetic rates for alkene: In a 16 mL oven-dried and flushed with nitrogen vial packed with a magnetic stirrer, dry chloramine T (0.15 mmol, 1.5 equiv.) was mixed in CH_3CN (8 mL) and then 4Å MS (20 mg, 200mg/mmol), TBAB (0.0075 mmol, 7.5 mol%), $V_2O_3Dipic_2(HMPA)_2$ (0.001 mmol, 1 mol%). A standard solution of alkene **2b** in CH_3CN was prepared so it could be added from 0.01 to 0.1 mmol and the difference in volume corrected with CH_3CN to achieve constant volume. After addition of alkene, aliquots (200 µL) were removed, filtered by a 1:1 silica gel/celite pad, washed with EtOAc and the resulting mixture was then analyzed by ^{1}H-NMR to quantify the formation of allylic amine **3b**.

Alkene M	Rate (mmol/min)
0.00125	0.0000280
0.0025	0.0000580
0.005	0.0001564
0.0075	0.0002690
0.01	0.0003829
0.0125	0.0004888

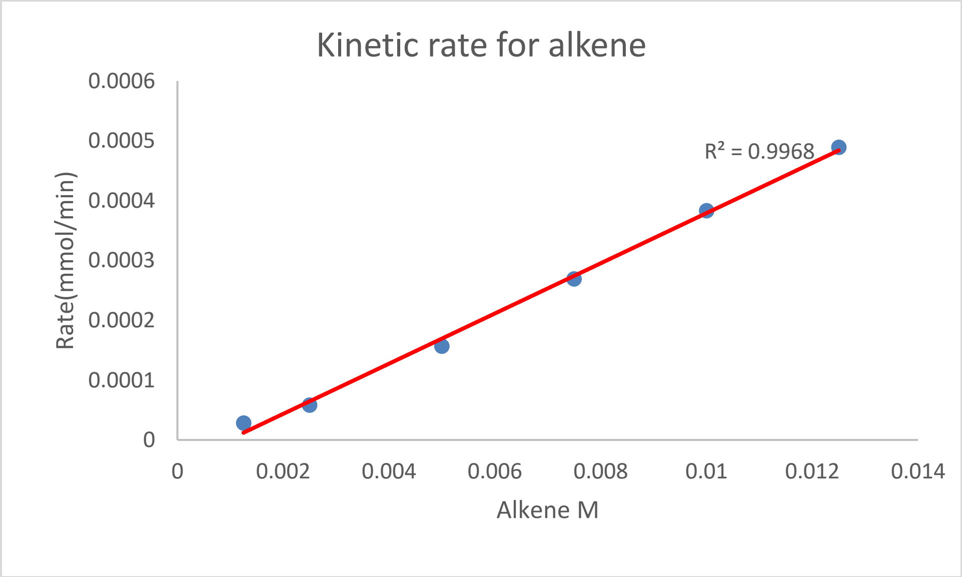

Figure S1. Kinetic rate for alkene plot.

Kinetic rates for chloramine T: In a 16 mL oven-dried and flushed with nitrogen vial packed with a magnetic stirrer, 4Å MS (20 mg, 200mg/mmol), TBAB (0.0075 mmol, 7.5 mol%), $V_2O_3Dipic_2$ $(HMPA)_2$ (0.001 mmol, 1 mol%), alkene **2b** (0.1 mmol, 1equiv.) were mixed in CH_3CN (8 mL). A standard solution of dry chloramine T in CH_3CN was prepared so it could be added from 0.01 to 0.125 mmol and the difference in volume corrected with CH_3CN to achieve constant volume. After addition of alkene, aliquots (200 µL) were removed, filtered by a 1:1 silica gel/celite pad, washed with EtOAc and the resulting mixture was then analyzed by ^{1}H-NMR to quantify the formation of allylic amine **3b**.

Chloramine T M	Rate (mmol/min)
0.001250	0.000016
0.003125	0.000075
0.006250	0.000177
0.009375	0.000219
0.012500	0.000260
0.015625	0.000264

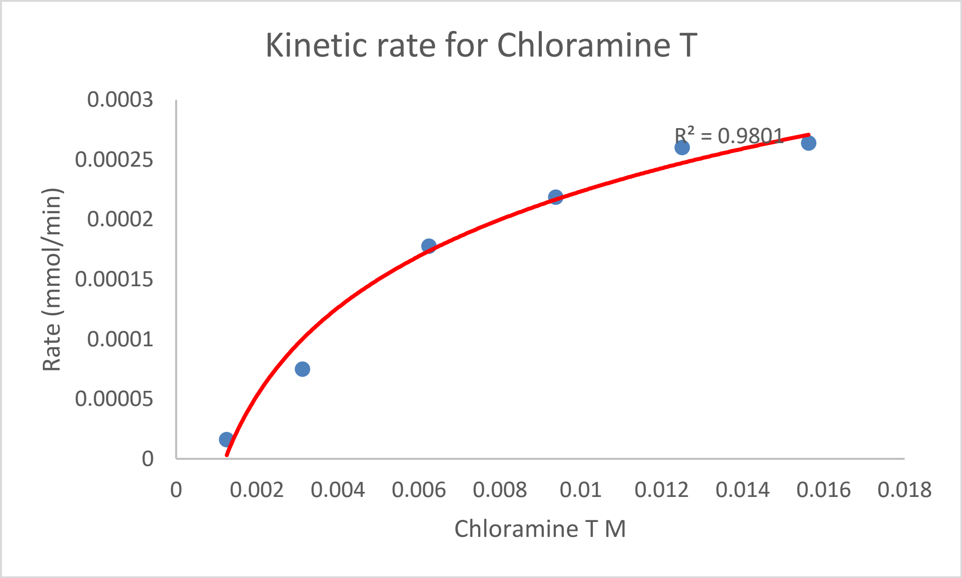

Figure S2. Kinetic rate for chloramine T plot.

Reaction rates in kinetic experiments were monitored up to 40-50% conversion to allylic amine **3b**. The reaction order was determined for alkene (Figure S1) and chloramine T (Figure S2) and it was found that the reaction is first order in [alkene] while [chloramine T] displayed saturation kinetics. These results support the proposed mechanism where $V_2O_3Dipic_2(HMPA)_2$ undergoes fast reaction with chloramine T to form complex **1** prior to alkene coordination. The observed saturation for [Chloramine T] and first order for [alkene] is in agreement with alkene coordination on the vanadoxaziridine metal center after formation of **1**. This also indicates that alkene coordination or aminovanadation may be the rate-limiting-step.

Ligand competition kinetic experiment: The same reaction as the one described for the general synthesis of allylic amine **3** was performed with 0.1 mmol of **2a** and 2, 5, and 10 of excess HMPA. The reaction was allowed to run for 16 hours and a small aliquot (16 µL) of each reaction mixture was retrieved and diluted to 0.5 mL with $CDCl_3$ for quantitative ^{1}H-NMR analysis. The reaction conversion to allylic amine was not significantly inhibited by the presence of HMPA. The reaction at 50 equiv. of HMPA did show significantly lower conversion, thus indicating the catalyst undergoes saturation in very high excess of HMPA. Overall, these results indicate that HMPA-alkene ligand exchange is reversible and fast for this process.

Competition experiments: These experiments were designed using deuterated α-methyl styrenes 2a1-d_2 and 2a2-d_3 were designed to address the nature of the proposed catalytic cycle. A competition experiment between 2a and 2a1-d_2 would provide secondary isotope effect data and a competition experiment between 2a and 2a2-d_3 would provide primary isotope effect data. Thus, 1:1 (4 equiv. each) mixtures of 2a and 2a1-d_2 was reacted under general method to provide the respective allylic amines 3a and 2a and 3a1-d_2. After correcting for 2a1-d_2 deuterium purity (96%-d), we obtained an inverse secondary k_H/k_D of 0.88. This result indicates that the rate-determining-step may be the aminovanadation to provide intermediate **A**. Moreover, 1:1 (4 equiv. each) mixtures of 2a and 2a2-d_2 was reacted under general method to provide the respective allylic amines 3a and 2a and 3a2-d_2. After correcting for 2a2-d_3 deuterium purity (95%-d), we obtained a primary k_H/k_D of 1.01. This indicates that the elimination step is reasonably fast, data that correlates very well with the results observed across α-methyl-alkyl alkenes.

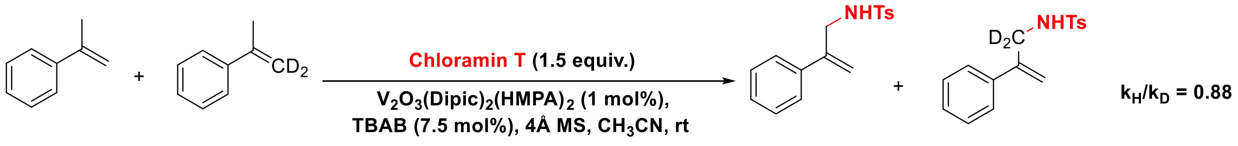

2a **2a1-d$_2$** **3a**, 42.4% **3a1-d$_2$**, 48.2%

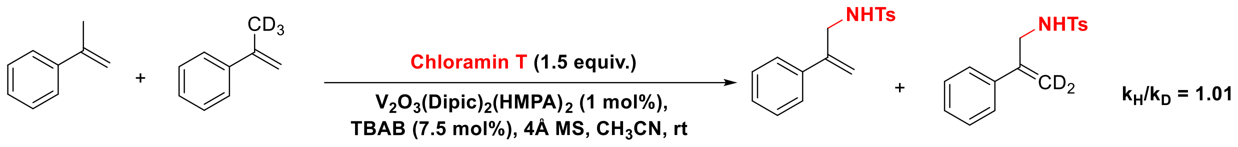

2a **2a2-d$_3$** **3a**, 49.3% **3a2-d$_2$**, 48.8%

General Procedure for competition experiments with deuterated-alkenes: In a 16 mL oven-dried and flushed with nitrogen vial packed with a magnetic stirrer, dry chloramine T (1.5 mmol, 1.5 equiv.) was mixed in CH_3CN (8 mL) and then 4Å MS (200 mg, 200mg/mmol), TBAB (0.075 mmol, 7.5 mol%), $V_2O_3Dipic_2(HMPA)_2$ (0.01 mmol, 1 mol%), alkene **2a** (0.5 mmol, 0.5 equiv.), and alkene **2a1-d$_2$** (0.5 mmol, 0.5 equiv.) were added. The reaction was then quickly flashed with nitrogen and allowed to stir at rt for 8 hours. The crude was then filtered by a 1:1 silica gel/celite pad and the resulting mixture was then purified by silica gel chromatography and the ratio of allylic amines **3a** and **3a1-d$_2$** was calculated from the resulting ^{1}H-NMR spectra.

Hammett experiments:

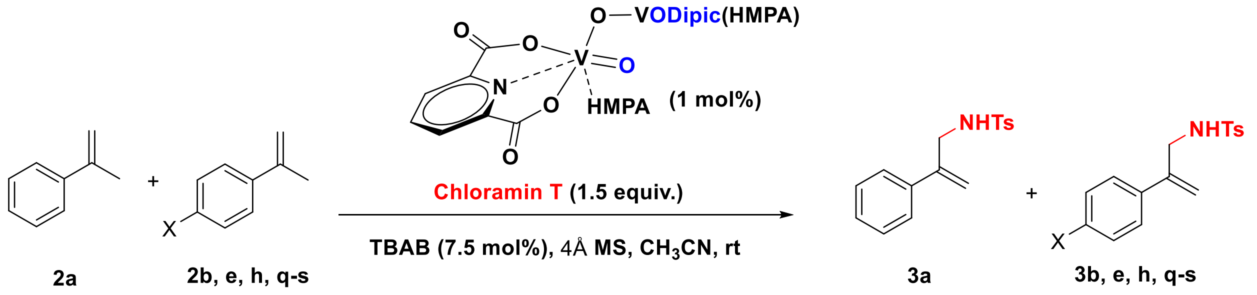

General Procedure for Hammett competition experiments: In a 16 mL oven-dried and flushed with nitrogen vial packed with a magnetic stirrer, dry chloramine T (1.5 mmol, 1.5 equiv.) was mixed in CH_3CN (8 mL) and then 4Å MS (200 mg, 200mg/mmol), TBAB (0.075 mmol, 7.5 mol%), $V_2O_3Dipic_2(HMPA)_2$ (0.01 mmol, 1 mol%), alkene **2a** (1 mmol, 1 equiv.), and alkene **2b** (1 mmol, 1 equiv.) were added. The reaction was then quickly flashed with nitrogen and allowed to stir at rt for 2 hours. The crude was then filtered by a 1:1 silica gel/celite pad and the resulting mixture was then purified by silica gel chromatography and the ratio of allylic amines **3a** and **3b** was calculated from the resulting ^{1}H-NMR spectra. The same procedure was performed separately for alkenes **2e**, **2h**, **2q**, **2r**, and **2s**. Hammett experiments were analyzed as crudes and by purification and the results were equal.

Entry	X	K_X/K_H	$Log(K_X/K_H)$	constant
1	H	1	0	0
2	Me	1.87	0.2718	-0.17
3	t-Bu	2.51	0.3997	-0.20
4	MeO	3.32	0.5211	-0.27
5	Cl	0.26	-0.5850	0.23
6	I	0.35	-0.4559	0.18
7	F	0.84	-0.0757	0.06

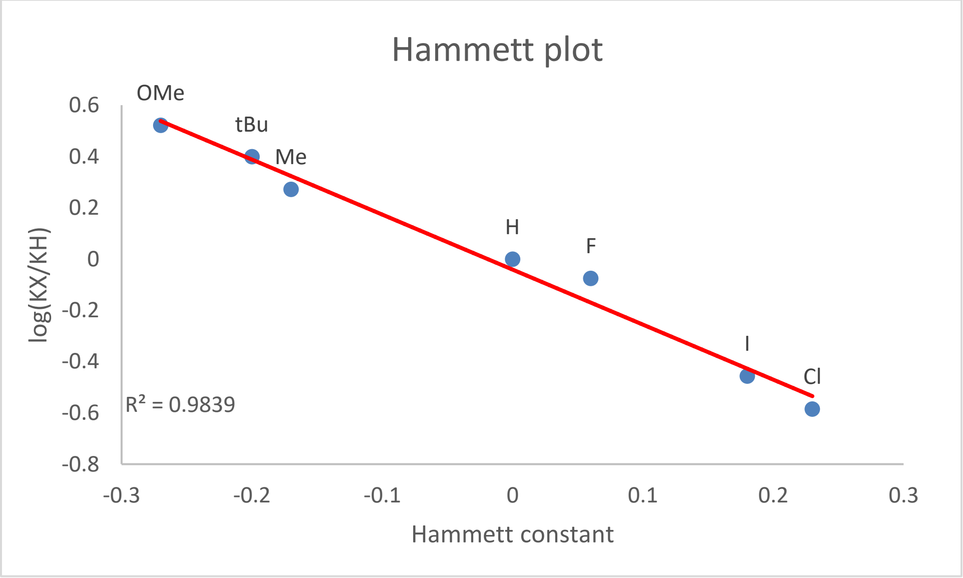

Figure S3. Hammet plot.

The Hammett experiments provided a ρ = -2.15, indicating that electron rich α-methylstyrenes lead to increased rates of allylic amination. The high ρ value correlates well with the development of a positive charge in the transition state, leading to intermediate **A**. These results are also in agreement with a *syn*-aminovanadation step across α-methylstyrenes to form intermediate **A** as the rate-determining-step.

Control experiments:
Experiments in the absence of $V_2O_3Dipic_2(HMPA)_2$ did not provide amino alcohol **3**. Moreover, no combination of chloramine T and PTC were able to produce allylic amine **3**. The reaction under catalytic conditions in the absence of PTC achieves moderate conversion for **3a** (32%) after 96h.

Below there is a list of some other control experiments that further validate the proposed mechanism:

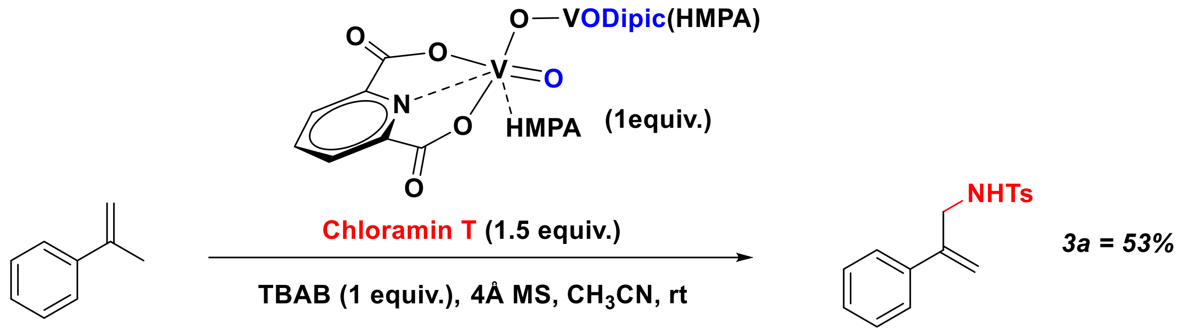

The reaction with stoichiometric amounts of $V_2O_3Dipic_2(HMPA)_2$ proved to work in moderate yield. $V_2O_3Dipic_2(HMPA)_2$ is a white solid and vanadoxaziridine **1** (active catalyst) is a light yellow solid, thus upon mixing $V_2O_3Dipic_2(HMPA)_2$ with chloramine T we did observe a subtle change in color. However, the liquid/solid interface was greatly disturbed, and the increased heterogeneity hurt the reaction conversion. The conversion to **3** validates the required formation of active catalyst **1** to promote the observed reaction.

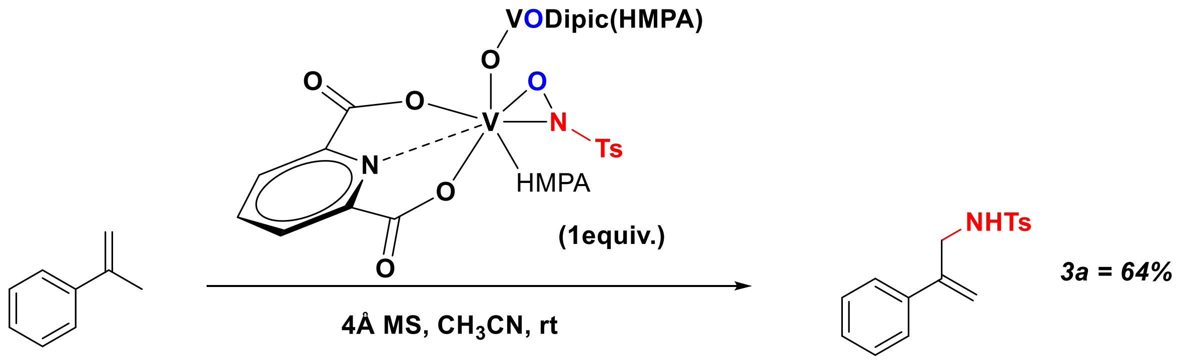

The reaction with stoichiometric amounts of **1** provided the expected product in moderate yield. Despite the increased solubility of **1**, the reaction was cloudy and after 3 h the reaction was completely heterogeneous. Despite the low conversion due to decomposition pathways, this experiment further proves that the reaction does not go through a LA-activated pathway neither

PTC plays a role in the actual transformation. Thus, providing further evidence for the proposed pathway and the first report of a metallooxaziridine-mediated allylic amination reaction.

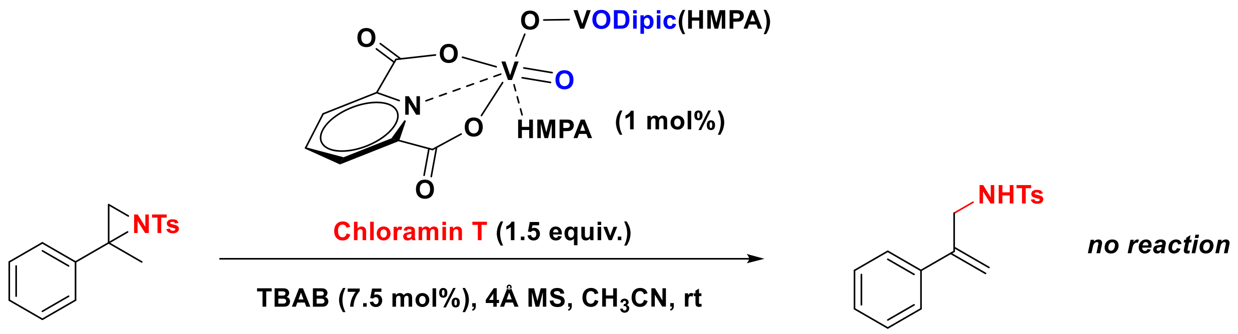

Under the reaction conditions, 2-methyl-2-phenyl-1-tosylaziridine does not react and no traces of allylic amine **3a** are observed in the crude reaction mixture. This clearly indicated that aziridine is not an intermediate for this allylic amination reaction.

Intermediate A characterization:
We prepared a stoichiometric reaction (1 equiv. of **1** + 1 equiv. α-methylstyrene) in CH_3CN under anhydrous conditions, and we were able to observe intermediate **A** by mass spectrometry (**ESI-MS** *m/z* (M+H) = 947.1, observed = 947.0). ^{1}H-NMR analysis of **A** revealed characteristic resonances for the proposed vanadoisoxazolidine **A**. However, intermediate is very reactive and undergoes fast elimination to form allylic amine **3a**. Efforts to afford stable intermediate **A** from substrates without α-protons has not been successful thus far.

List of other substrates that failed to react in comparable yields:

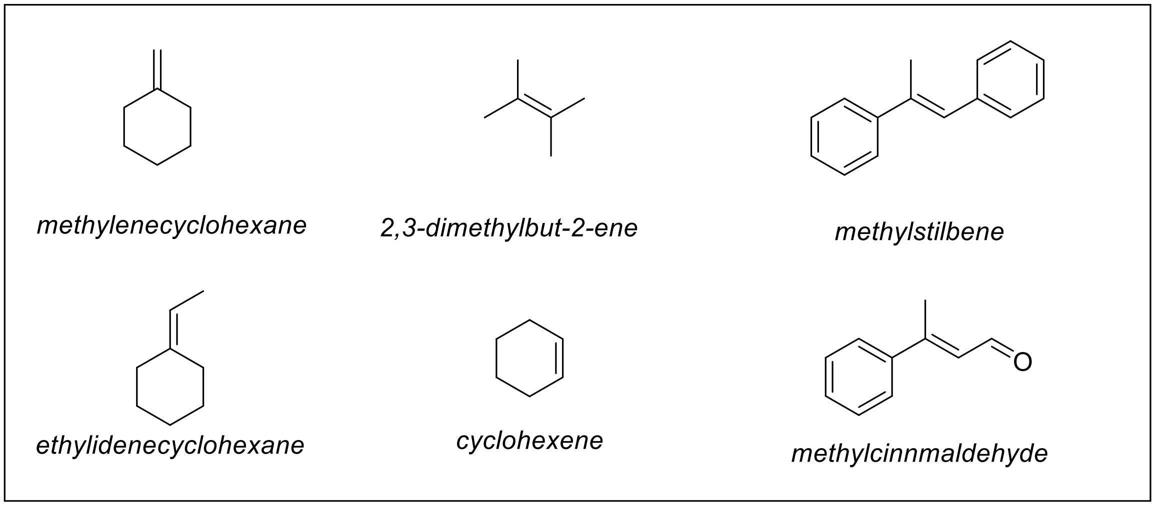

B. SYNTHESIS OF VANADOXAZIRIDINE 1:

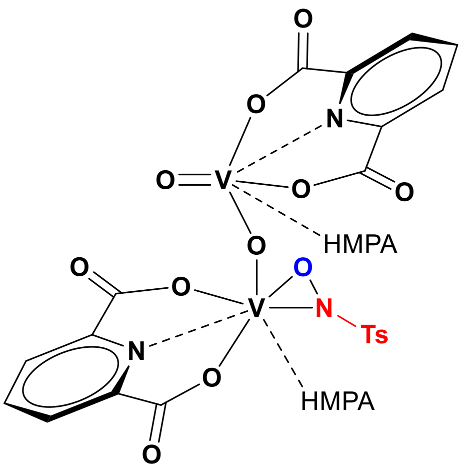

N-tosyl-Dipic$_2$(HMPA)$_2$vanadoxaziridine (1): V_2O_5 (1.81 g, 10 mmol, 1 equiv.) is mixed in CH_2Cl_2 (0.1M, 100 mL) with Dipic (3.34 g, 20 mmol, 1 equiv.) and HMPA (3.48 mL, 20 mmol, 1 equiv.) and the resulting heterogeneous mixture allowed to react at rt for 2 hours. The filtrate was then washed with CH_2Cl_2 and dried under vacuum to obtain residue V_2O_3Dipic$_2$(HMPA)$_2$. Dry chloramine T (2.27 g, 10 mmol, 1 equiv.) is mixed in MeOH (0.1M, 100 mL) with V_2O_3Dipic$_2$(HMPA)$_2$ and the resulting heterogeneous mixture is then stirred vigorously at rt for 8h. The reaction is then allowed to settle, and the supernatant is removed and another portion of MeOH (100 mL) is added the reaction is stirred at rt for another hour. This step was repeated two more times to provide pure vanadoxaziridine **1** as light-yellow crystals (5,343 g, 5.3 mmol, 53% yield). **MP**: 262-264°C. **^{1}H-NMR** (400 MHz, CD_3OD): δ 8.24-8.22 (m, 4H), 8.08 (t, *J* = 8.8 Hz, 2H), 7.68 (d, *J* = 8.2 Hz, 2H), 7.24 (d, *J* = 8.2, 2H), 2.54 (s, 18H), 2.52 (s, 18H), 2.30 (s, 3H). **^{13}C-NMR {^{1}H}** (100 MHz, CD_3OD): δ 166.1, 147.8, 142.7, 140.9, 139.5, 129.1, 127.5, 125.8, 35.7, 20.1 ppm. **ESI-MS** *m/z* (rel int): (pos) 1008.2 ($[M+H]^+$, 100); (neg) 1006.2 ($[M-H]^-$, 100). **HRMS** (ESI) m/z: $[M+H]^+$ Calcd for: $C_{33}H_{50}N_9O_{15}P_2SV_2^+$: 1008.1496, found: 1008.1485. Absolute difference (ppm): 1.12.

C. SYNTHESIS OF AMINOALCOHOLS FROM TABLES 2 AND 3:

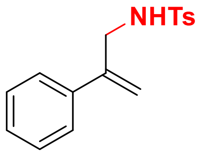

4-methyl-N-(2-phenylallyl)benzenesulfonamide (3a): Alkene **2a** (0.1 mmol) reacted under the general method to produce allylic amine **3a** as a white solid (27 mg, 0.095 mmol, 95%).[1] **TLC**: R_f: 0.32 (2:1 heptanes/EtOAc). **IR** (thin film): υ 3280, 3050, 2926, 1588, 1364, 1153, 817, 664, 548 cm^{-1}. **^{1}H-NMR** (400 MHz, $CDCl_3$): δ 7.72 (d, *J* = 8.0 Hz, 2H), 7.30-7.21 (m, 7H), 5.37 (d, *J* = 0.8 Hz, 1H), 5.21 (d, *J* = 0.8 Hz, 1H), 4.57 (t, *J* = 6.2 Hz, 1H), 3.98 (ddd, *J* = 6.2, 1.6, 0.8 Hz, 2H), 2.44 (s, 3H). **^{13}C-NMR {^{1}H}** (100 MHz, $CDCl_3$): δ 143.5, 142.9, 137.9, 136.7, 129.8, 128.7, 128.2, 127.3, 126.1, 115.1, 47.1, 21.6 ppm. **ESI-MS** *m/z* (rel int): (pos) 288.1 ([M+H]$^+$, 100); (neg) 286.1 ([M–H]$^-$, 100). **HRMS** (ESI) m/z: [M+H]$^+$ Calcd for: $C_{16}H_{18}NO_2S^+$: 288.1053, found: 288.1053. Absolute difference (ppm): 0.

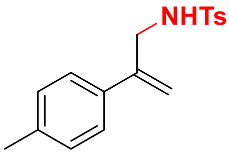

4-methyl-N-(2-(p-tolyl)allyl)benzenesulfonamide (3b): Alkene **2b** (0.1 mmol) reacted under the general method to produce allylic amine **3b** as a white solid (28 mg, 0.093 mmol, 93%).[2] **TLC**: R_f: 0.32 (2:1 heptanes/EtOAc). **MP**: 75-76 °C. **IR** (thin film): υ 3284, 3044, 2988, 1564, 1353, 1148, 816, 648, 553 cm^{-1}. **^{1}H-NMR** (400 MHz, $CDCl_3$): δ 7.71 (d, *J* = 8.0 Hz, 2H), 7.28 (d, *J* = 8.0 Hz, 2H), 7.14-7.07 (m, 4H), 5.33 (s, 1H), 5.14 (s, 1H), 4.59-4.52 (m, 1H), 3.96 (d, *J* = 6.2 Hz, 2H), 2.44 (s, 3H), 2.33 (s, 3H). **^{13}C-NMR {^{1}H}** (100 MHz, $CDCl_3$): δ 143.5, 142.5, 138.1, 136.6, 134.8, 129.7, 129.2, 127.2, 125.8, 114.4, 47.1, 21.6, 21.1 ppm. **ESI-MS** *m/z* (rel int): (pos) 302.1 ([M+H]$^+$, 100); (neg) 300.1 ([M–H]$^-$, 100). **HRMS** (ESI) m/z: [M+H]$^+$ Calcd for: $C_{17}H_{20}NO_2S^+$: 302.1209, found: 302.1209. Absolute difference (ppm): 0.

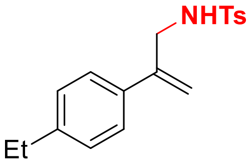

N-(2-(4-ethylphenyl)allyl)-4-methylbenzenesulfonamide (3c): Alkene **2c** (0.1 mmol) reacted under the general method to produce allylic amine **3c** as a white solid (29 mg, 0.091 mmol, 91%). **TLC**: R_f: 0.32 (2:1 heptanes/EtOAc). **MP**: 82-83 °C. **IR** (thin film): υ 3262, 3044, 2980, 1582, 1353, 1135, 813, 662, 540 cm^{-1}. **^{1}H-NMR** (400 MHz, $CDCl_3$): δ 7.72 (d, *J* = 8.0 Hz, 2H), 7.30 (d, *J* = 8.0 Hz, 2H), 7.16-7.10 (m, 4H), 5.33 (d, *J* = 0.8 Hz, 1H), 5.14 (d, *J* = 0.8 Hz, 1H), 4.46 (t, *J* = 6.2 Hz, 1H), 3.98 (ddd, *J* = 6.2, 1.2, 0.8 Hz, 2H), 2.62 (q, *J* = 7.1 Hz, 2H), 2.44 (s, 3H), 1.23 (t, *J* = 7.1 Hz, 3H). **^{13}C-NMR {^{1}H}** (100 MHz, $CDCl_3$): δ 144.5, 143.5, 142.6, 136.8, 135.1, 129.7, 128.1, 127.2, 126.1, 114.4, 47.1, 28.5, 21.6, 15.5 ppm. **ESI-MS** *m/z* (rel int): (pos) 316.2 ([M+H]$^+$, 100); (neg) 314.2 ([M–H]$^-$, 100). **HRMS** (ESI) m/z: [M+H]$^+$ Calcd for: $C_{18}H_{22}NO_2S^+$: 316.1366, found: 316.1368. Absolute difference (ppm): 0.63.

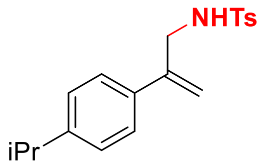

N-(2-(4-isopropylphenyl)allyl)-4-methylbenzenesulfonamide (3d): Alkene **2d** (0.1 mmol) reacted under the general method to produce allylic amine **3d** as a white solid (31 mg, 0.094 mmol, 94%). **TLC**: R_f : 0.36 (2:1 heptanes/EtOAc). **MP**: 84-86 °C. **IR** (thin film): υ 3293, 3064, 2971, 1564, 1348, 1144, 824, 682, 553 cm^{-1}. **^{1}H-NMR** (400 MHz, $CDCl_3$): δ 7.72 (d, *J* = 8.0 Hz, 2H), 7.30 (d, *J* = 8.0 Hz, 2H), 7.18-7.13 (m, 4H), 5.34 (s, 1H), 5.14 (s, 1H), 4.43 (t, *J* = 6.2 Hz, 1H), 3.99 (d, *J* = 6.2 Hz, 2H), 2.88 (heptet, *J* = 7.1 Hz, 1H), 2.44 (s, 3H), 1.25 (d, *J* = 7.1 Hz, 6H). **^{13}C-NMR {^{1}H}** (100 MHz, $CDCl_3$): δ 149.1, 143.5, 142.6, 136.8, 135.2, 129.7, 127.3, 126.6, 125.8, 114.4, 47.1, 33.8, 23.9, 21.5 ppm. **ESI-MS** *m/z* (rel int): (pos) 330.2 ($[M+H]^+$, 100); (neg) 328.2 ($[M-H]^-$, 100). **HRMS** (ESI) m/z: $[M+H]^+$ Calcd for: $C_{19}H_{24}NO_2S^+$: 330.1522, found: 330.1526. Absolute difference (ppm): 1.21.

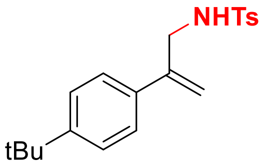

N-(2-(4-(tert-butyl)phenyl)allyl)-4-methylbenzenesulfonamide (3e): Alkene **2e** (0.1 mmol) reacted under the general method to produce allylic amine **3e** as a white solid (28 mg, 0.090 mmol, 90%).[2] **TLC**: R_f : 0.36 (2:1 heptanes/EtOAc). **IR** (thin film): υ 3850, 3203, 2905, 1744, 1512, 1313, 1124, 943 cm^{-1}. **^{1}H-NMR** (400 MHz, $CDCl_3$): δ 7.72 (d, *J* = 8.0 Hz, 2H), 7.31-7.25 (m, 4H), 7.18 (d, *J* = 8.0 Hz, 2H), 5.35 (d, *J* = 0.8 Hz, 1H), 5.14 (d, *J* = 0.8 Hz, 1H), 4.53 (s, 1H), 3.98 (dd, *J* = 1.6, 0.8 Hz, 2H), 2.44 (s, 3H), 1.31 (s, 9H). **^{13}C-NMR {^{1}H}** (100 MHz, $CDCl_3$): δ 151.3, 143.4, 142.5, 136.8, 134.8, 129.7, 127.3, 125.7, 125.5, 114.4, 47.0, 34.5, 31.3, 21.5 ppm. **ESI-MS** *m/z* (rel int): (pos) 344.2 ($[M+H]^+$, 100); (neg) 342.2 ($[M-H]^-$, 100). **HRMS** (ESI) m/z: $[M+H]^+$ Calcd for: $C_{20}H_{26}NO_2S^+$: 344.1679, found: 344.1673. Absolute difference (ppm): 1.74.

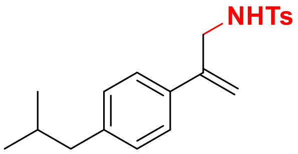

N-(2-(4-isobutylphenyl)allyl)-4-methylbenzenesulfonamide (3f): Alkene **2f** (0.1 mmol) reacted under the general method to produce allylic amine **3f** as a white solid (30 mg, 0.089 mmol, 89%). **TLC**: R_f : 0.39 (2:1 heptanes/EtOAc). **MP**: 96-97 °C. **IR** (thin film): υ 3275, 3053, 2948, 1564, 1380, 1135, 812, 646, 540 cm^{-1}. **^{1}H-NMR** (400 MHz, $CDCl_3$): δ 7.71 (d, *J* = 8.0 Hz, 2H), 7.29 (d, *J* = 8.0 Hz, 2H), 7.14 (d, *J* = 8.0 Hz, 2H), 7.05 (d, *J* = 8.0 Hz, 2H), 5.35 (s, 1H), 5.14 (s, 1H), 4.42 (t, *J* = 6.0 Hz, 1H), 3.98 (d, *J* = 6.0 Hz, 2H), 2.45-2.44 (m, 5H), 1.88-1.81 (m, 1H), 0.91 (d, *J* = 7.1 Hz, 6H). **^{13}C-NMR {^{1}H}** (100 MHz, $CDCl_3$): δ 143.5, 142.6, 141.9, 136.8, 135.1, 129.6, 129.3, 127.2, 125.8, 114.4, 47.1, 45.1, 30.1, 22.4, 21.5 ppm. **ESI-MS** *m/z* (rel int): (pos) 344.2 ($[M+H]^+$,

100); (neg) 342.2 ([M–H]$^-$, 100). **HRMS** (ESI) m/z: [M+H]$^+$ Calcd for: $C_{20}H_{26}NO_2S^+$: 344.1679, found: 344.1674. Absolute difference (ppm): 1.45.

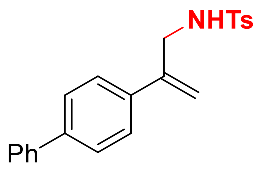

N-(2-([1,1'-biphenyl]-4-yl)allyl)-4-methylbenzenesulfonamide (3g): Alkene **2g** (0.1 mmol) reacted under the general method to produce allylic amine **3g** as a white solid (35 mg, 0.097 mmol, 97%).[3] **TLC**: R_f: 0.39 (2:1 heptanes/EtOAc). **IR** (thin film): υ 3284, 3005, 2962, 1580, 1364, 1153, 812, 671, 542 cm^{-1}. **^{1}H-NMR** (400 MHz, $CDCl_3$): δ 7.74 (d, *J* = 8.0 Hz, 2H), 7.53 (d, *J* = 8.0 Hz, 2H), 7.50 (d, *J* = 8.0 Hz, 2H), 7.43 (t, *J* = 7.3 Hz, 2H), 7.35-7.25 (m, 5H), 5.43 (s, 1H), 5.23 (s, 1H), 4.53 (s, 1H), 4.02 (d, *J* = 6.2 Hz, 2H), 2.44 (s 3H). **^{13}C-NMR {^{1}H}** (100 MHz, $CDCl_3$): δ 143.5, 142.4, 141.0, 140.3, 136.8, 136.6, 129.7, 128.9, 128.8, 127.5, 127.3, 127.0, 126.5, 115.3, 47.1, 21.6 ppm. **ESI-MS** *m/z* (rel int): (pos) 364.1 ([M+H]$^+$, 100); (neg) 362.1 ([M–H]$^-$, 100). **HRMS** (ESI) m/z: [M+H]$^+$ Calcd for: $C_{22}H_{22}NO_2S^+$: 364.1366, found: 364.1369. Absolute difference (ppm): 0.82.

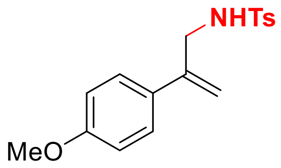

N-(2-(4-methoxyphenyl)allyl)-4-methylbenzenesulfonamide (3h): Alkene **2h** (0.1 mmol) reacted under the general method to produce allylic amine **3h** as a colorless oil (29 mg, 0.092 mmol, 92%).[2] **TLC**: R_f : 0.40 (1:1 heptanes/EtOAc). **IR** (thin film): υ 3244, 3017, 2962, 2953, 1482, 1362, 1094, 813 cm^{-1}.**^{1}H-NMR** (400 MHz, $CDCl_3$): δ 7.72 (d, *J* = 8.0 Hz, 2H), 7.32 (d, *J* = 8.0 Hz, 2H), 7.18 (d, *J* = 8.0 Hz, 2H), 6.80 (d, *J* = 8.0 Hz, 2H), 5.29 (d, *J* = 0.8 Hz, 1H), 5.09 (d, *J* = 0.8 Hz, 1H), 4.40 (t, *J* = 6.2 Hz, 1H), 3.98 (dd, *J* = 6.2, 1.6 Hz, 2H), 3.81 (s, 3H), 2.44 (s, 3H). **^{13}C-NMR {^{1}H}** (100 MHz, $CDCl_3$): δ 159.6, 143.5, 142.1, 136.7, 129.7, 129.6, 127.3, 127.2, 114.0, 113.6, 55.3, 47.2, 21.6 ppm. **ESI-MS** *m/z* (rel int): (pos) 318.2 ([M+H]$^+$, 100); (neg) 316.2 ([M–H]$^-$, 100). **HRMS** (ESI) m/z: [M+H]$^+$ Calcd for: $C_{17}H_{20}NO_3S^+$: 318.1158, found: 318.1154. Absolute difference (ppm): 1.26.

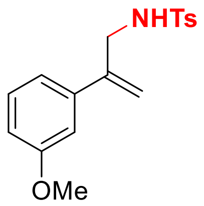

N-(2-(3-methoxyphenyl)allyl)-4-methylbenzenesulfonamide (3i): Alkene **2i** (0.1 mmol) reacted under the general method to produce allylic amine **3i** as a pale yellow solid (29 mg, 0.09 mmol, 90%). **TLC**: R_f : 0.42 (1:1 heptanes/EtOAc). **MP**: 116-117 °C. **IR** (thin film): υ 3232, 3018, 2962, 2935, 1488, 1326, 1094, 817 cm^{-1}.**^{1}H-NMR** (400 MHz, $CDCl_3$): δ 7.72 (d, *J* = 8.0 Hz, 2H), 7.28 (d, *J* = 8.0 Hz, 2H), 7.72 (t, *J* = 7.1 Hz, 1H), 6.84-6.80 (m, 2H), 6.77 (s, 1H), 5.37 (s, 1H), 5.21 (s,

1H), 4.46 (t, *J* = 6.2 Hz, 1H), 3.98 (d, *J* = 6.2 Hz, 2H), 3.78 (s, 3H), 2.44 (s, 3H). **^{13}C-NMR {^{1}H}** (100 MHz, $CDCl_3$): δ 159.7, 143.5, 142.8, 139.4, 136.8, 129.7, 129.5, 127.2, 118.5, 115.4, 113.6, 112.0, 55.3, 47.1, 21.5 ppm. **ESI-MS** *m/z* (rel int): (pos) 318.2 ($[M+H]^+$, 100); (neg) 316.2 ($[M-H]^-$, 100). **HRMS** (ESI) m/z: $[M+H]^+$ Calcd for: $C_{17}H_{20}NO_3S^+$: 318.1158, found: 318.1154. Absolute difference (ppm): 1.26.

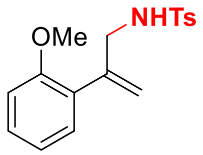

N-(2-(2-methoxyphenyl)allyl)-4-methylbenzenesulfonamide (3j): Alkene **2j** (0.1 mmol) reacted under the general method to produce allylic amine **3j** as a light yellow solid (28 mg, 0.088 mmol, 88%).[4] **TLC**: R_f : 0.40 (1:1 heptanes/EtOAc). **IR** (thin film): υ 3240, 3016, 2991, 2953, 1480, 1324, 1093, 811 cm^{-1}. **^{1}H-NMR** (400 MHz, $CDCl_3$): δ 7.65 (d, *J* = 8.0 Hz, 2H), 7.27-7.25 (m, 3H), 6.93-6.91 (m, 1H), 6.88-6.86 (m, 1H), 6.78 (d, *J* = 6.8 Hz, 1H), 5.25 (d, *J* = 0.8 Hz, 1H), 5.10 (d, *J* = 0.8 Hz, 1H), 4.57 (s, 1H), 4.00 (d, *J* = 5.8 Hz, 2H), 3.71 (s, 3H), 2.42 (s 3H). **^{13}C-NMR {^{1}H}** (100 MHz, $CDCl_3$): δ 156.3, 143.8, 143.1, 137.1, 130.5, 129.5, 129.4, 128.3, 127.2, 120.9, 117.1, 110.6, 55.3, 47.7, 21.5 ppm. **ESI-MS** *m/z* (rel int): (pos) 318.2 ($[M+H]^+$, 100); (neg) 316.2 ($[M-H]^-$, 100). **HRMS** (ESI) m/z: $[M+H]^+$ Calcd for: $C_{17}H_{20}NO_3S^+$: 318.1158, found: 318.1154. Absolute difference (ppm): 1.26.

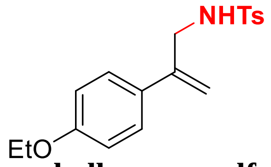

N-(2-(4-ethoxyphenyl)allyl)-4-methylbenzenesulfonamide (3k): Alkene **2k** (0.1 mmol) reacted under the general method to produce allylic amine **3k** as a white solid (29 mg, 0.089 mmol, 89%). **TLC**: R_f : 0.42 (1:1 heptanes/EtOAc). **MP**: 124-126 °C. **IR** (thin film): υ 3239, 3005, 2960, 2953, 1464, 1335, 1088, 811 cm^{-1}. **^{1}H-NMR** (400 MHz, $CDCl_3$): δ 7.72 (d, *J* = 8.0 Hz, 2H), 7.30 (d, *J* = 8.0 Hz, 2H), 7.18 (d, *J* = 8.0 Hz, 2H), 6.80 (d, *J* = 8.0 Hz, 2H), 5.29 (s, 1H), 5.08 (s, 1H), 4.39 (s, 1H), 4.02 (q, *J* = 6.4 Hz, 2H), 3.96 (d, *J* = 6.2 Hz, 2H), 2.44 (s, 3H), 1.41 (t, *J* = 6.4 Hz, 3H). **^{13}C-NMR {^{1}H}** (100 MHz, $CDCl_3$): δ 159.0, 143.5, 142.1, 136.8, 132.2, 129.9, 128.5, 127.3, 114.5, 113.5, 60.4, 47.2, 21.6, 14.8 ppm. **ESI-MS** *m/z* (rel int): (pos) 332.2 ($[M+H]^+$, 100); (neg) 330.2 ($[M-H]^-$, 100). **HRMS** (ESI) m/z: $[M+H]^+$ Calcd for: $C_{18}H_{22}NO_3S^+$: 332.1315, found: 332.1311. Absolute difference (ppm): 1.20.

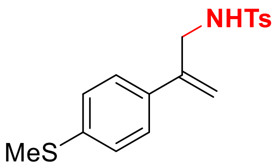

4-methyl-N-(2-(4-(methylthio)phenyl)allyl)benzenesulfonamide (3l): Alkene **2l** (0.1 mmol) reacted under the general method to produce allylic amine **3l** as a light yellow solid (30 mg, 0.09 mmol, 90%). **TLC**: R_f: 0.48 (1:1 heptanes/EtOAc). **MP**: 128-130 °C. **IR** (thin film): υ 3282, 3051, 2988, 1582, 1364, 1106, 816, 544 cm^{-1}. **^{1}H-NMR** (400 MHz, $CDCl_3$): δ 7.65 (d, *J* = 7.8 Hz, 2H), 7.23 (d, *J* = 7.8 Hz, 2H), 7.10-7.05 (m, 4H), 5.29 (s, 1H), 5.10 (s, 1H), 4.32 (t, *J* = 6.2 Hz, 1H), 3.89 (d, *J* = 6.2 Hz, 2H), 2.41 (s, 3H), 2.38 (s, 3H). **^{13}C-NMR {^{1}H}** (100 MHz, $CDCl_3$): δ 143.6, 142.2, 138.9, 136.7, 134.4, 129.8, 129.6, 127.3, 126.5, 114.8, 47.0, 21.6, 15.6 ppm. **ESI-MS** *m/z* (rel int): (pos) 334.2 ($[M+H]^+$, 100); (neg) 332.2 ($[M–H]^-$, 100). **HRMS** (ESI) m/z: $[M+H]^+$ Calcd for: $C_{17}H_{20}NO_2S_2^+$: 334.0930, found: 334.0933. Absolute difference (ppm): 0.90.

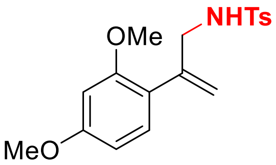

N-(2-(2,4-dimethoxyphenyl)allyl)-4-methylbenzenesulfonamide (3m): Alkene **2m** (0.1 mmol) reacted under the general method to produce allylic alcohol **3m** as a white solid (30 mg, 0.086 mmol, 86%). **TLC**: R_f : 0.32 (1:1 heptanes/EtOAc). **MP**: 97-99 °C. **IR** (thin film): υ 3230, 3023, 2982, 1564, 1364, 1153, 815, 682, 530 cm^{-1}. **^{1}H-NMR** (400 MHz, $CDCl_3$): δ 7.64 (d, *J* = 8.0 Hz, 2H), 7.24 (d, *J* = 8.0 Hz, 2H), 6.84 (d, *J* = 7.1 Hz, 1H), 6.37-6.35 (m, 2H), 5.18 (d, *J* = 0.8 Hz, 1H), 5.05 (d, *J* = 0.8 Hz, 1H), 4.49 (t, *J* = 6.2 Hz, 1H), 3.98 (d, *J* = 6.2 Hz, 2H), 3.80 (s, 3H), 3.67 (s, 3H), 2.42 (s 3H). **^{13}C-NMR {^{1}H}** (100 MHz, $CDCl_3$): δ 161.0, 157.3, 143.3, 143.1, 137.2, 131.0, 129.5, 127.2, 120.9, 116.4, 104.5, 98.5, 55.4, 55.3, 47.9, 21.5 ppm. **ESI-MS** *m/z* (rel int): (pos) 348.1 ($[M+H]^+$, 100); (neg) 346.1 ($[M–H]^-$, 100). **HRMS** (ESI) m/z: $[M+H]^+$ Calcd for: $C_{18}H_{22}NO_4S^+$: 348.1264, found: 348.1269. Absolute difference (ppm): 1.43.

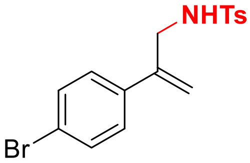

N-(2-(4-bromophenyl)allyl)-4-methylbenzenesulfonamide (3n): Alkene **2n** (0.1 mmol) reacted under the general method to produce allylic amine **3n** as a white solid (33 mg, 0.090 mmol, 90%).[2] **TLC**: R_f: 0.45 (2:1 heptanes/EtOAc). **IR** (thin film): υ 3253, 3023, 2932, 1580, 1364, 1148, 817, 671 cm^{-1}. **^{1}H-NMR** (400 MHz, $CDCl_3$): δ 7.71 (d, *J* = 8.0 Hz, 2H), 7.41 (d, *J* = 8.0 Hz, 2H), 7.28 (d, *J* = 8.0 Hz, 2H), 7.10 (d, *J* = 8.0 Hz, 2H), 5.38 (s, 1H), 5.23 (s, 1H), 4.49 (t, *J* = 6.2 Hz, 1H), 3.96 (d, *J* = 6.2 Hz, 2H), 2.44 (s 3H). **^{13}C-NMR {^{1}H}** (100 MHz, $CDCl_3$): δ 143.7, 142.1, 136.7, 131.6, 129.7, 127.7, 127.2, 127.1, 122.6, 116.0, 47.0, 21.6 ppm. **ESI-MS** *m/z* (rel int): (pos) 366.1 ($[M+H]^+$, 100); (neg) 364.1 ($[M–H]^-$, 100). **HRMS** (ESI) m/z: $[M+H]^+$ Calcd for: $C_{16}H_{17}BrNO_2S^+$: 366.0158, found: 366.0154. Absolute difference (ppm): 1.09.

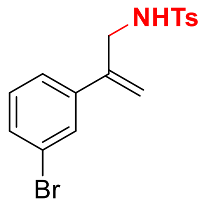

N-(2-(3-bromophenyl)allyl)-4-methylbenzenesulfonamide (3o): Alkene **2o** (0.1 mmol) reacted under the general method to produce allylic amine **3o** as a white solid (32 mg, 0.088 mmol, 88%). **TLC**: R_f: 0.45 (2:1 heptanes/EtOAc). **MP**: 134-136 °C. **IR** (thin film): υ 3230, 3014, 2950, 1555, 1304, 1151, 817, 542 cm^{-1}. **^{1}H-NMR** (400 MHz, $CDCl_3$): δ 7.70 (d, *J* = 8.0 Hz, 2H), 7.41-7.38 (m, 1H), 7.30-7.27 (m, 3H), 7.16-7.14 (m, 2H), 5.36 (s, 1H), 5.26 (s, 1H), 4.56 (t, *J* = 6.0 Hz, 1H), 3.97 (d, *J* = 6.0 Hz, 2H), 2.44 (s 3H). **^{13}C-NMR {^{1}H}** (100 MHz, $CDCl_3$): δ 143.7, 141.8, 140.1, 136.7, 131.1, 130.1, 129.8, 129.2, 127.2, 124.7, 122.7, 116.6, 46.9, 21.6 ppm. **ESI-MS** *m/z* (rel int): (pos) 366.1 ([M+H]$^+$, 100); (neg) 364.1 ([M–H]$^-$, 100). **HRMS** (ESI) m/z: [M+H]$^+$ Calcd for: $C_{16}H_{17}BrNO_2S^+$: 366.0158, found: 366.0159. Absolute difference (ppm): 0.27.

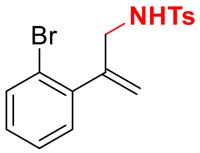

N-(2-(2-bromophenyl)allyl)-4-methylbenzenesulfonamide (3p): Alkene **2p** (0.1 mmol) reacted under the general method to produce allylic amine **3p** as a white solid (31 mg, 0.086 mmol, 86%).[5] **TLC**: R_f : 0.46 (2:1 heptanes/EtOAc). **IR** (thin film): υ 3283, 3009, 2950, 1582, 1366, 1151, 816, 673, 521 cm^{-1}. **^{1}H-NMR** (400 MHz, $CDCl_3$): δ 7.72 (d, *J* = 8.0 Hz, 2H), 7.45 (dd, *J* = 7.3, 4.2 Hz, 1H), 7.36 (d, *J* = 8.0 Hz, 2H), 7.18-7.14 (m, 2H), 7.01 (dt, *J* = 7.1, 4.2 Hz, 1H), 5.34 (s, 1H), 5.21 (s, 1H), 4.53 (t, *J* = 6.0 Hz, 1H), 3.97 (d, *J* = 6.0 Hz, 2H), 2.44 (s 3H). **^{13}C-NMR {^{1}H}** (100 MHz, $CDCl_3$): δ 143.0, 141.7, 140.3, 137.1, 133.3, 130.7, 129.8, 129.2, 127.9, 124.7, 122.7, 118.8, 46.8, 21.5 ppm. **ESI-MS** *m/z* (rel int): (pos) 366.1 ([M+H]$^+$, 100); (neg) 364.1 ([M–H]$^-$, 100). **HRMS** (ESI) m/z: [M+H]$^+$ Calcd for: $C_{16}H_{17}BrNO_2S^+$: 366.0158, found: 366.0154. Absolute difference (ppm): 1.09.

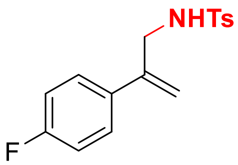

N-(2-(4-fluorophenyl)allyl)-4-methylbenzenesulfonamide (3q): Alkene **2q** (0.1 mmol) reacted under the general method to produce allylic amine **3q** as a white solid (28 mg, 0.091 mmol, 91%).[6] **TLC**: R_f : 0.50 (2:1 heptanes/EtOAc). **MP**: 122-123 °C. **IR** (thin film): υ 3382, 3014, 2962, 1580, 1384, 1205, 817, 802 cm^{-1}. **^{1}H-NMR** (400 MHz, $CDCl_3$): δ 7.70 (d, *J* = 8.0 Hz, 2H), 7.28 (d, *J* = 8.0 Hz, 2H), 7.21 (d, *J* = 8.0 Hz, 2H), 6.96 (t, *J* = 8.0 Hz, 2H), 5.32 (s, 1H), 5.19 (s, 1H), 4.56 (t, *J* = 6.2 Hz, 1H), 3.96 (d, *J* = 6.2 Hz, 2H), 2.44 (s 3H). **^{13}C-NMR {^{1}H}** (100 MHz, $CDCl_3$): δ 162.3 (d, *J* = 246 Hz), 143.6, 141.9, 136.7, 133.9 (d, *J* = 4 Hz), 129.7, 127.9, 127.8 (d, *J* = 8 Hz), 127.2,

115.3 (d, J = 26 Hz), 47.1, 21.5 ppm. **ESI-MS** *m/z* (rel int): (pos) 306.1 ($[M+H]^+$, 100); (neg) 304.1 ($[M-H]^-$, 100). **HRMS** (ESI) m/z: $[M+H]^+$ Calcd for: $C_{16}H_{17}FNO_2S^+$: 306.0959, found: 306.0952. Absolute difference (ppm): 2.28.

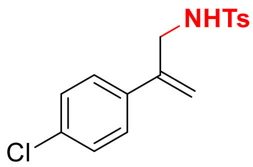

N-(2-(4-chlorophenyl)allyl)-4-methylbenzenesulfonamide (3r): Alkene **2r** (0.1 mmol) reacted under the general method to produce allylic amine **3r** as a white solid (30 mg, 0.092 mmol, 92%).[2] **TLC**: R_f.: 0.50 (2:1 heptanes/EtOAc). **IR** (thin film): υ 3302, 3023, 2962, 1582, 1366, 1153, 812, 673 cm^{-1}. **^{1}H-NMR** (400 MHz, $CDCl_3$): δ 7.71 (d, J = 8.0 Hz, 2H), 7.28 (d, J = 8.0 Hz, 2H), 7.23 (d, J = 8.0 Hz, 2H), 7.18 (d, J = 8.0 Hz, 2H), 5.37 (s, 1H), 5.22 (s, 1H), 4.53 (t, J = 5.3 Hz, 1H), 3.96 (d, J = 5.3 Hz, 2H), 2.44 (s 3H). **^{13}C-NMR {^{1}H}** (100 MHz, $CDCl_3$): δ 143.7, 141.9, 136.7, 136.3, 134.1, 129.7, 128.7, 127.5, 127.2, 115.9, 47.0, 21.6 ppm. **ESI-MS** *m/z* (rel int): (pos) 322.1 ($[M+H]^+$, 100); (neg) 320.1 ($[M-H]^-$, 100). **HRMS** (ESI) m/z: $[M+H]^+$ Calcd for: $C_{16}H_{17}ClNO_2S^+$: 322.0663, found: 322.0660. Absolute difference (ppm): 0.93.

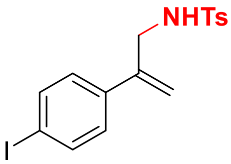

N-(2-(4-iodophenyl)allyl)-4-methylbenzenesulfonamide (3s): Alkene **2s** (0.1 mmol) reacted under the general method to produce allylic amine **3s** as a white solid (37 mg, 0.089 mmol, 89%). **TLC**: R_f : 0.52 (2:1 heptanes/EtOAc). **MP**: 161-162 °C. **IR** (thin film): υ 3382, 3050, 2964, 1580, 1364, 1153, 862, 682 cm^{-1}. **^{1}H-NMR** (400 MHz, $CDCl_3$): δ 7.70 (d, J = 8.0 Hz, 2H), 7.58 (d, J = 8.0 Hz, 2H), 7.27 (d, J = 8.0 Hz, 2H), 6.97 (d, J = 8.0 Hz, 2H), 5.37 (s, 1H), 5.22 (s, 1H), 4.64 (bs, 1H), 3.94 (d, J = 5.3 Hz, 2H), 2.44 (s 3H). **^{13}C-NMR {^{1}H}** (100 MHz, $CDCl_3$): δ 143.6, 142.1, 137.6, 137.4, 136.7, 129.7, 127.9, 127.2, 116.0, 93.8, 46.8, 21.6 ppm. **ESI-MS** *m/z* (rel int): (pos) 414.1 ($[M+H]^+$, 100); (neg) 412.1 ($[M-H]^-$, 100). **HRMS** (ESI) m/z: $[M+H]^+$ Calcd for: $C_{16}H_{17}INO_2S^+$: 414.0019, found: 414.0014. Absolute difference (ppm): 1.21.

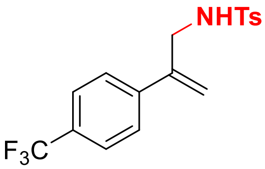

4-methyl-N-(2-(4-(trifluoromethyl)phenyl)allyl)benzenesulfonamide (3t): Alkene **2t** (0.1 mmol) reacted under the general method to produce allylic amine **3t** as a white solid (30 mg, 0.084 mmol, 84%).[6] **TLC**: R_f : 0.52 (2:1 heptanes/EtOAc). **MP**: 122-124 °C. **IR** (thin film): υ 3401, 3018, 2948, 1580, 1364, 1151, 817, 718 cm^{-1}. **^{1}H-NMR** (400 MHz, $CDCl_3$): δ 7.71 (d, J = 8.0 Hz, 2H), 7.54 (d, J = 8.0 Hz, 2H), 7.36 (d, J = 8.0 Hz, 2H), 7.27 (d, J = 8.0 Hz, 2H), 5.47 (s, 1H), 5.33

(s, 1H), 4.43 (s, 1H), 4.02 (d, *J* = 5.3 Hz, 2H), 2.44 (s 3H). **^{13}C-NMR {^{1}H}** (100 MHz, $CDCl_3$): δ 143.7, 142.1, 136.7, 129.9 (d, *J* = 32 Hz), 129.7, 127.2, 126.6, 126.5, 125.5 (d, *J* = 4 Hz), 124.1 (d, *J* = 264 Hz), 117.4, 46.9, 21.5 ppm. **ESI-MS** *m/z* (rel int): (pos) 356.1 ($[M+H]^+$, 100); (neg) 354.1 ($[M-H]^-$, 100). **HRMS** (ESI) m/z: $[M+H]^+$ Calcd for: $C_{17}H_{17}F_3NO_2S^+$: 356.0927, found: 356.0922. Absolute difference (ppm): 1.40.

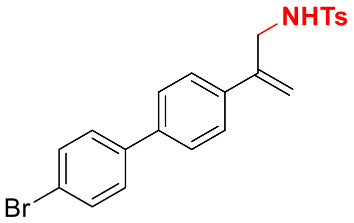

N-(2-(4'-bromo-[1,1'-biphenyl]-4-yl)allyl)-4-methylbenzenesulfonamide (3u): Alkene **2u** (0.1 mmol) reacted under the general method to produce allylic amine **3u** as a white solid (40 mg, 0.09 mmol, 90%). **MP**: 198-199 °C. **TLC**: R_f: 0.48 (2:1 heptanes/EtOAc). **IR** (thin film): υ 3320, 3014, 2944, 1519, 1304, 1153, 826, 682 cm^{-1}. **^{1}H-NMR** (400 MHz, $CDCl_3$): δ 7.74 (d, *J* = 8.0 Hz, 2H), 7.58 (d, *J* = 8.0 Hz, 2H), 7.49-7.45 (m, 4H), 7.36-7.31 (m, 4H), 5.45 (s, 1H), 5.23 (s, 1H), 4.42 (t, *J* = 5.3 Hz, 1H), 4.02 (d, *J* = 5.3 Hz, 2H), 2.44 (s 3H). **^{13}C-NMR {^{1}H}** (100 MHz, $CDCl_3$): δ 143.6, 142.3, 139.8, 139.3, 137.1, 136.8, 131.9, 129.8, 128.6, 127.3, 127.1, 126.6, 121.8, 115.5, 47.1, 21.5 ppm. **ESI-MS** *m/z* (rel int): (pos) 442.0 ($[M+H]^+$, 100); (neg) 440.0 ($[M-H]^-$, 100). **HRMS** (ESI) m/z: $[M+H]^+$ Calcd for: $C_{22}H_{21}BrNO_2S^+$: 442.0471, found: 442.0475. Absolute difference (ppm): 0.90.

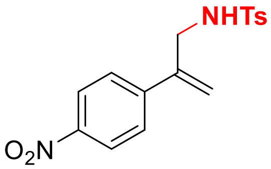

4-methyl-N-(2-(4-nitrophenyl)allyl)benzenesulfonamide (3v): Alkene **2v** (0.1 mmol) reacted under the general method to produce allylic amine **3v** as a pale yellow solid (27 mg, 0.082 mmol, 82%).[4] **TLC**: R_f : 0.46 (2:1 heptanes/EtOAc). **IR** (thin film): υ 3203, 3050, 2964, 1735, 1601, 1519, 1158, 855 cm^{-1}. **^{1}H-NMR** (400 MHz, $CDCl_3$): δ 8.15 (d, *J* = 8.0 Hz, 1H), 7.72 (d, *J* = 8.0 Hz, 1H), 7.45 (d, *J* = 8.0 Hz, 2H), 7.32 (d, *J* = 8.0 Hz, 2H), 5.52 (s, 1H), 5.43 (s, 1H), 4.57 (t, *J* = 5.3 Hz, 1H), 4.04 (d, *J* = 5.3 Hz, 2H), 2.44 (s 3H). **^{13}C-NMR {^{1}H}** (100 MHz, $CDCl_3$): δ 144.3, 143.9, 141.5, 136.6, 129.8, 127.2, 127.1, 126.9, 123.8, 118.1, 46.9, 21.5 ppm. **ESI-MS** *m/z* (rel int): (pos) 333.1 ($[M+H]^+$, 100); (neg) 331.1 ($[M-H]^-$, 100). **HRMS** (ESI) m/z: $[M+H]^+$ Calcd for: $C_{16}H_{17}N_2O_4S^+$: 333.0904, found: 333.0905. Absolute difference (ppm): 0.30.

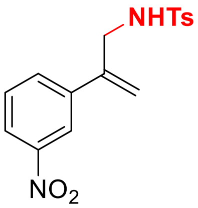

4-methyl-N-(2-(3-nitrophenyl)allyl)benzenesulfonamide (3w): Alkene **2w** (0.1 mmol) reacted under the general method to produce allylic amine **3w** as a light yellow solid (28 mg, 0.084 mmol,

84%).[5] **TLC**: R_f : 0.48 (2:1 heptanes/EtOAc). **IR** (thin film): υ 3256, 3058, 2946, 1753, 1601, 1511, 1158, 853 cm^{-1}. **^{1}H-NMR** (400 MHz, $CDCl_3$): δ 8.14 (d, *J* = 8.0 Hz, 1H), 8.04 (s, 1H), 7.71 (d, *J* = 8.0 Hz, 2H), 7.65 (d, *J* = 7.1 Hz, 1H), 7.49 (t, *J* = 7.1 Hz, 1H), 7.27 (d, *J* = 8.0 Hz, 2H), 5.52 (s, 1H), 5.41 (s, 1H), 4.46 (t, *J* = 5.3 Hz, 1H), 4.06 (d, *J* = 5.3 Hz, 2H), 2.44 (s 3H). **^{13}C-NMR {^{1}H}** (100 MHz, $CDCl_3$): δ 148.3, 143.9, 141.2, 139.7, 136.6, 132.2, 130.5, 129.8, 127.2, 122.9, 121.1, 118.1, 46.9, 21.5 ppm. **ESI-MS** *m/z* (rel int): (pos) 333.1 ($[M+H]^+$, 100); (neg) 331.1 ($[M–H]^–$, 100). **HRMS** (ESI) m/z: $[M+H]^+$ Calcd for: $C_{16}H_{17}N_2O_4S^+$: 333.0904, found: 333.0906. Absolute difference (ppm): 0.60.

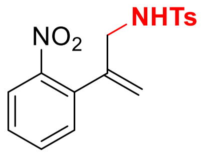

4-methyl-N-(2-(2-nitrophenyl)allyl)benzenesulfonamide (3x): Alkene **2x** (0.1 mmol) reacted under the general method to produce allylic amine **3x** as a light yellow solid (25 mg, 0.075 mmol, 75%). **TLC**: R_f : 0.46 (2:1 heptanes/EtOAc). **IR** (thin film): υ 3212, 3041, 2988, 1735, 1654, 1519, 1133, 835 cm^{-1}. **^{1}H-NMR** (400 MHz, $CDCl_3$): δ 7.81-7.78 (m, 3H), 7.55-7.53 (m, 2H), 7.38 (d, *J* = 8.0 Hz, 2H), 7.32 (dt, *J* = 7.3, 7.1 Hz, 1H), 5.48 (s, 1H), 5.38 (s, 1H), 4.40 (t, *J* = 5.3 Hz, 1H), 4.02 (d, *J* = 5.3 Hz, 2H), 2.44 (s 3H). **^{13}C-NMR {^{1}H}** (100 MHz, $CDCl_3$): δ 147.3, 146.5, 141.7, 137.7, 135.6, 132.2, 129.7, 129.1, 128.5, 127.2, 121.1, 118.2, 46.6, 21.5 ppm. **ESI-MS** *m/z* (rel int): (pos) 333.1 ($[M+H]^+$, 100); (neg) 331.1 ($[M–H]^–$, 100). **HRMS** (ESI) m/z: $[M+H]^+$ Calcd for: $C_{16}H_{17}N_2O_4S^+$: 333.0904, found: 333.0909. Absolute difference (ppm): 1.50.

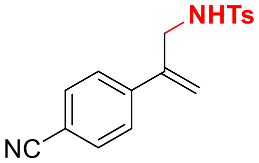

N-(2-(4-cyanophenyl)allyl)-4-methylbenzenesulfonamide (3y): Alkene **2y** (0.1 mmol) reacted under the general method to produce allylic amine **3y** as a white solid (26 mg, 0.091 mmol, 82%).[5] **TLC**: R_f : 0.50 (2:1 heptanes/EtOAc). **IR** (thin film): υ 3265, 3005, 2980, 2240, 1735, 1609, 1159, 853 cm^{-1}. **^{1}H-NMR** (400 MHz, $CDCl_3$): δ 7.72 (d, *J* = 8.0 Hz, 2H), 7.58 (d, *J* = 8.0 Hz, 2H), 7.38 (d, *J* = 8.0 Hz, 2H), 7.30 (d, *J* = 8.0 Hz, 2H), 5.51 (s, 1H), 5.38 (s, 1H), 4.38 (t, *J* = 6.0 Hz, 1H), 4.00 (d, *J* = 6.0 Hz, 2H), 2.46 (s 3H). **^{13}C-NMR {^{1}H}** (100 MHz, $CDCl_3$): δ 143.8, 142.3, 141.7, 136.6, 132.2, 129.8, 127.2, 126.9, 118.6, 111.8, 46.8, 21.6 ppm. **ESI-MS** *m/z* (rel int): (pos) 313.1 ($[M+H]^+$, 100); (neg) 311.1 ($[M–H]^–$, 100). **HRMS** (ESI) m/z: $[M+H]^+$ Calcd for: $C_{17}H_{17}N_2O_2S^+$: 313.1005, found: 313.1010. Absolute difference (ppm): 1.60.

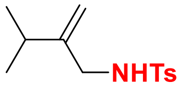

4-methyl-N-(3-methyl-2-methylenebutyl)benzenesulfonamide (3z): Alkene **2z** (0.1 mmol) reacted under the general method to produce allylic amine **3z** as a clear oil (23 mg, 0.091 mmol,

91%). **TLC**: R_f : 0.53 (2:1 heptanes/EtOAc). **IR** (thin film): υ 3252, 3058, 2988, 1744, 1610, 1554, 1151, 817 cm^{-1}. **^{1}H-NMR** (400 MHz, $CDCl_3$): δ 7.72 (d, *J* = 8.0 Hz, 2H), 7.27 (d, *J* = 8.0 Hz, 2H), 4.82 (s, 1H), 4.71 (s, 1H), 4.33-4.31 (m, 1H), 3.45 (d, *J* = 6.2 Hz, 2H), 2.42 (s, 3H), 1.92 (septet, *J* = 7.3 Hz, 1H), 0.88 (d, *J* = 7.3 Hz, 6H). **^{13}C-NMR {^{1}H}** (100 MHz, $CDCl_3$): δ 144.3, 143.4, 136.8, 129.6, 127.2, 112.4, 47.9, 35.8, 20.6, 18.1 ppm. **ESI-MS** *m/z* (rel int): (pos) 254.1 ([M+H]$^+$, 100); (neg) 252.1 ([M–H]$^-$, 100). **HRMS** (ESI) m/z: [M+H]$^+$ Calcd for: $C_{13}H_{20}NO_2S^+$: 254.1209, found: 254.1204. Absolute difference (ppm): 1.97.

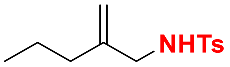

4-methyl-N-(2-methylenepentyl)benzenesulfonamide (3aa): Alkene **2aa** (0.1 mmol) reacted under the general method to produce allylic amine **3aa** as a clear oil (22 mg, 0.089 mmol, 89%). **TLC**: R_f : 0.51 (2:1 heptanes/EtOAc). **IR** (thin film): υ 3221, 3005, 2980, 1735, 1654, 1520, 1133, 871 cm^{-1}. **^{1}H-NMR** (400 MHz, $CDCl_3$): δ 7.72 (d, *J* = 8.0 Hz, 2H), 7.28 (d, *J* = 8.0 Hz, 2H), 4.85 (s, 1H), 4.80 (s, 1H), 4.32-4.30 (m, 1H), 3.46 (d, *J* = 6.2 Hz, 2H), 2.40 (s, 3H), 1.91 (t, *J* = 7.1 Hz, 2H), 1.35 (tq, *J* = 7.3, 7.1 Hz, 2H), 0.82 (t, *J* = 7.3 Hz, 3H). **^{13}C-NMR {^{1}H}** (100 MHz, $CDCl_3$): δ 144.5, 143.5, 136.9, 129.8, 127.2, 112.0, 47.9, 35.7, 21.5, 20.6, 13.7 ppm. **ESI-MS** *m/z* (rel int): (pos) 254.1 ([M+H]$^+$, 100); (neg) 252.1 ([M–H]$^-$, 100). **HRMS** (ESI) m/z: [M+H]$^+$ Calcd for: $C_{13}H_{20}NO_2S^+$: 254.1209, found: 254.1206. Absolute difference (ppm): 1.18.

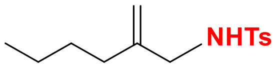

4-methyl-N-(2-methylenehexyl)benzenesulfonamide (3ab): Alkene **2ab** (0.1 mmol) reacted under the general method to produce allylic amine **3ab** as a colorless oil (25 mg, 0.093 mmol, 93%).[7] **TLC**: R_f : 0.53 (2:1 heptanes/EtOAc). **IR** (thin film): υ 3207, 3014, 2944, 1735, 1645, 1511, 1122, 871 cm^{-1}. **^{1}H-NMR** (400 MHz, $CDCl_3$): δ 7.72 (d, *J* = 8.0 Hz, 2H), 7.28 (d, *J* = 8.0 Hz, 2H), 4.84 (s, 1H), 4.79 (s, 1H), 4.33-4.31 (m, 1H), 3.46 (d, *J* = 6.2 Hz, 2H), 2.40 (s, 3H), 1.92 (t, *J* = 7.1 Hz, 2H), 1.28-1.18 (m, 4H), 0.82 (t, *J* = 7.3 Hz, 3H). **^{13}C-NMR {^{1}H}** (100 MHz, $CDCl_3$): δ 144.7, 143.5, 136.9, 129.7, 127.2, 111.9, 47.9, 33.1, 27.1, 22.4, 21.5, 14.0 ppm. **ESI-MS** *m/z* (rel int): (pos) 268.2 ([M+H]$^+$, 100); (neg) 266.2 ([M–H]$^-$, 100). **HRMS** (ESI) m/z: [M+H]$^+$ Calcd for: $C_{14}H_{22}NO_2S^+$: 268.1366, found: 268.1364. Absolute difference (ppm): 0.75.

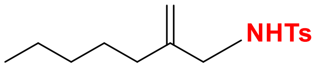

4-methyl-N-(2-methyleneheptyl)benzenesulfonamide (3ac): Alkene **2ac** (0.1 mmol) reacted under the general method to produce allylic amine **3ac** as an oil (25 mg, 0.09 mmol, 90%).[8] **TLC**: R_f : 0.55 (2:1 heptanes/EtOAc). **IR** (thin film): υ 3184, 3005, 2953, 1726, 1645, 1511, 1133, 817 cm^{-1}. **^{1}H-NMR** (400 MHz, $CDCl_3$): δ 7.72 (d, *J* = 8.0 Hz, 2H), 7.28 (d, *J* = 8.0 Hz, 2H), 4.84 (s, 1H), 4.80 (s, 1H), 4.31-4.29 (m, 1H), 3.46 (d, *J* = 6.2 Hz, 2H), 2.40 (s, 3H), 1.89 (t, *J* = 7.1 Hz, 2H), 1.29-1.25 (m, 6H), 0.78 (t, *J* = 7.3 Hz, 3H). **^{13}C-NMR {^{1}H}** (100 MHz, $CDCl_3$): δ 144.5, 143.5, 136.9, 129.7, 127.2, 111.9, 47.9, 38.1, 33.2, 31.2, 22.3, 21.5, 13.9 ppm. **ESI-MS** *m/z* (rel

int): (pos) 282.2 ([M+H]$^+$, 100); (neg) 280.2 ([M−H]$^-$, 100). **HRMS** (ESI) m/z: [M+H]$^+$ Calcd for: $C_{15}H_{24}NO_2S^+$: 282.1522, found: 282.1527. Absolute difference (ppm): 1.77.

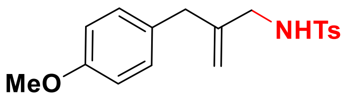

N-(2-(4-methoxybenzyl)allyl)-4-methylbenzenesulfonamide (3ad): Alkene **2ad** (0.1 mmol) reacted under the general method to produce allylic amine **3ad** as a thick oil (29 mg, 0.088 mmol, 88%). **TLC**: R_f : 0.28 (2:1 heptanes/EtOAc). **IR** (thin film): υ 3302, 3058, 2992, 1762, 1636, 1527, 1137, 862 cm^{-1}. **^{1}H-NMR** (400 MHz, $CDCl_3$): δ 7.68 (d, *J* = 8.0 Hz, 2H), 7.24 (d, *J* = 8.0 Hz, 2H), 6.97 (d, *J* = 8.0 Hz, 2H), 6.77 (d, *J* = 8.0 Hz, 2H), 4.94 (d, *J* = 0.8 Hz, 1H), 4.82 (d, *J* = 0.8 Hz, 1H), 4.40 (t, *J* = 6.2 Hz, 1H), 3.76 (s, 3H), 3.42 (dd, *J* = 6.2, 1.6 Hz, 2H), 3.22 (s, 2H), 2.40 (s, 3H). **^{13}C-NMR {^{1}H}** (100 MHz, $CDCl_3$): δ 158.2, 144.3, 143.6, 136.9, 130.3, 129.9, 129.6, 127.2, 127.1, 113.9, 55.2, 47.2, 39.6, 21.5 ppm. **ESI-MS** *m/z* (rel int): (pos) 332.2 ([M+H]$^+$, 100); (neg) 330.2 ([M−H]$^-$, 100). **HRMS** (ESI) m/z: [M+H]$^+$ Calcd for: $C_{18}H_{22}NO_3S^+$: 332.1315, found: 332.1318. Absolute difference (ppm): 0.90.

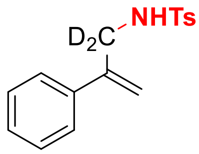

4-methyl-N-(2-phenylallyl-1,1-d2)benzenesulfonamide (3a1-d$_2$): Alkene **2a1-d$_2$** (0.5 mmol) reacted under general procedure for competition experiments with deuterated-alkenes, to produce allylic amine **3a1-d$_2$** as a thick oil (138 mg, 0.48 mmol, 96%). **TLC**: R_f: 0.32 (2:1 heptanes/EtOAc). **MP**: 81-82 °C. **^{1}H-NMR** (400 MHz, $CDCl_3$): δ 7.72 (d, *J* = 8.0 Hz, 2H), 7.32-7.23 (m, 7H), 5.29 (s, 1H), 5.12 (s, 1H), 4.31 (bs, 1H), 2.44 (s, 3H). **^{13}C-NMR {^{1}H}** (100 MHz, $CDCl_3$): δ 144.3, 142.9, 138.4, 136.7, 129.8, 128.6, 128.2, 127.6, 126.3, 116.1, 50.3-50.7 (m), 21.5 ppm. **ESI-MS** *m/z* (rel int): (pos) 290.1 ([M+H]$^+$, 100); (neg) 288.1 ([M−H]$^-$, 100). **HRMS** (ESI) m/z: [M+H]$^+$ Calcd for: $C_{16}H_{16}D_2NO_2S^+$: 290.1178, found: 290.1174. Absolute difference (ppm): 1.79.

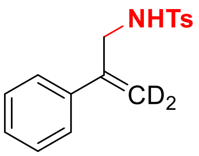

4-methyl-N-(2-phenylallyl-3,3-d2)benzenesulfonamide (3a2-d$_2$): Alkene **2a2-d$_2$** (0.5 mmol) reacted under general procedure for competition experiments with deuterated-alkenes, to produce allylic amine **3a2-d$_2$** as a thick oil (135 mg, 0.48 mmol, 94%). **TLC**: R_f: 0.32 (2:1 heptanes/EtOAc). **MP**: 80-82 °C. **^{1}H-NMR** (400 MHz, $CDCl_3$): δ 7.74 (d, *J* = 8.0 Hz, 2H), 7.32-7.21 (m, 7H), 4.62 (t, *J* = 6.2 Hz, 1H), 4.02 (d, *J* = 6.2 Hz, 2H), 2.44 (s, 3H). **^{13}C-NMR {^{1}H}** (100 MHz, $CDCl_3$): δ 144.5, 142.9, 137.9, 136.7, 129.8, 128.6, 128.2, 127.9, 126.4, 115.1-115.9 (m), 51.2, 21.5 ppm. **ESI-MS** *m/z* (rel int): (pos) 290.1 ([M+H]$^+$, 100); (neg) 288.1 ([M−H]$^-$, 100).

HRMS (ESI) m/z: $[M+H]^+$ Calcd for: $C_{16}H_{16}D_2NO_2S^+$: 290.1178, found: 290.1173. Absolute difference (ppm): 1.72.

References:

1. Souto, J. A.; Zian, D.; Muniz, K. Iodine(III)-Mediated Intermolecular Allylic Amination under Metal-Free Conditions, *J. Am. Chem. Soc.* **2012**, 134, 7242.

2. Liu, Y.; Che, C.-M. $[Fe^{III}(F_{20}\text{-tpp})Cl]$ Is an Effective Catalyst for Nitrene Transfer Reactions and Amination of Saturated Hydrocarbons with Sulfonyl and Aryl Azides as Nitrogen Source under Thermal and Microwave-Assisted Conditions, *Chem. A Eur. J.* **2010**, 16, 10494.

3. Chang, M.-Y.; Chen, Y.-C.; Chan, C.-K. N-Bromosuccinamide-mediated reaction of cyclic styrenes with chloramine-T. *Tet. Lett.* **2014**, 55, 4767.

4. Cabre, A.; Sciortino, G.; Ujaque, G.; Verdaguer, X.; Lledos, A.; Riera, A. Iridium-Catalyzed Isomerization of N-Sulfonyl Aziridines to Allyl Amines, *Org. Lett.* **2018**, 20, 5747.

5. Dong, X.; Xu, L.-P.; Yang, Y.; Liu, Y.; Li, X.; Liu, Q.; Zheng, L.; Wang, F.; Liu, H. A palladium/Et_3N·HI-catalyzed highly selective 7-*endo* alkyl-Heck-type reaction of epoxides and a DFT study on the mechanism, *Org. Chem. Front.* **2021**, 8, 6009.

6. Kiyokawa, K.; Kojima, T.; Hishikawa, Y.; Minakata, S. Iodine-Catalyzed Decarboxylative Amidation of β,γ-Unsaturated Carboxylic Acids with Chloramine Salts Leading to Allylic Amides, *Chem. A Eur. J.* **2015**, 21, 15548.

7. Kim, H.; Lim, W.; Im, D.; Kim, D.-G.; Rhee, Y.; H. Synthetic Strategy for Cyclic Amines: A Stereodefined Cyclic N,O-Acetal as a Stereocontrol and Diversity-Generating Element. *Agew. Chem. Int. Ed.* **2012**, 51, 12055.

8. Blaszykowski, C.; Dhimane, A.-L.; Fensterbank, L.; Malacria, M. N-Silyl-Tethered Radical Cyclizations: A New Synthesis of **γ**-Amino Alcohols. *Org. Lett.* **2003**, 5, 1341.

E. ^{1}H-NMR AND ^{13}C-NMR SPECTRA

pdata/1
KD-14-3

CDCl3, 400 MHz

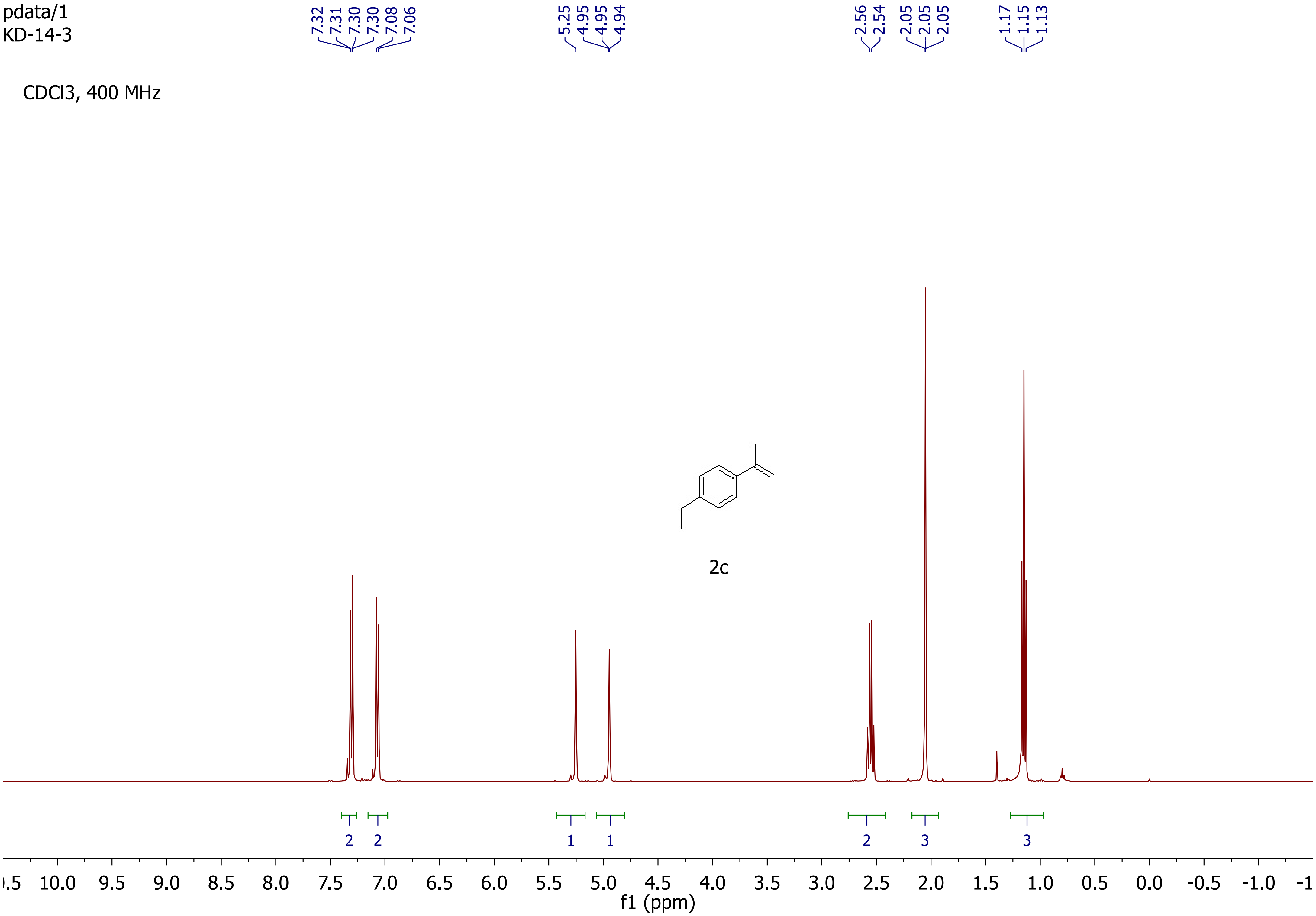

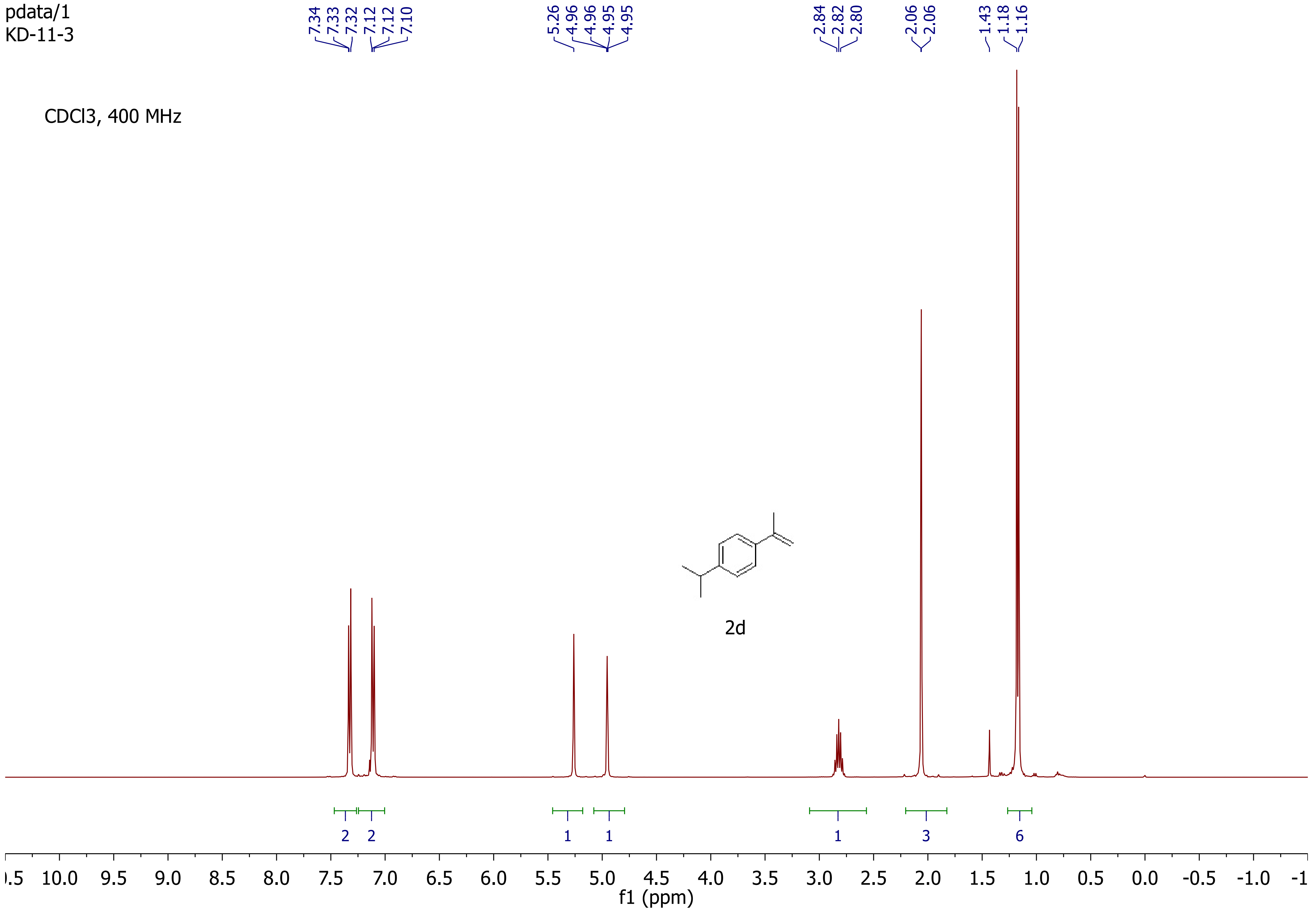
pdata/1
KD-11-3
CDCl3, 400 MHz
7.34 7.33 7.32 7.12 7.12 7.10
5.26 4.96 4.96 4.95 4.95
2.84 2.82 2.80
2.06 2.06
1.43 1.18 1.16
2d
2 2 1 1 1 3 6
10.0 9.5 9.0 8.5 8.0 7.5 7.0 6.5 6.0 5.5 5.0 4.5 4.0 3.5 3.0 2.5 2.0 1.5 1.0 0.5 0.0 -0.5 -1.0
f1 (ppm)

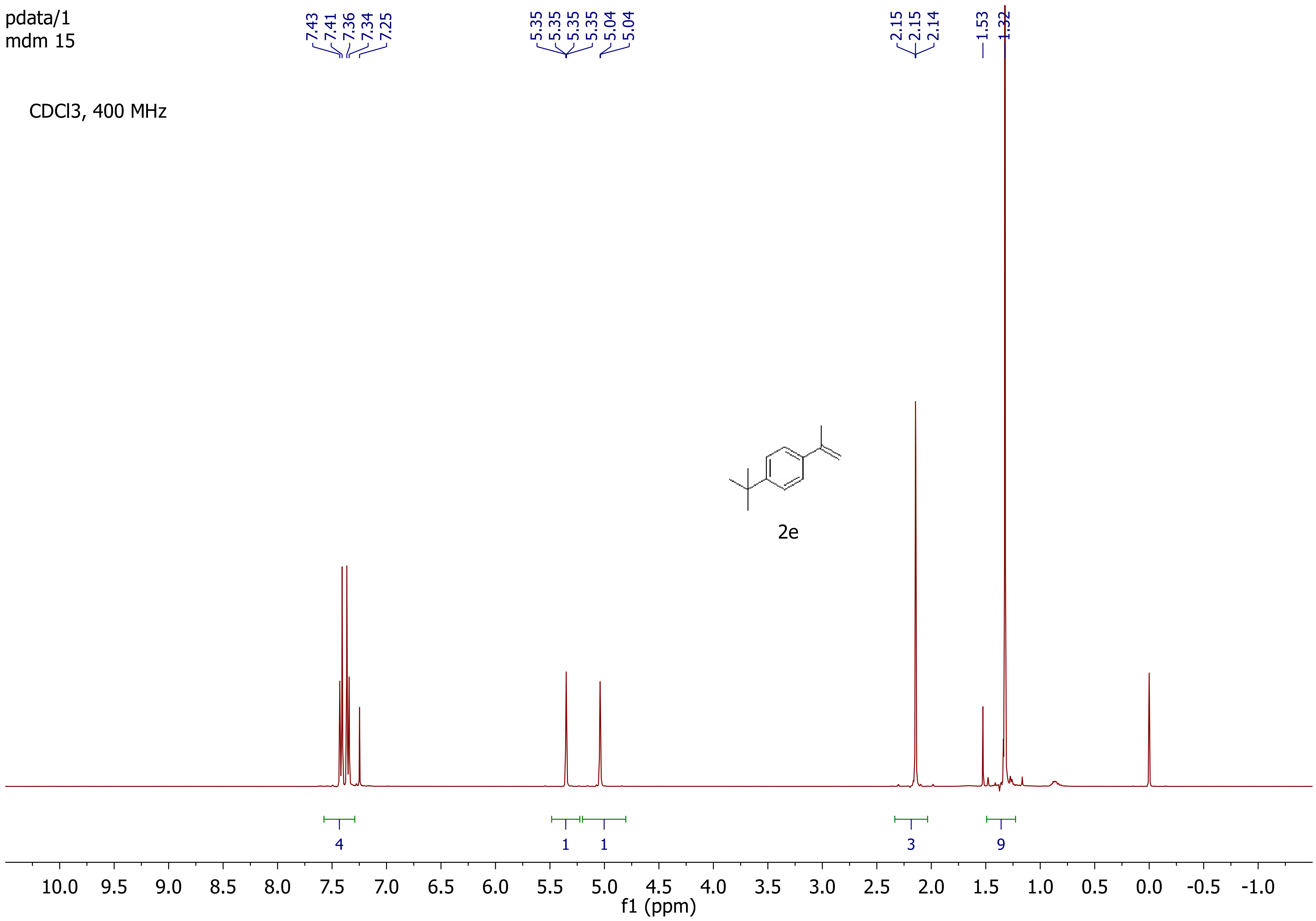
pdata/1
mdm 15
CDCl3, 400 MHz
7.43
7.41
7.36
7.34
7.25
5.35
5.35
5.35
5.35
5.04
5.04
2.15
2.15
2.14
1.53
1.32
2e
4
1
1
3
9
10.0 9.5 9.0 8.5 8.0 7.5 7.0 6.5 6.0 5.5 5.0 4.5 4.0 3.5 3.0 2.5 2.0 1.5 1.0 0.5 0.0 -0.5 -1.0
f1 (ppm)

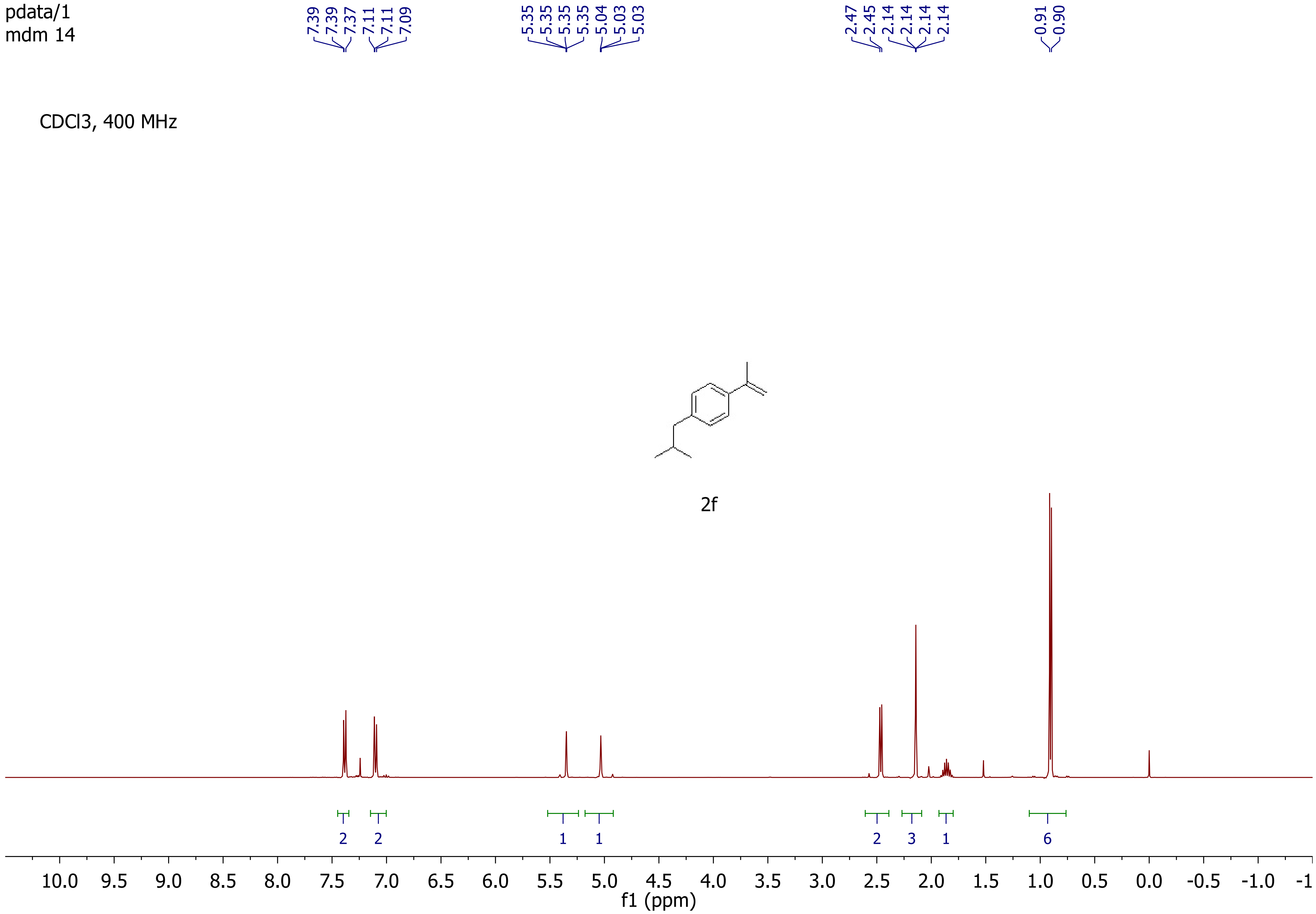
pdata/1
mdm 14
CDCl3, 400 MHz
7.39
7.39
7.37
7.11
7.11
7.09
5.35
5.35
5.35
5.35
5.04
5.03
5.03
2.47
2.45
2.14
2.14
2.14
2.14
0.91
0.90
2f
2
2
1
1
2
3
1
6
10.0 9.5 9.0 8.5 8.0 7.5 7.0 6.5 6.0 5.5 5.0 4.5 4.0 3.5 3.0 2.5 2.0 1.5 1.0 0.5 0.0 -0.5 -1.0 -1
f1 (ppm)

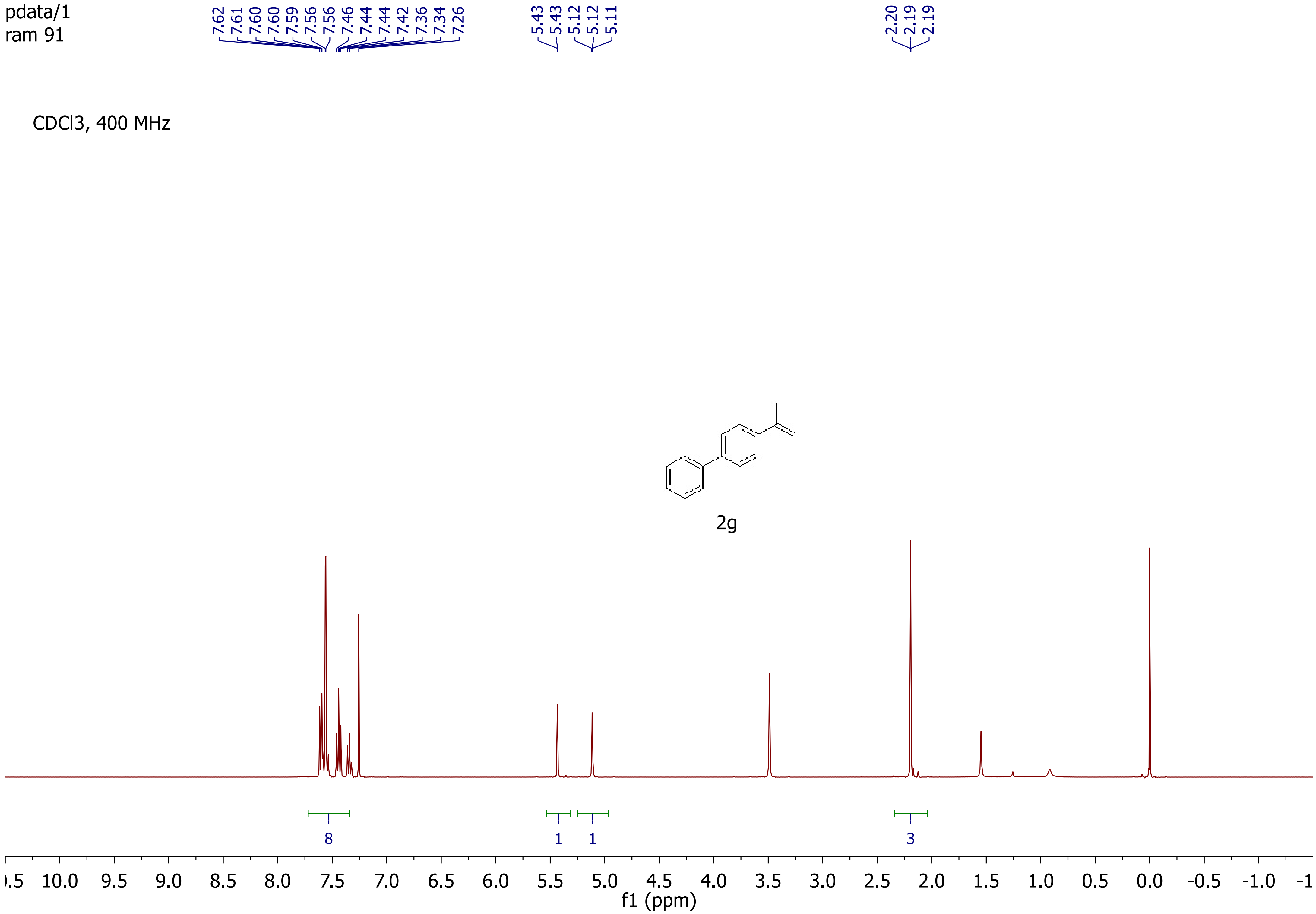
pdata/1
ram 91
7.62
7.61
7.60
7.60
7.59
7.56
7.56
7.46
7.44
7.44
7.42
7.36
7.34
7.26
5.43
5.43
5.12
5.12
5.11
2.20
2.19
2.19
CDCl3, 400 MHz
2g
8
1
1
3
10.0 9.5 9.0 8.5 8.0 7.5 7.0 6.5 6.0 5.5 5.0 4.5 4.0 3.5 3.0 2.5 2.0 1.5 1.0 0.5 0.0 -0.5 -1.0
f1 (ppm)

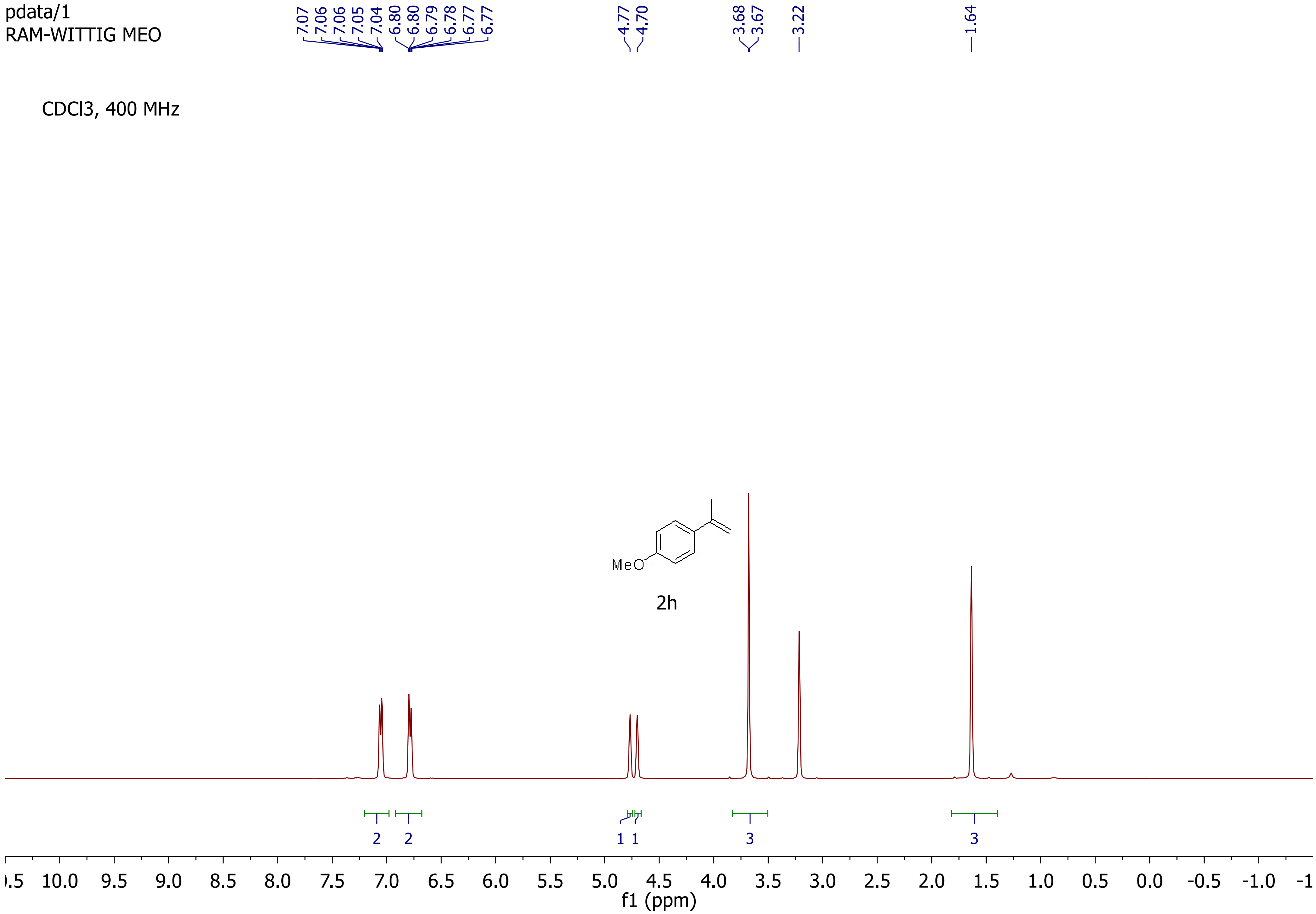
pdata/1
RAM-WITTIG MEO
CDCl3, 400 MHz
7.07 7.06 7.06 7.05 7.04 6.80 6.80 6.79 6.78 6.77 6.77
4.77 4.70
3.68 3.67
3.22
1.64
MeO
2h
2 2 1 1 3 3
.5 10.0 9.5 9.0 8.5 8.0 7.5 7.0 6.5 6.0 5.5 5.0 4.5 4.0 3.5 3.0 2.5 2.0 1.5 1.0 0.5 0.0 -0.5 -1.0 -1
f1 (ppm)

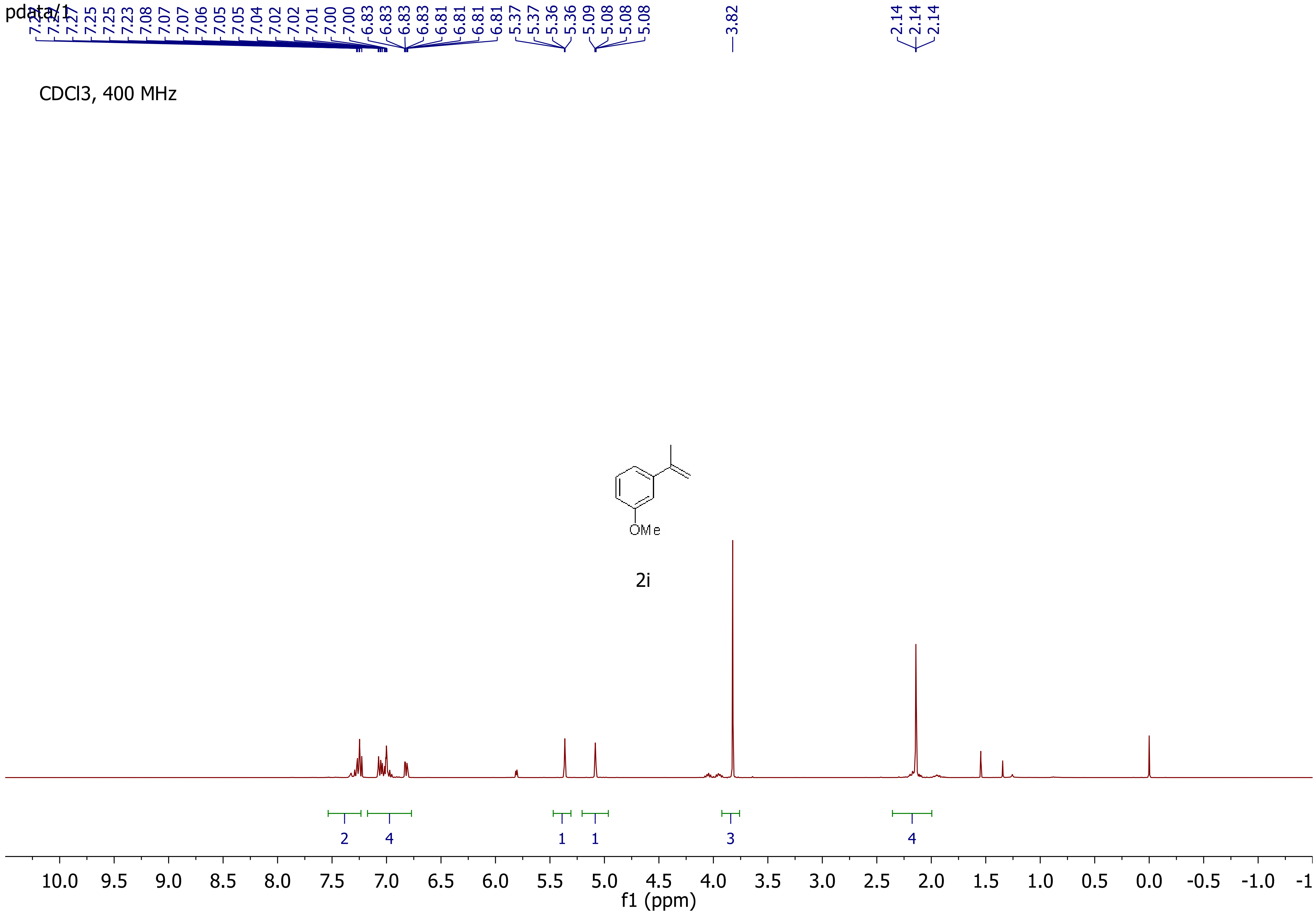
pdata/1
7.27 7.25 7.25 7.23 7.08 7.07 7.07 7.06 7.05 7.05 7.04 7.02 7.02 7.01 7.00 6.83 6.83 6.83 6.83 6.81 6.81 6.81 6.81 5.37 5.37 5.36 5.36 5.09 5.08 5.08 5.08
3.82
2.14 2.14 2.14
CDCl3, 400 MHz
OMe
2i
2 4 1 1 3 4
10.0 9.5 9.0 8.5 8.0 7.5 7.0 6.5 6.0 5.5 5.0 4.5 4.0 3.5 3.0 2.5 2.0 1.5 1.0 0.5 0.0 -0.5 -1.0 -1
f1 (ppm)

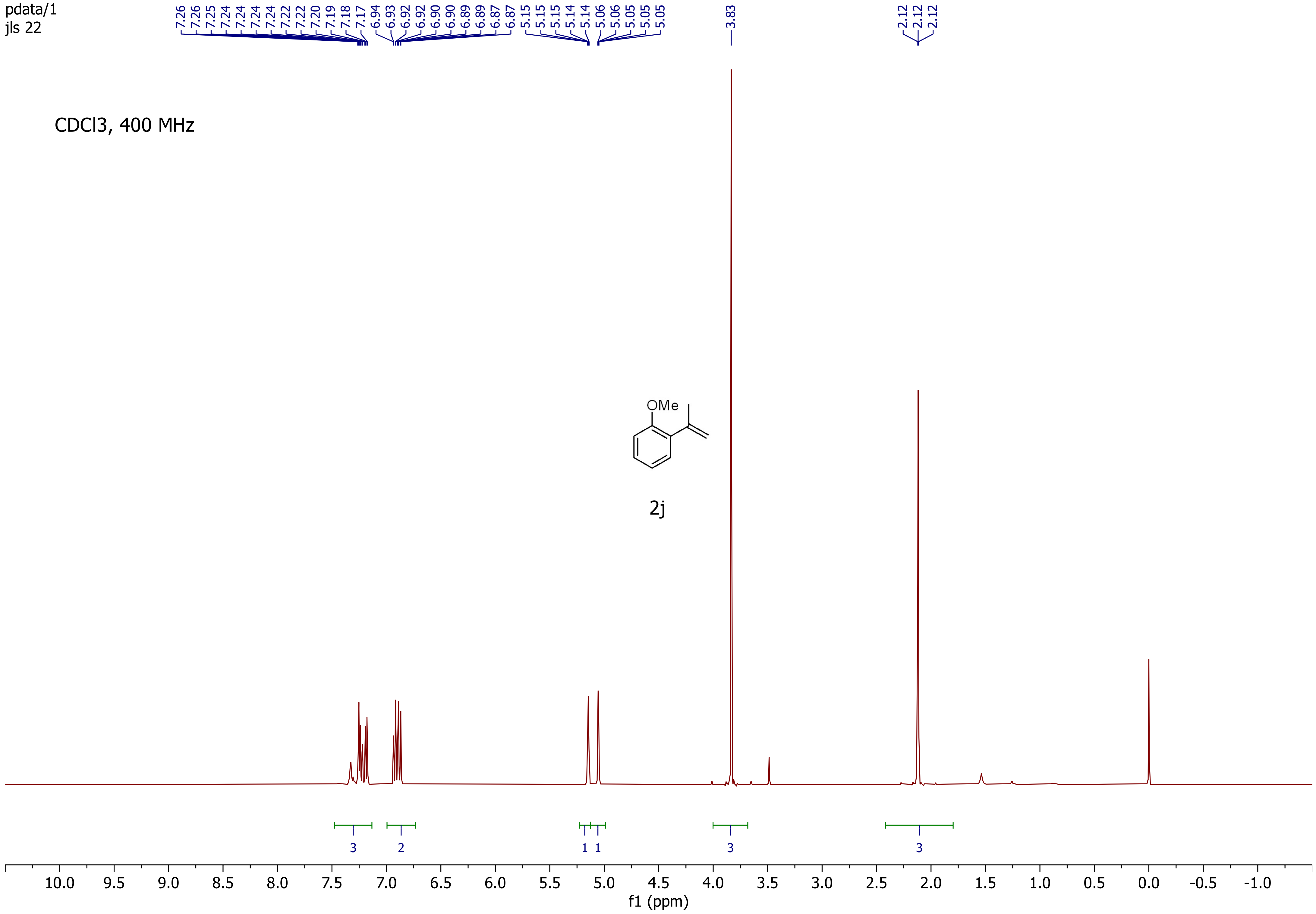
pdata/1
jls 22
7.26 7.26 7.25 7.24 7.24 7.24 7.24 7.22 7.22 7.20 7.19 7.18 7.17 6.94 6.93 6.92 6.92 6.90 6.90 6.89 6.89 6.87 6.87 5.15 5.15 5.15 5.14 5.14 5.06 5.06 5.05 5.05 5.05
3.83
2.12 2.12 2.12
CDCl3, 400 MHz
OMe
2j
3 2 1 1 3 3
10.0 9.5 9.0 8.5 8.0 7.5 7.0 6.5 6.0 5.5 5.0 4.5 4.0 3.5 3.0 2.5 2.0 1.5 1.0 0.5 0.0 -0.5 -1.0
f1 (ppm)

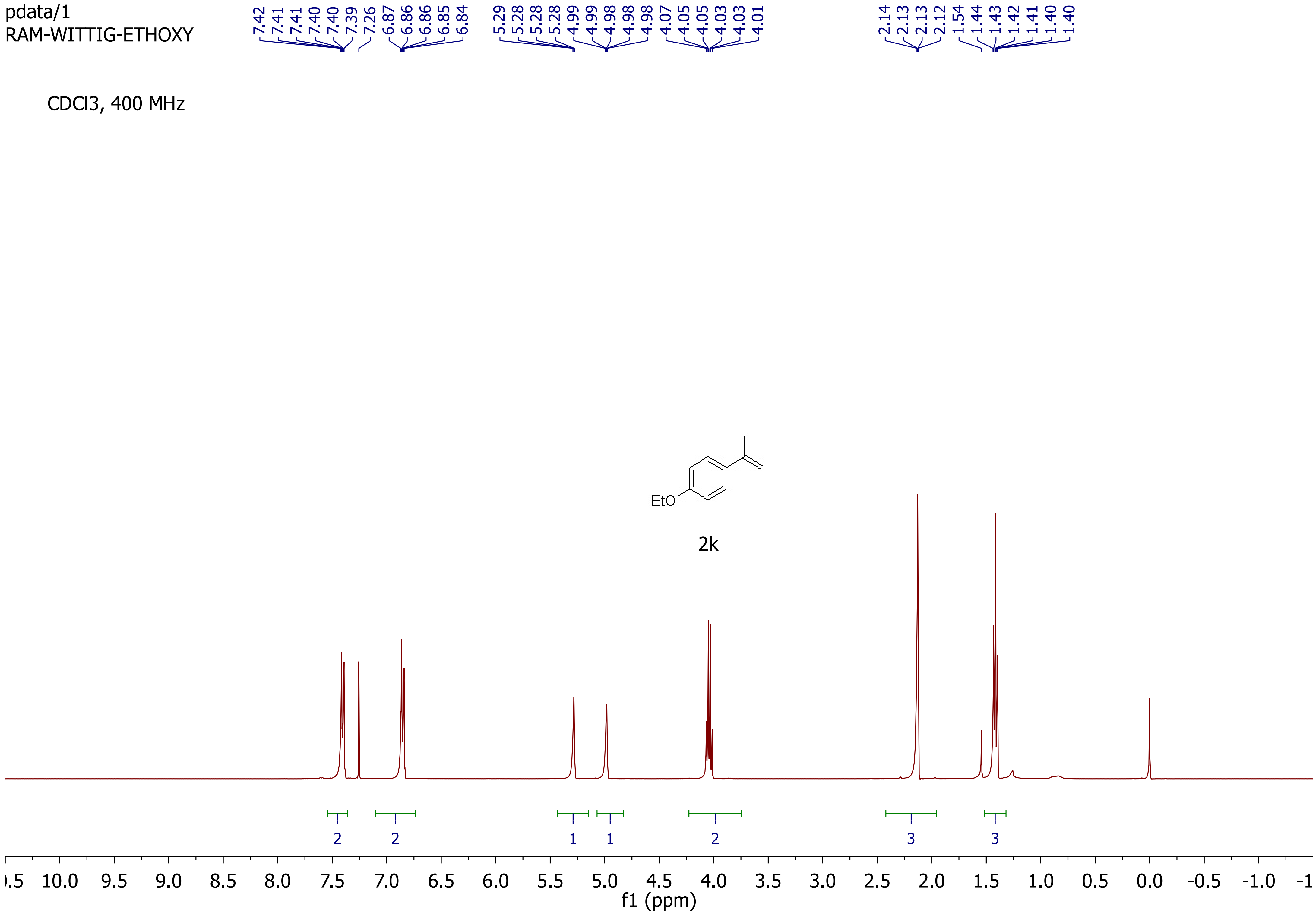

pdata/1
RAM-WITTIG-ETHOXY
CDCl3, 400 MHz
7.42 7.41 7.41 7.40 7.40 7.39 7.26 6.87 6.86 6.86 6.85 6.84 5.29 5.28 5.28 5.28 4.99 4.99 4.98 4.98 4.98 4.07 4.05 4.05 4.03 4.03 4.01 2.14 2.13 2.13 2.12 1.54 1.44 1.43 1.42 1.41 1.40 1.40
EtO
2k
2 2 1 1 2 3 3
f1 (ppm)

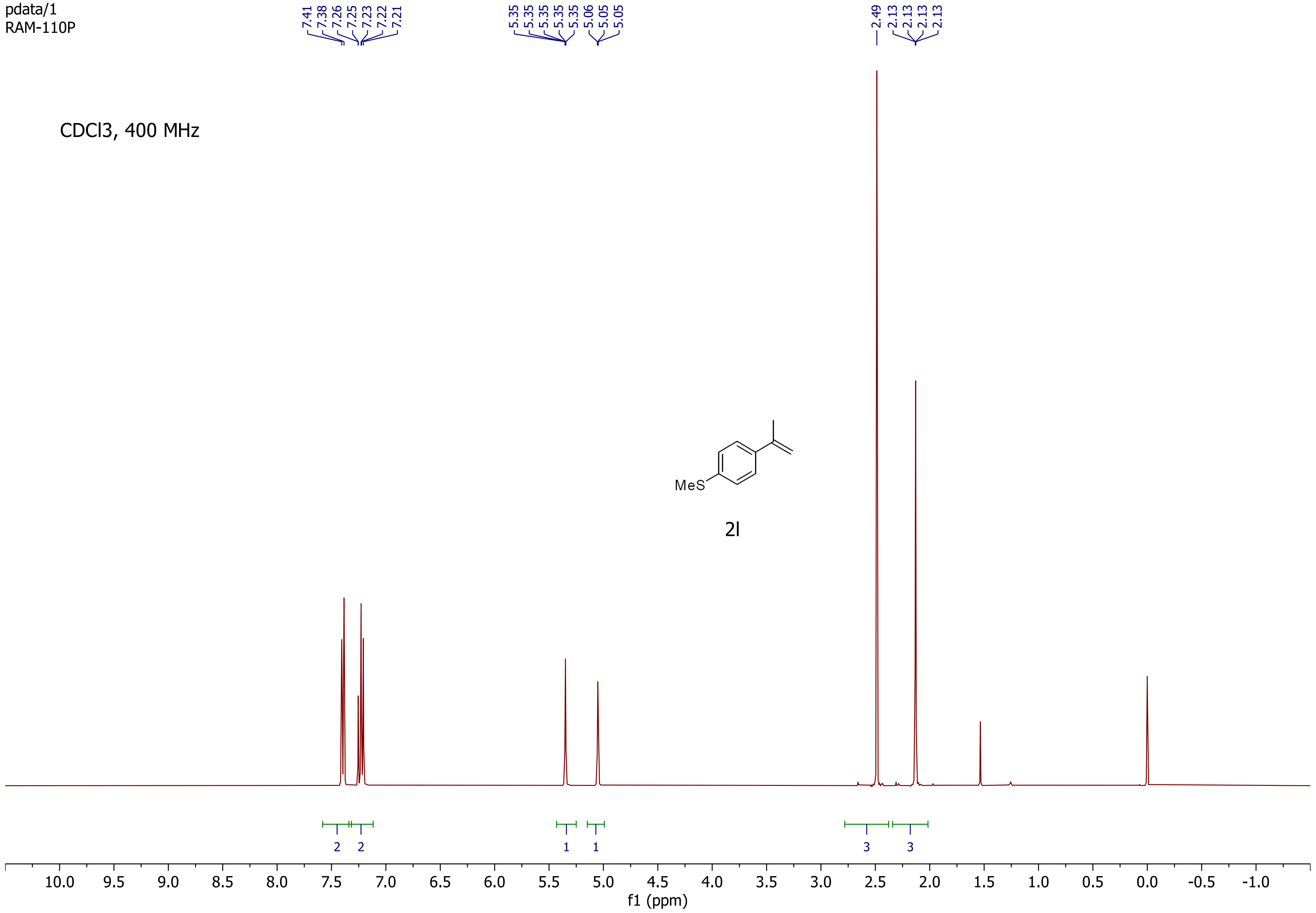

pdata/1
RAM-110P
7.41
7.38
7.26
7.25
7.23
7.22
7.21
5.35
5.35
5.35
5.35
5.35
5.06
5.05
5.05
2.49
2.13
2.13
2.13
2.13
CDCl3, 400 MHz
MeS
2l
2
2
1
1
3
3
10.0
9.5
9.0
8.5
8.0
7.5
7.0
6.5
6.0
5.5
5.0
4.5
4.0
3.5
3.0
2.5
2.0
1.5
1.0
0.5
0.0
-0.5
-1.0
f1 (ppm)

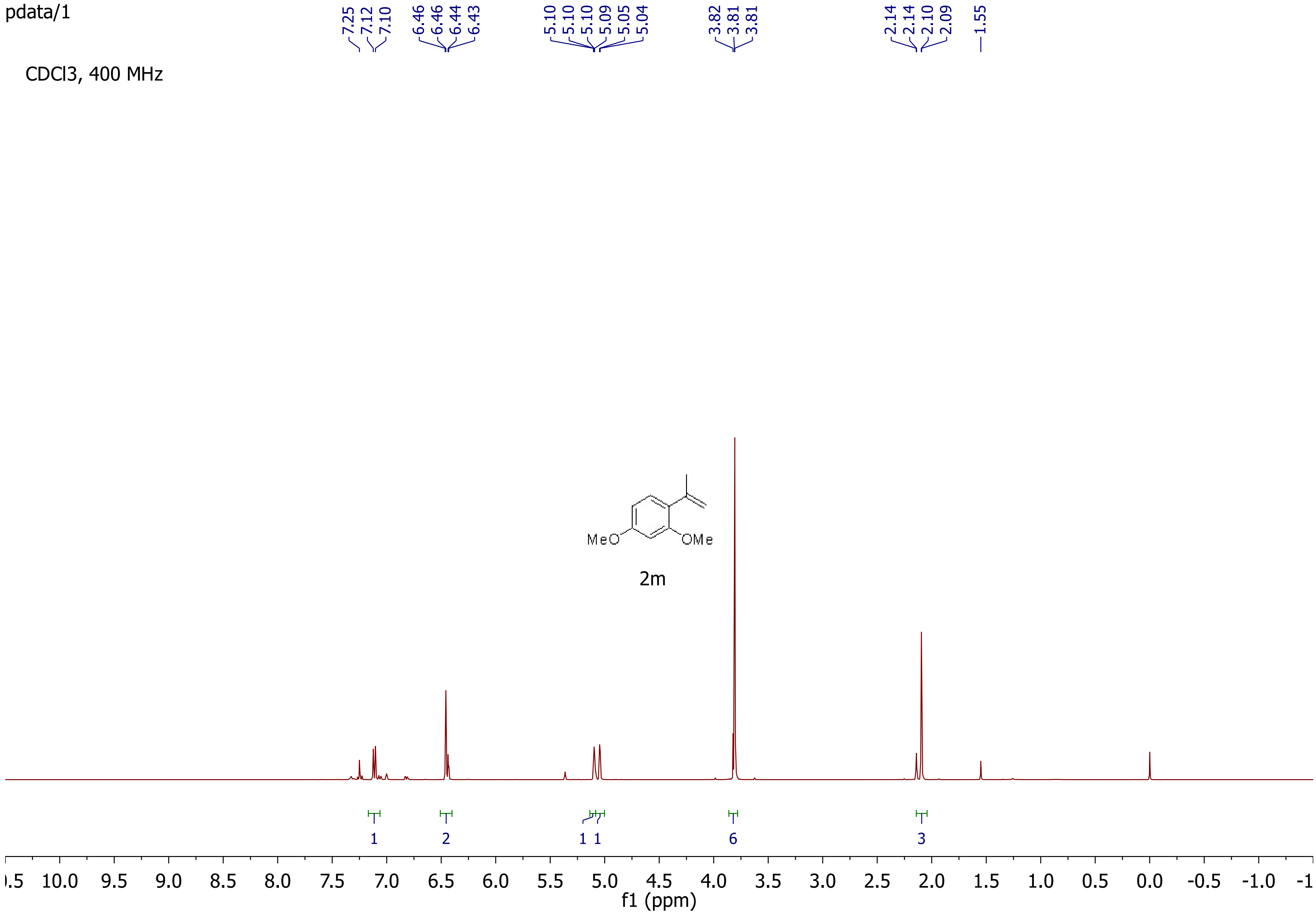
pdata/1
CDCl3, 400 MHz
7.25
7.12
7.10
6.46
6.46
6.44
6.43
5.10
5.10
5.10
5.09
5.05
5.04
3.82
3.81
3.81
2.14
2.14
2.10
2.09
1.55
MeO
OMe
2m
1
2
1
1
6
3
f1 (ppm)

pdata/1
ram 85

CDCl3, 400 MHz

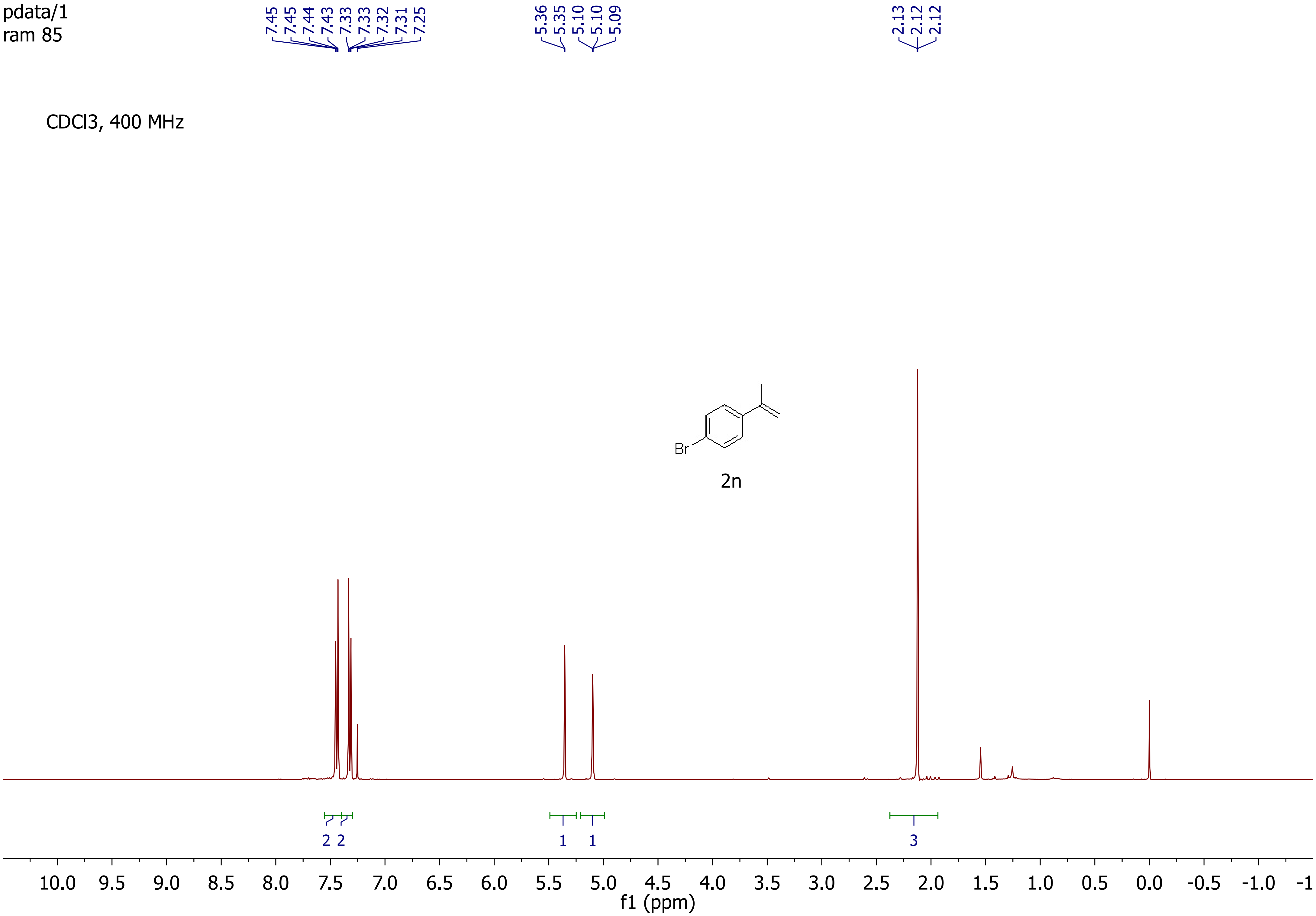

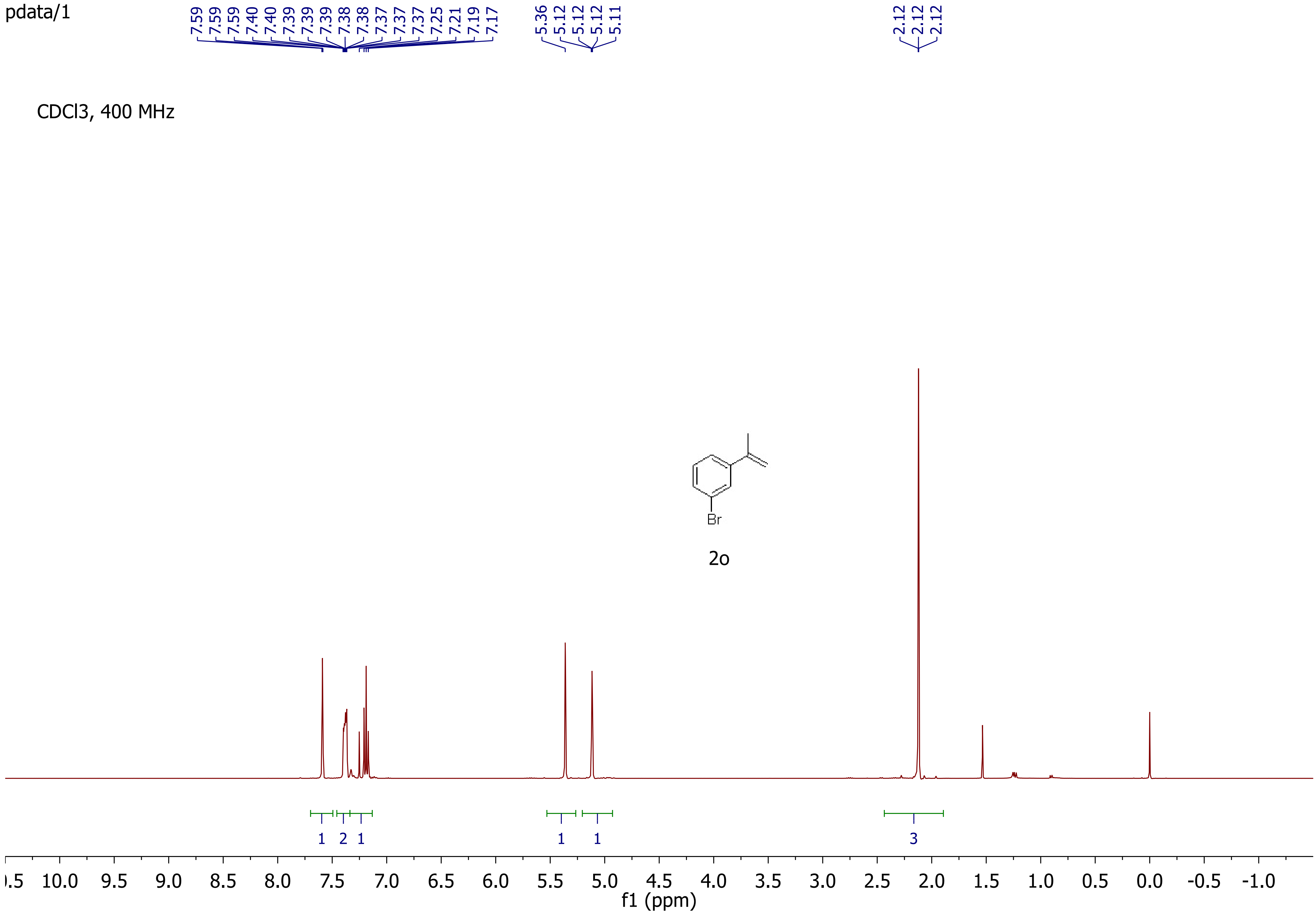
pdata/1
7.59
7.59
7.59
7.40
7.40
7.39
7.39
7.39
7.38
7.38
7.37
7.37
7.37
7.25
7.21
7.19
7.17
5.36
5.12
5.12
5.12
5.11
2.12
2.12
2.12
CDCl3, 400 MHz
Br
2o
1
2
1
1
1
3
10.0
9.5
9.0
8.5
8.0
7.5
7.0
6.5
6.0
5.5
5.0
4.5
4.0
3.5
3.0
2.5
2.0
1.5
1.0
0.5
0.0
-0.5
-1.0
f1 (ppm)

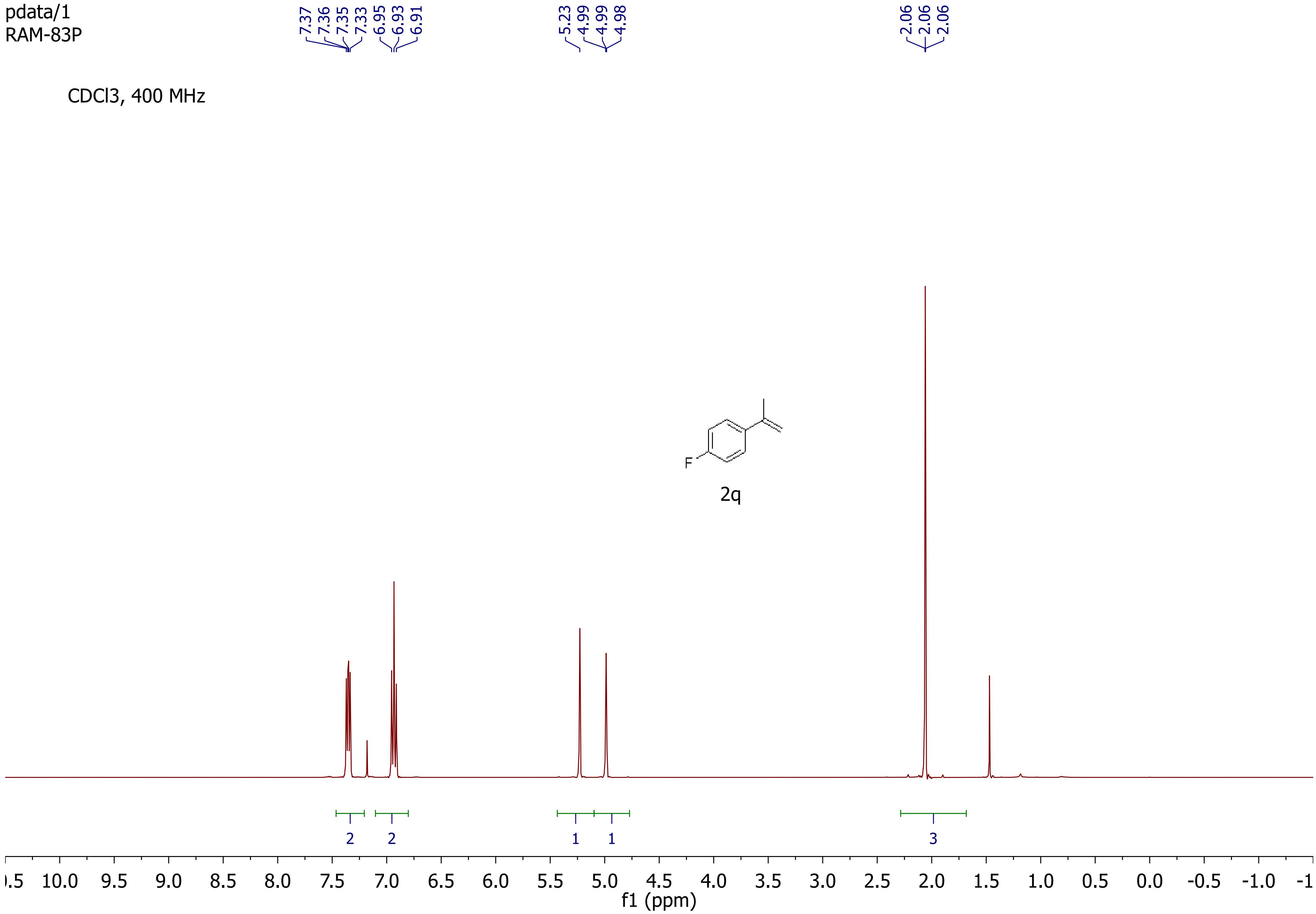
pdata/1
RAM-83P
7.37
7.36
7.35
7.33
6.95
6.93
6.91
5.23
4.99
4.99
4.98
2.06
2.06
2.06
CDCl3, 400 MHz
F
2q
2
2
1
1
3
10.0 9.5 9.0 8.5 8.0 7.5 7.0 6.5 6.0 5.5 5.0 4.5 4.0 3.5 3.0 2.5 2.0 1.5 1.0 0.5 0.0 -0.5 -1.0
f1 (ppm)

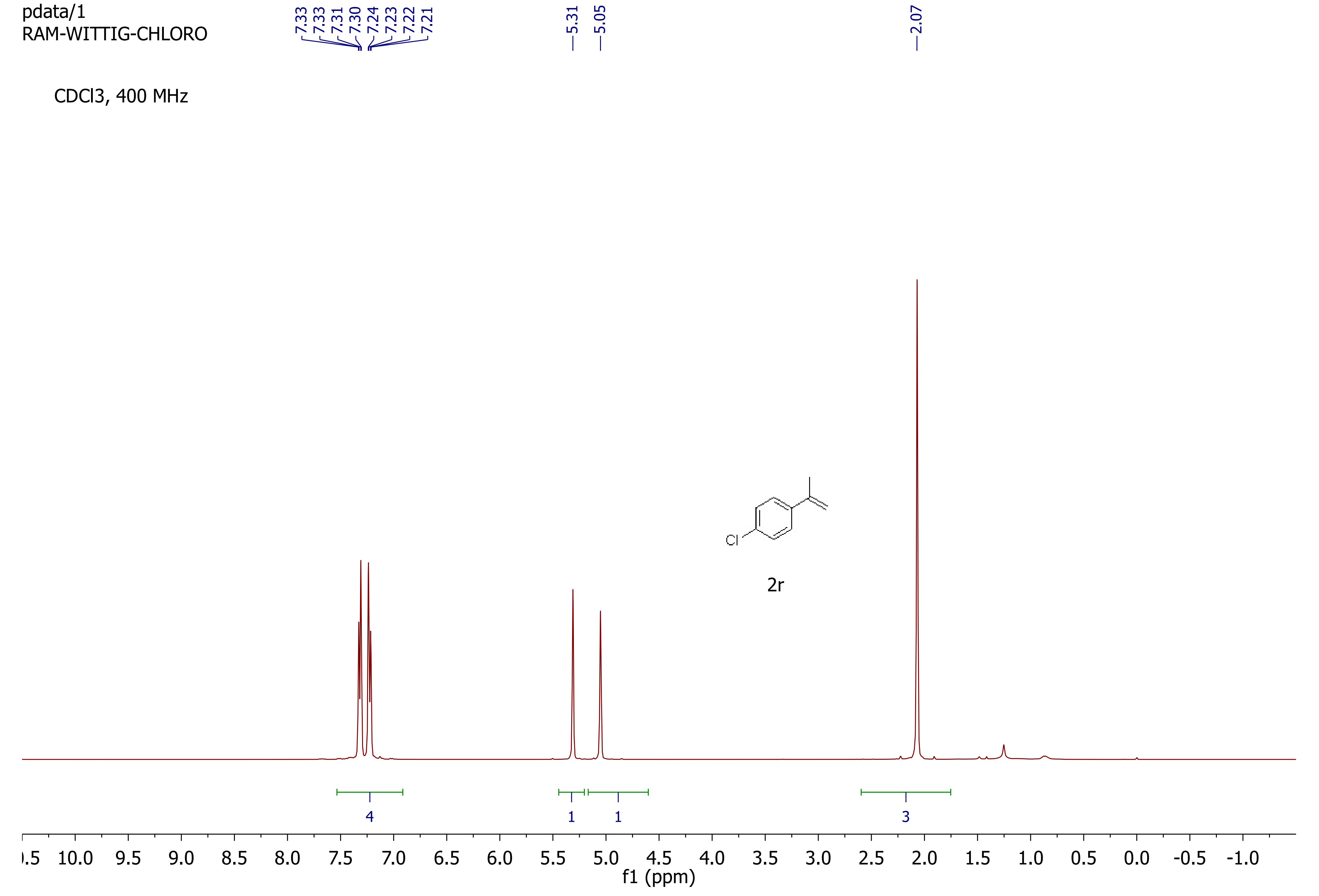
pdata/1
RAM-WITTIG-CHLORO
CDCl3, 400 MHz
7.33
7.33
7.31
7.30
7.24
7.23
7.22
7.21
5.31
5.05
2.07
Cl
2r
4
1
1
3
10.0 9.5 9.0 8.5 8.0 7.5 7.0 6.5 6.0 5.5 5.0 4.5 4.0 3.5 3.0 2.5 2.0 1.5 1.0 0.5 0.0 -0.5 -1.0
f1 (ppm)

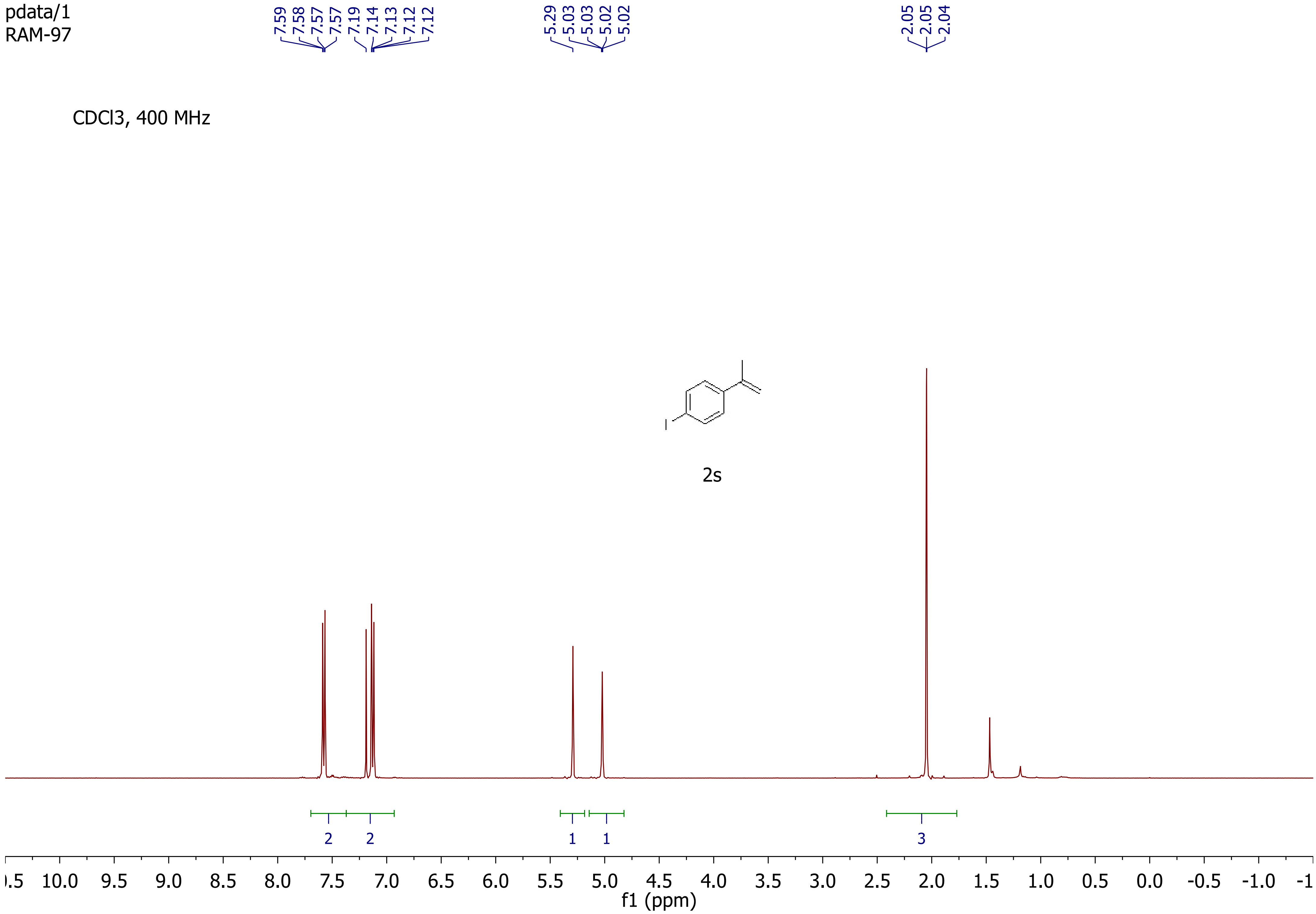
pdata/1
RAM-97
7.59
7.58
7.57
7.57
7.19
7.14
7.13
7.12
7.12
5.29
5.03
5.03
5.02
5.02
2.05
2.05
2.04
CDCl3, 400 MHz
I
2s
2
2
1
1
3
f1 (ppm)

pdata/1

CDCl3, 400 MHz

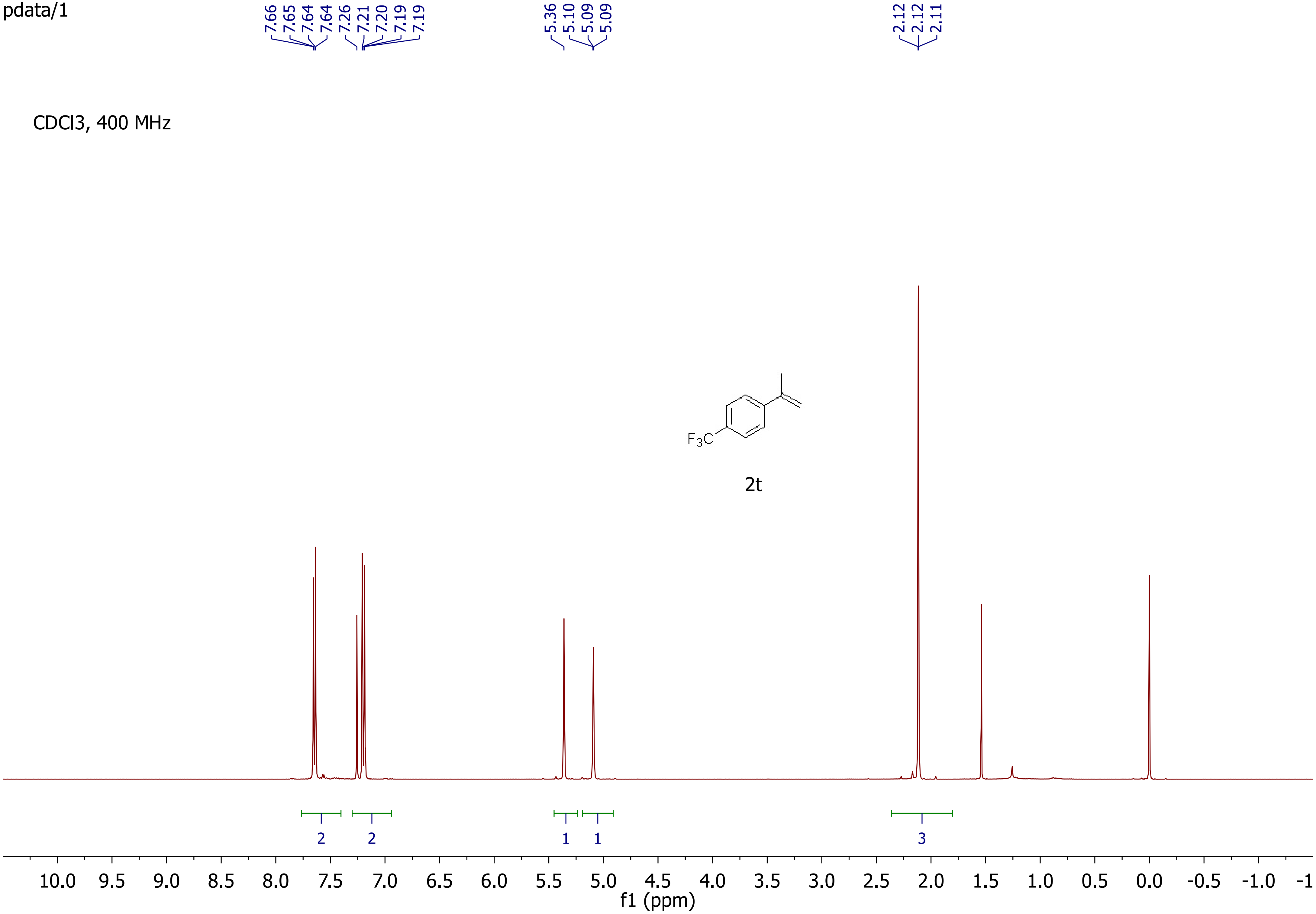

2t

pdata/1
KDꞏ12-3

CDCl3, 400 MHz

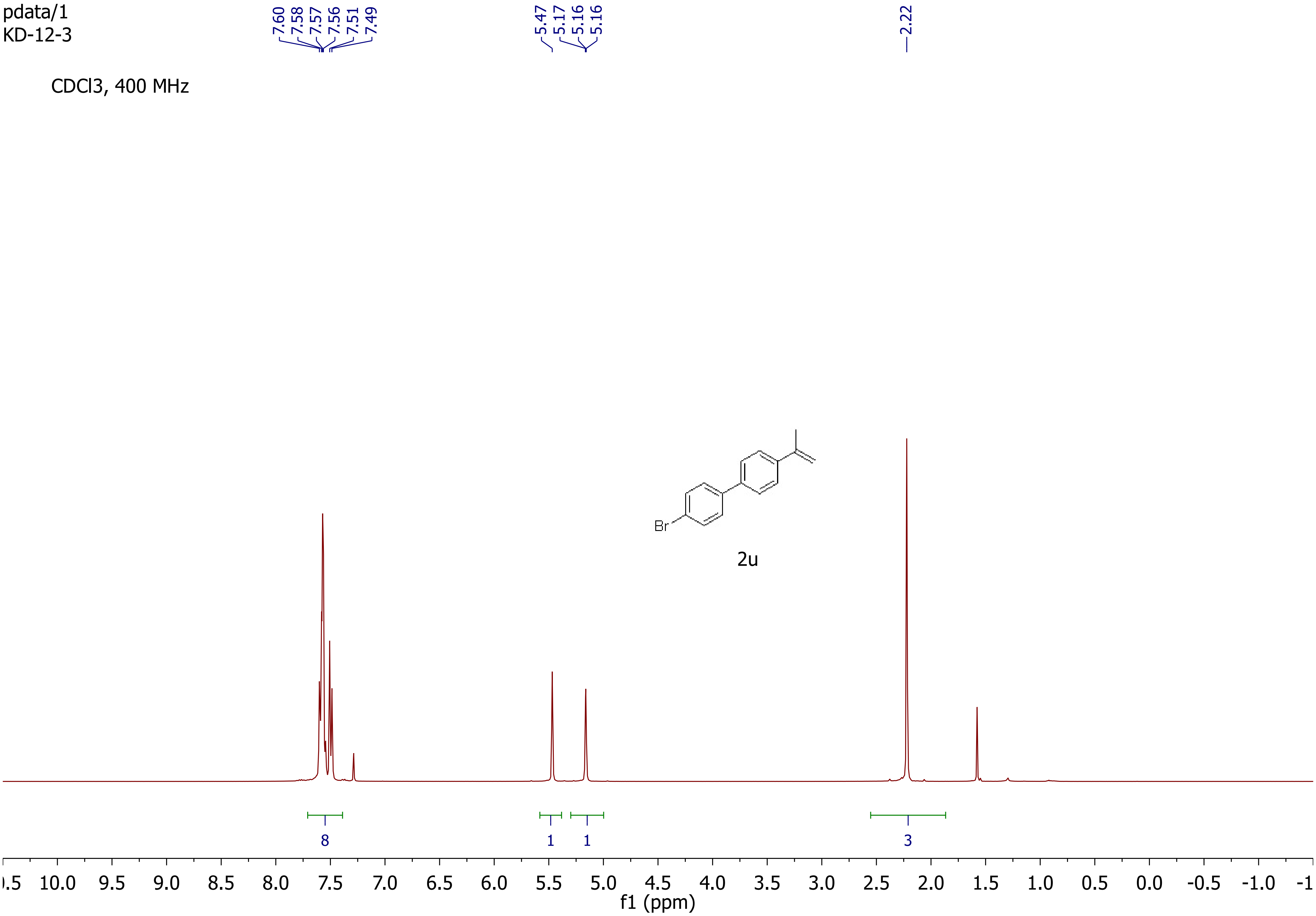

2u

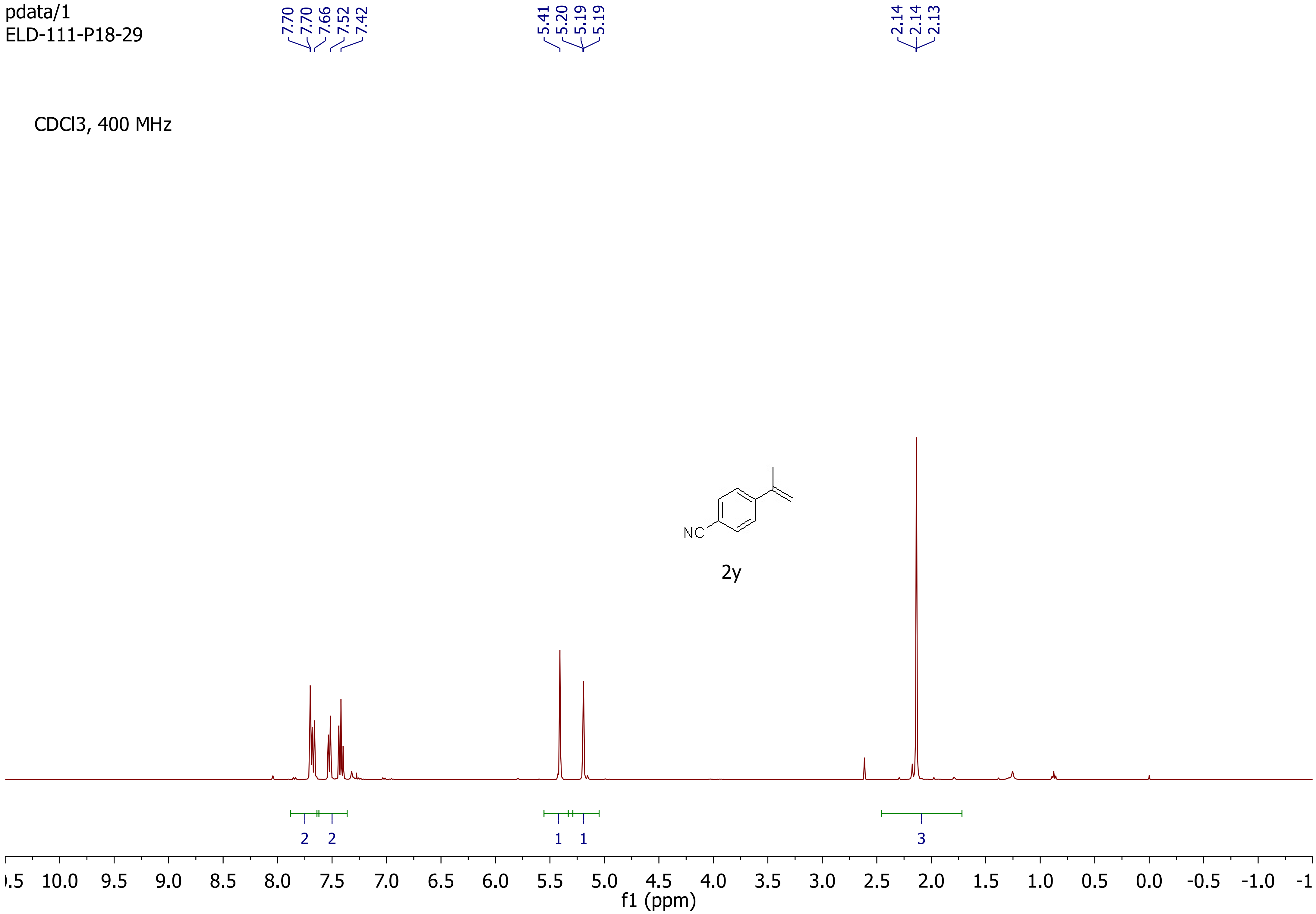
pdata/1
ELD-111-P18-29
CDCl3, 400 MHz
7.70
7.70
7.66
7.52
7.42
5.41
5.20
5.19
5.19
2.14
2.14
2.13
NC
2y
2
2
1
1
3
f1 (ppm)

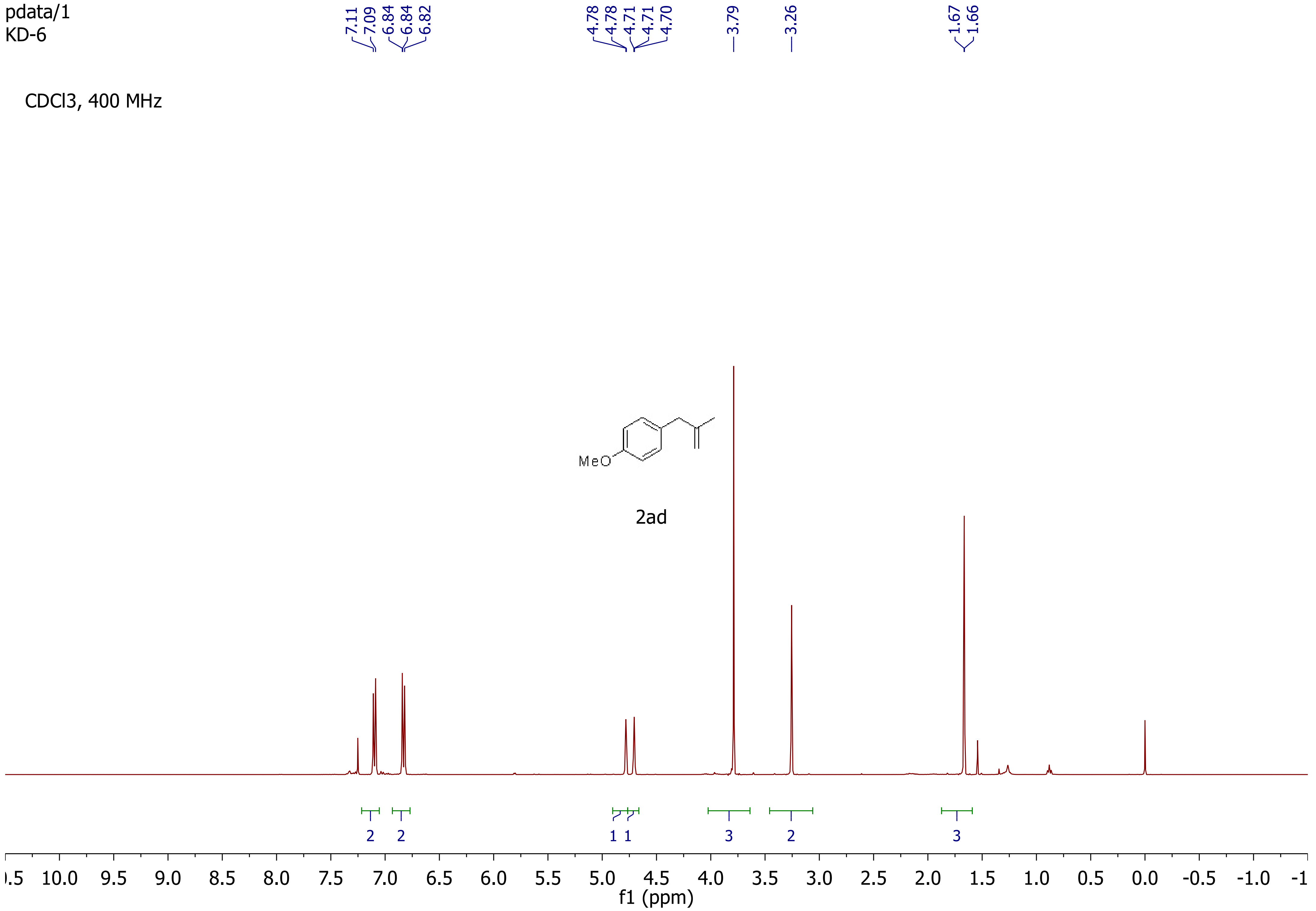
pdata/1
KD-6
CDCl3, 400 MHz
7.11
7.09
6.84
6.84
6.82
4.78
4.78
4.71
4.71
4.70
3.79
3.26
1.67
1.66
MeO
2ad
2
2
1
1
3
2
3
f1 (ppm)

pdata/1

CD3OD, 400 MHz

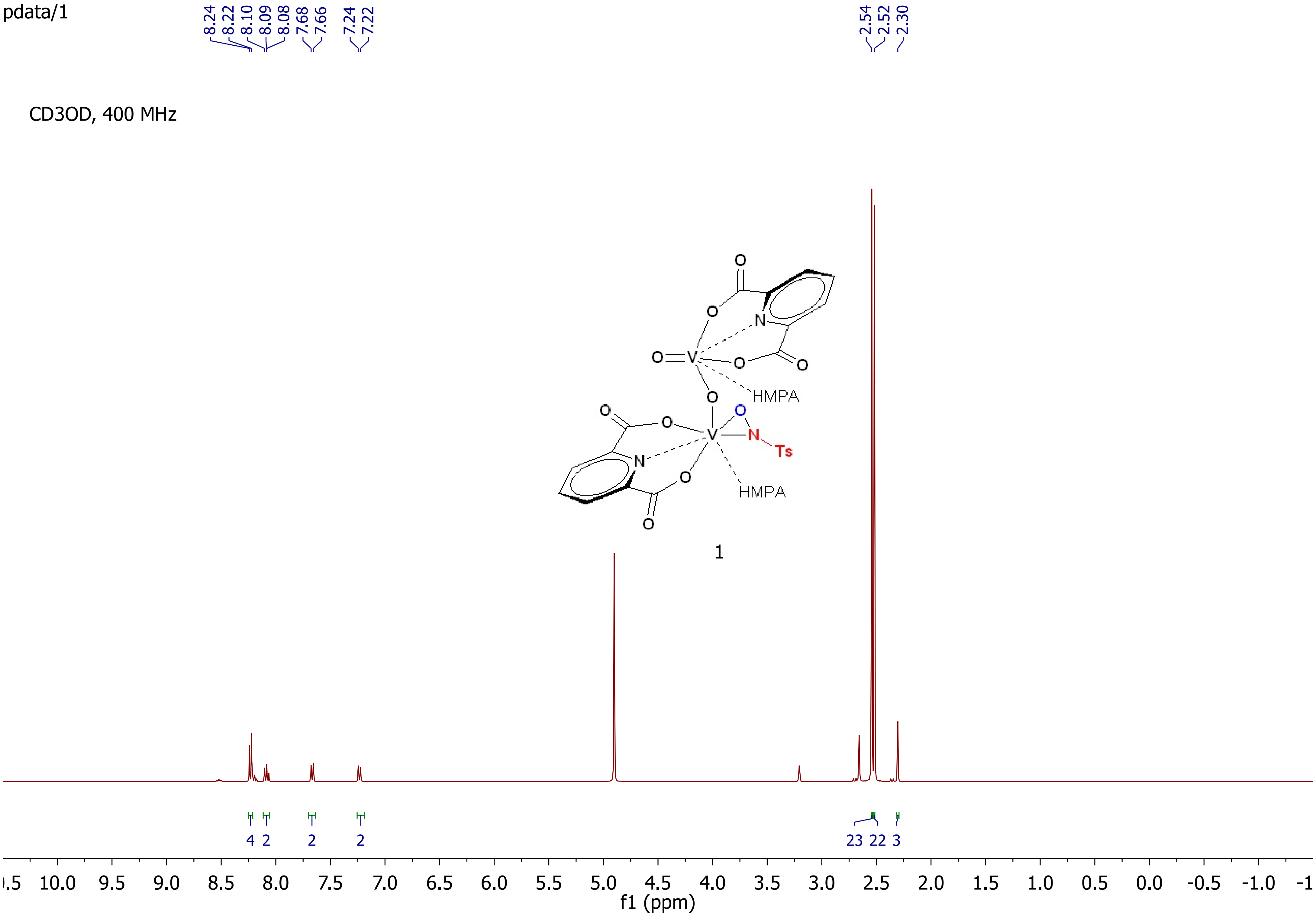

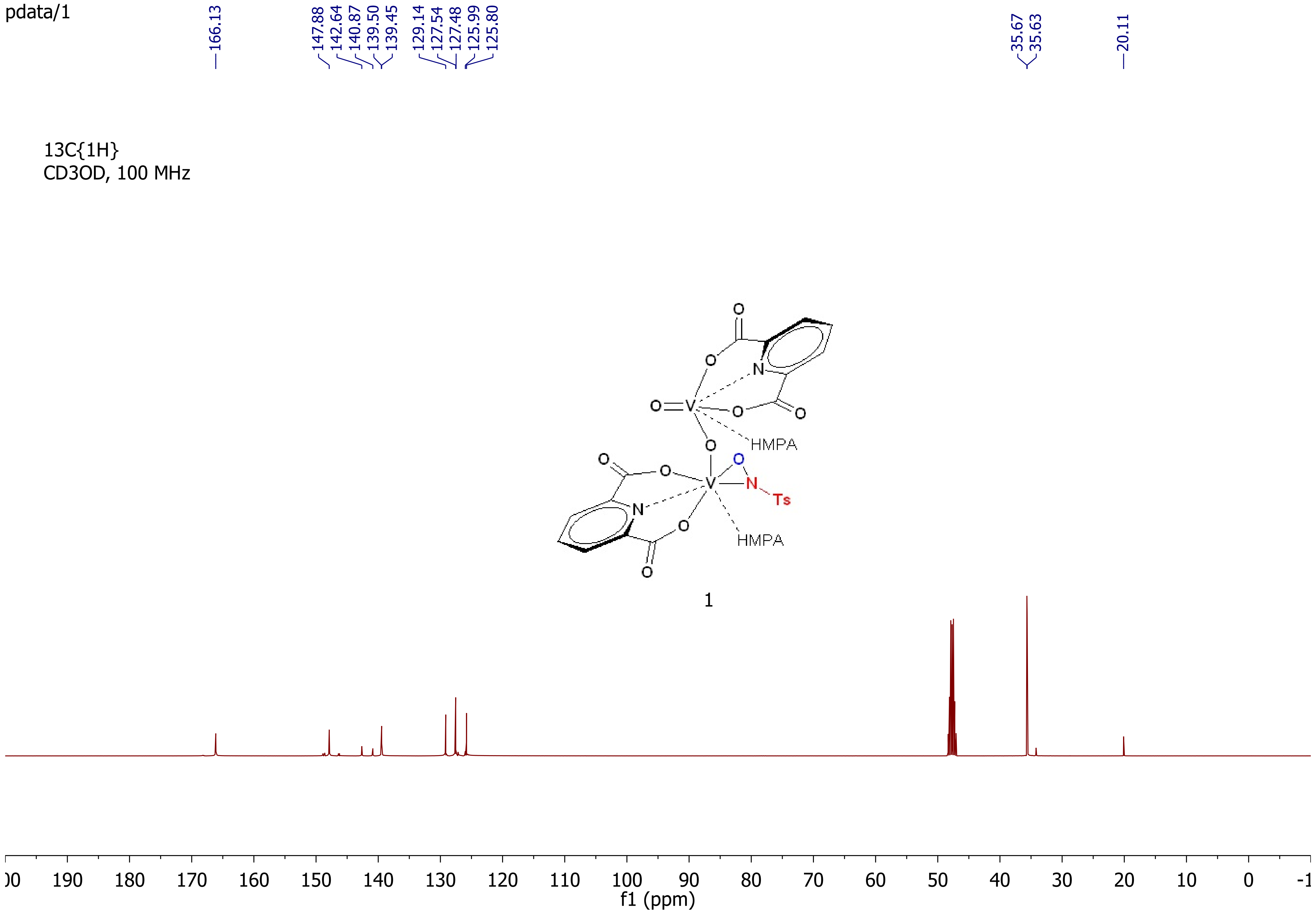
pdata/1
166.13
147.88
142.64
140.87
139.50
139.45
129.14
127.54
127.48
125.99
125.80
35.67
35.63
20.11
13C{1H}
CD3OD, 100 MHz
HMPA
HMPA
Ts
1
190 180 170 160 150 140 130 120 110 100 90 80 70 60 50 40 30 20 10 0
f1 (ppm)

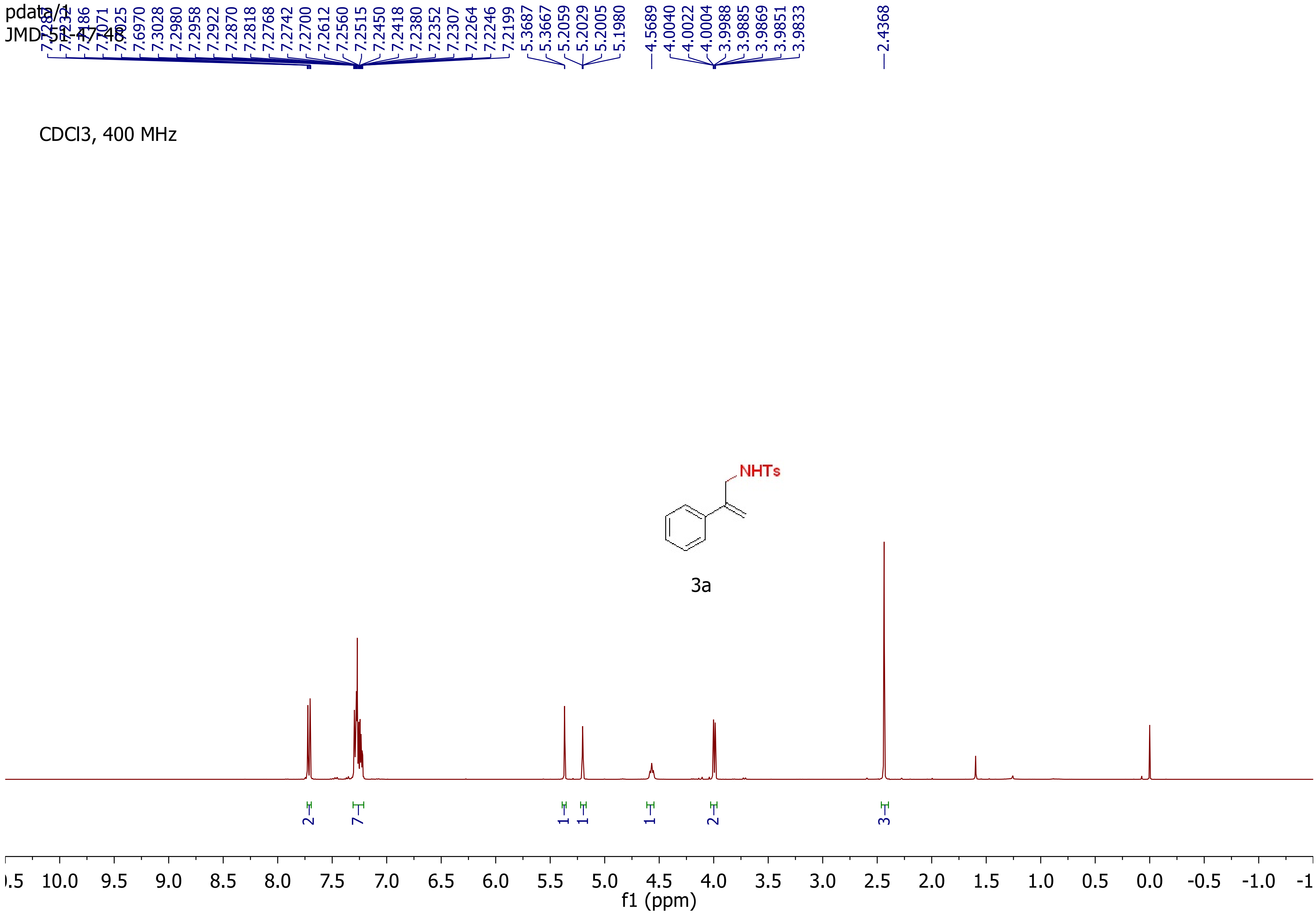

pdata/1
JMD-51-47-48
7.7287
7.7232
7.7186
7.7071
7.7025
7.6970
7.3028
7.2980
7.2958
7.2922
7.2870
7.2818
7.2768
7.2742
7.2700
7.2612
7.2560
7.2515
7.2450
7.2418
7.2380
7.2352
7.2307
7.2264
7.2246
7.2199
5.3687
5.3667
5.2059
5.2029
5.2005
5.1980
4.5689
4.0040
4.0022
4.0004
3.9988
3.9885
3.9869
3.9851
3.9833
2.4368
CDCl3, 400 MHz
NHTs
3a
2
7
1
1
1
2
3
10.0
9.5
9.0
8.5
8.0
7.5
7.0
6.5
6.0
5.5
5.0
4.5
4.0
3.5
3.0
2.5
2.0
1.5
1.0
0.5
0.0
-0.5
-1.0
f1 (ppm)

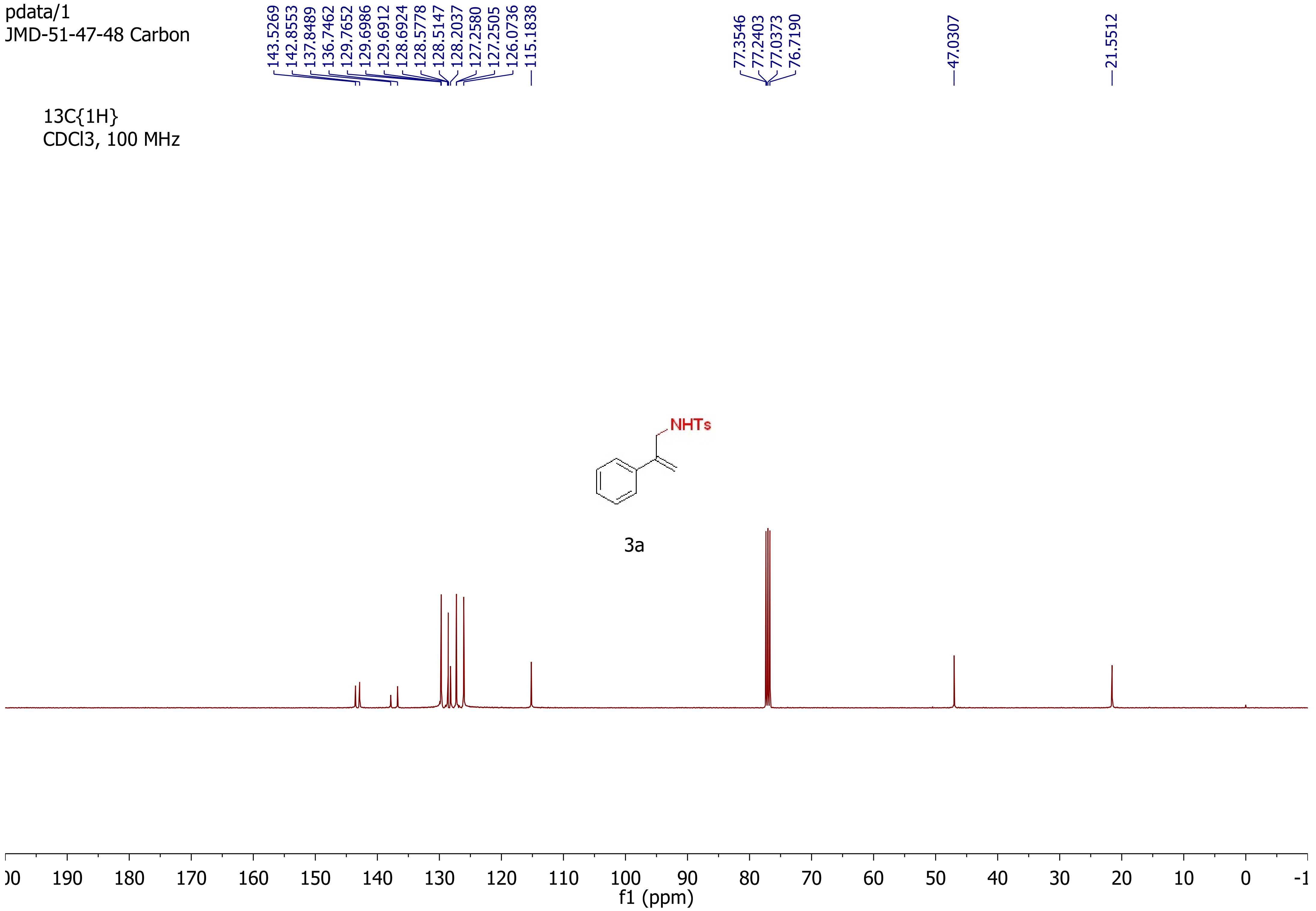
pdata/1
JMD-51-47-48 Carbon
13C{1H}
CDCl3, 100 MHz
143.5269
142.8553
137.8489
136.7462
129.7652
129.6986
129.6912
128.6924
128.5778
128.5147
128.2037
127.2580
127.2505
126.0736
115.1838
77.3546
77.2403
77.0373
76.7190
47.0307
21.5512
NHTs
3a
f1 (ppm)

pdata/1

CDCl3, 400 MHz

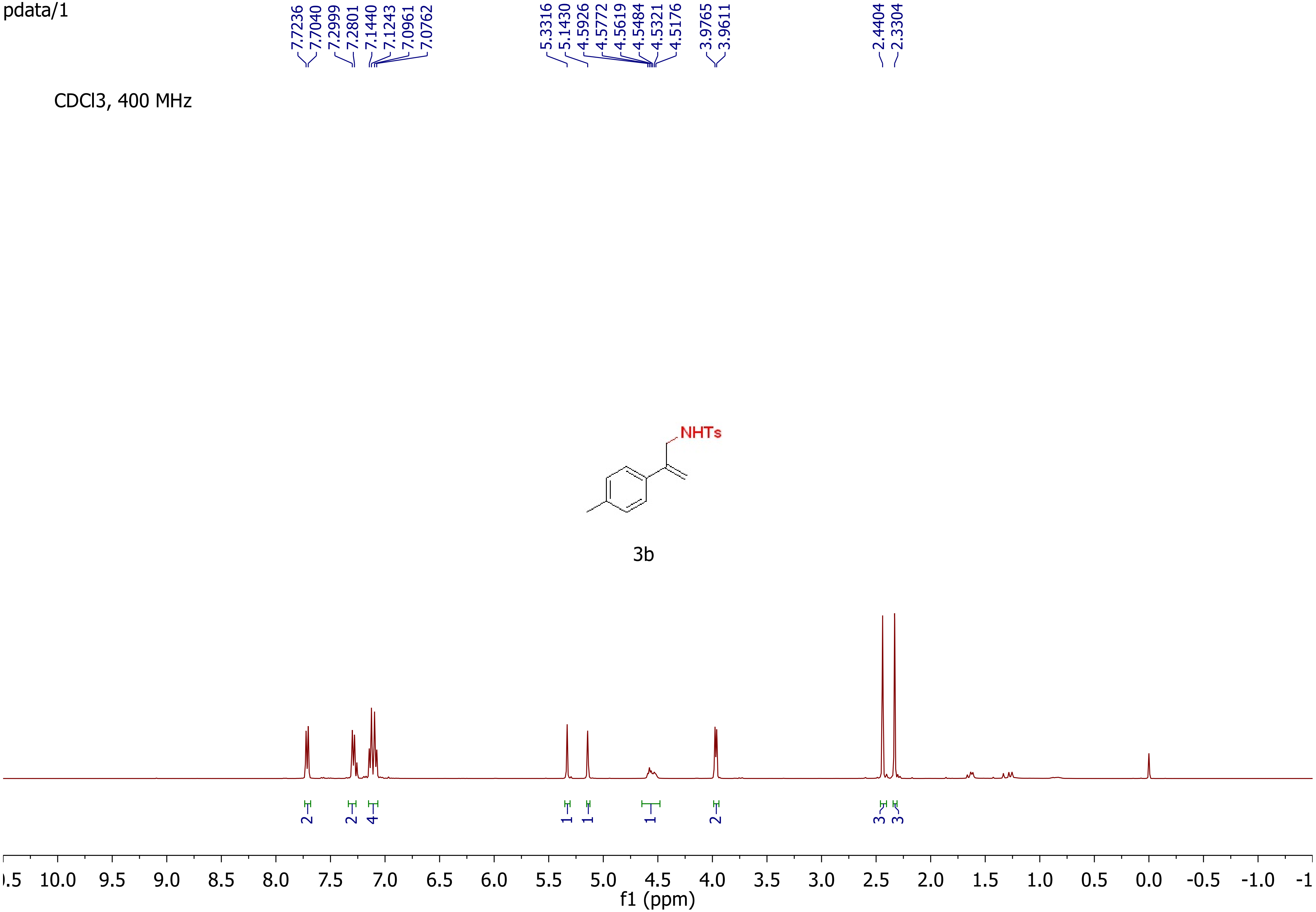

3b

pdata/1

13C{1H}
CDCl3, 100 MHz

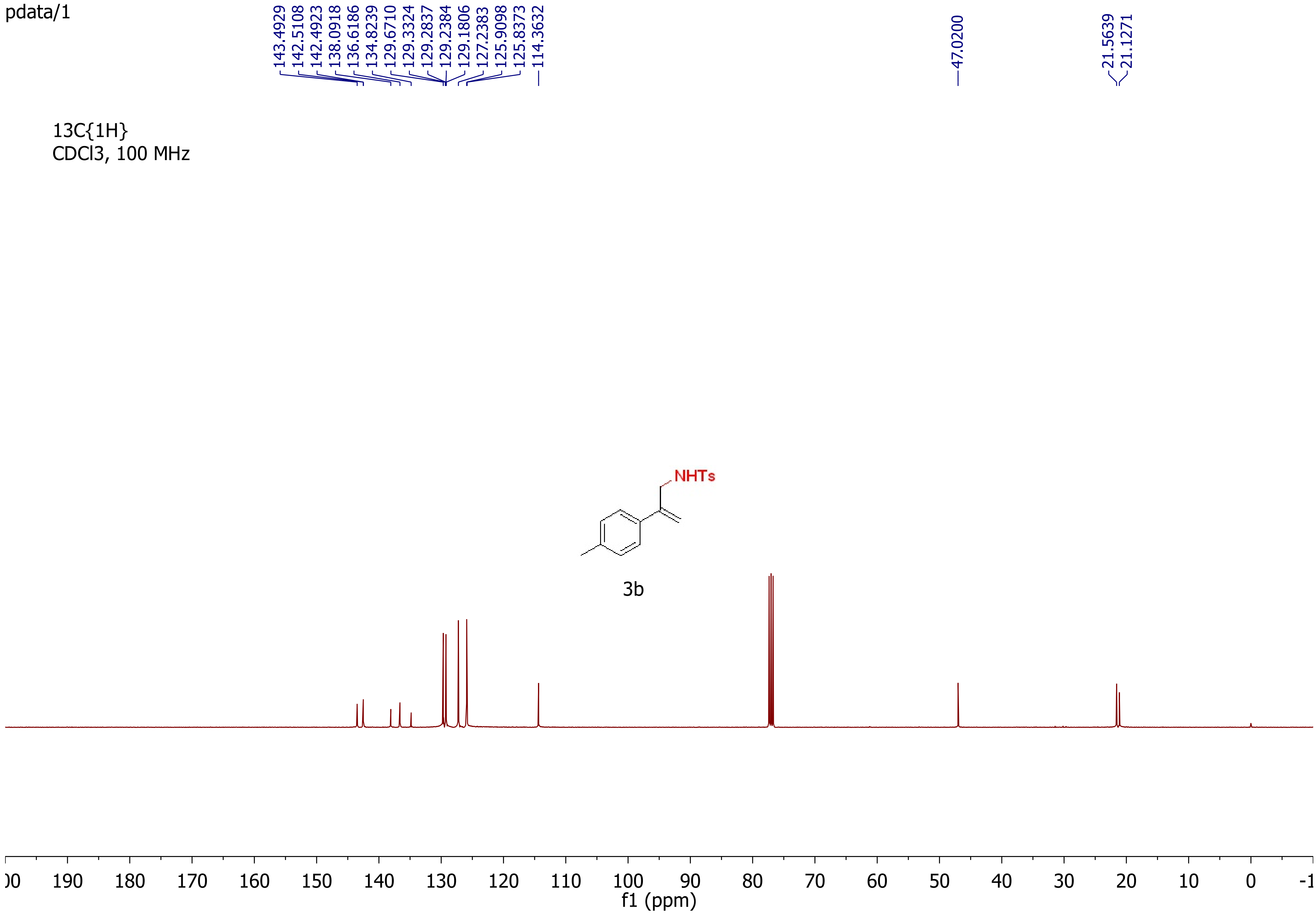

3b

pdata/1
ELD-63D-PURE

CDCl3, 400 MHz

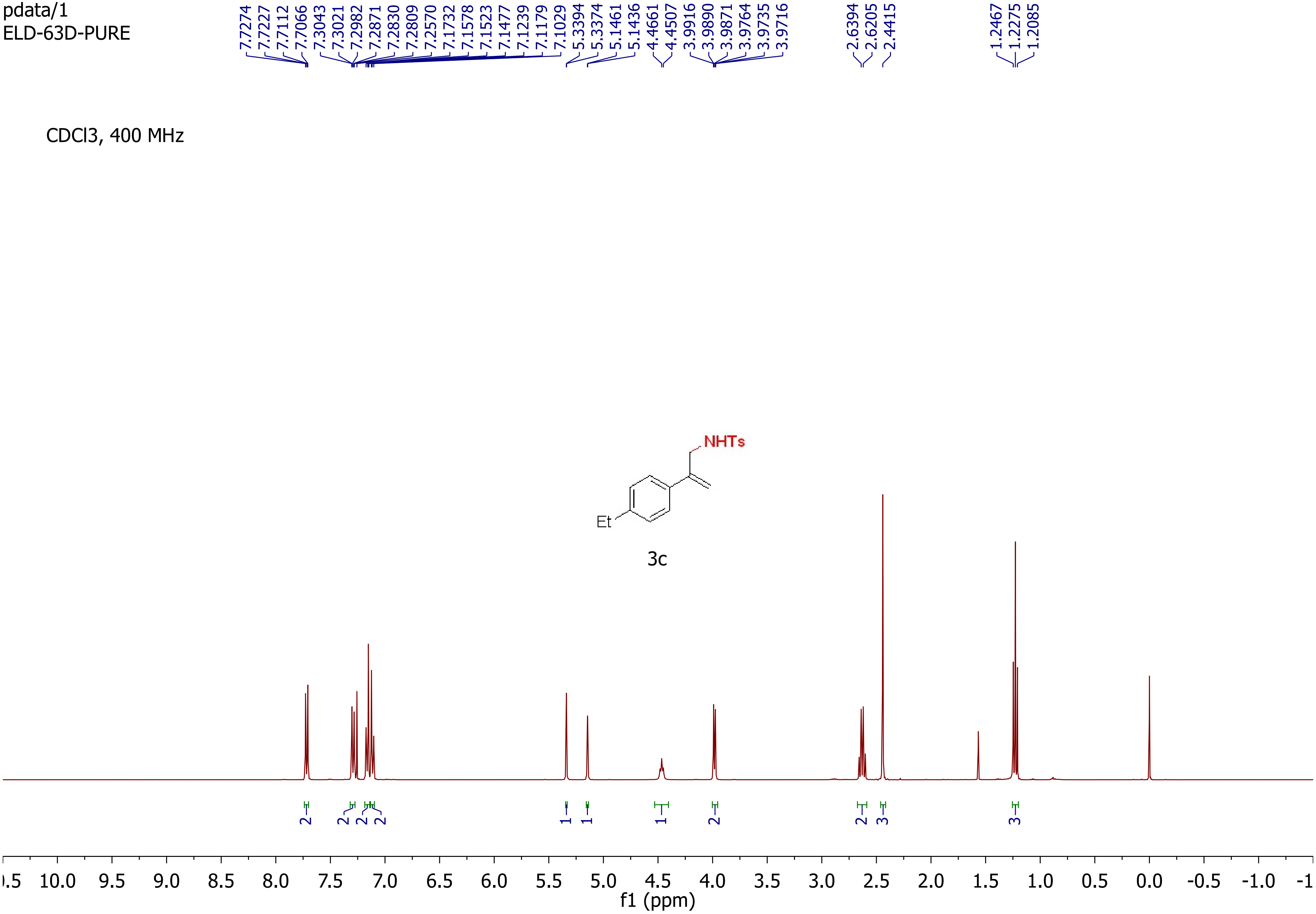

3c

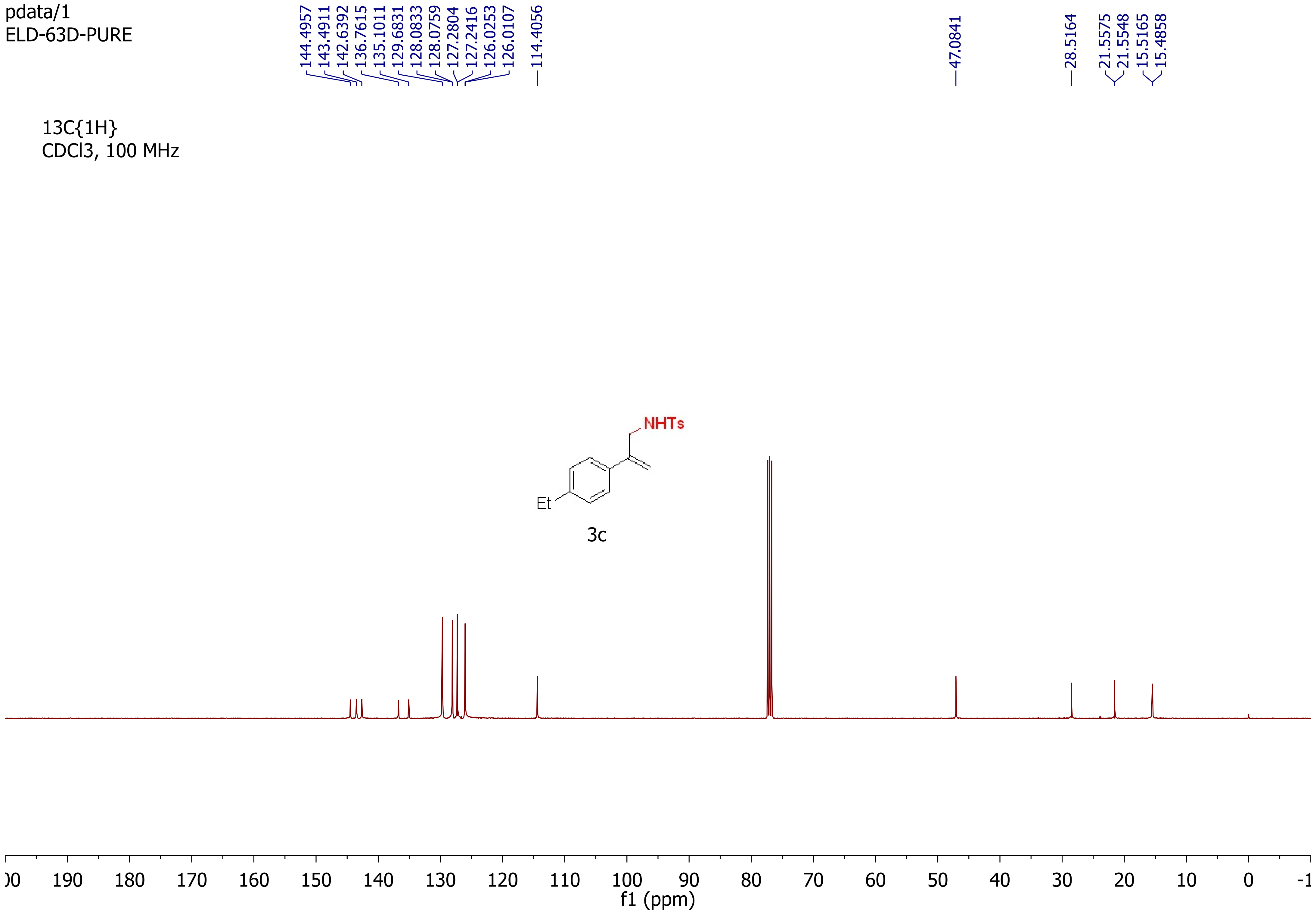
pdata/1
ELD-63D-PURE
13C{1H}
CDCl3, 100 MHz
144.4957
143.4911
142.6392
136.7615
135.1011
129.6831
128.0833
128.0759
127.2804
127.2416
126.0253
126.0107
114.4056
47.0841
28.5164
21.5575
21.5548
15.5165
15.4858
NHTs
Et
3c
190
180
170
160
150
140
130
120
110
100
90
80
70
60
50
40
30
20
10
0
f1 (ppm)

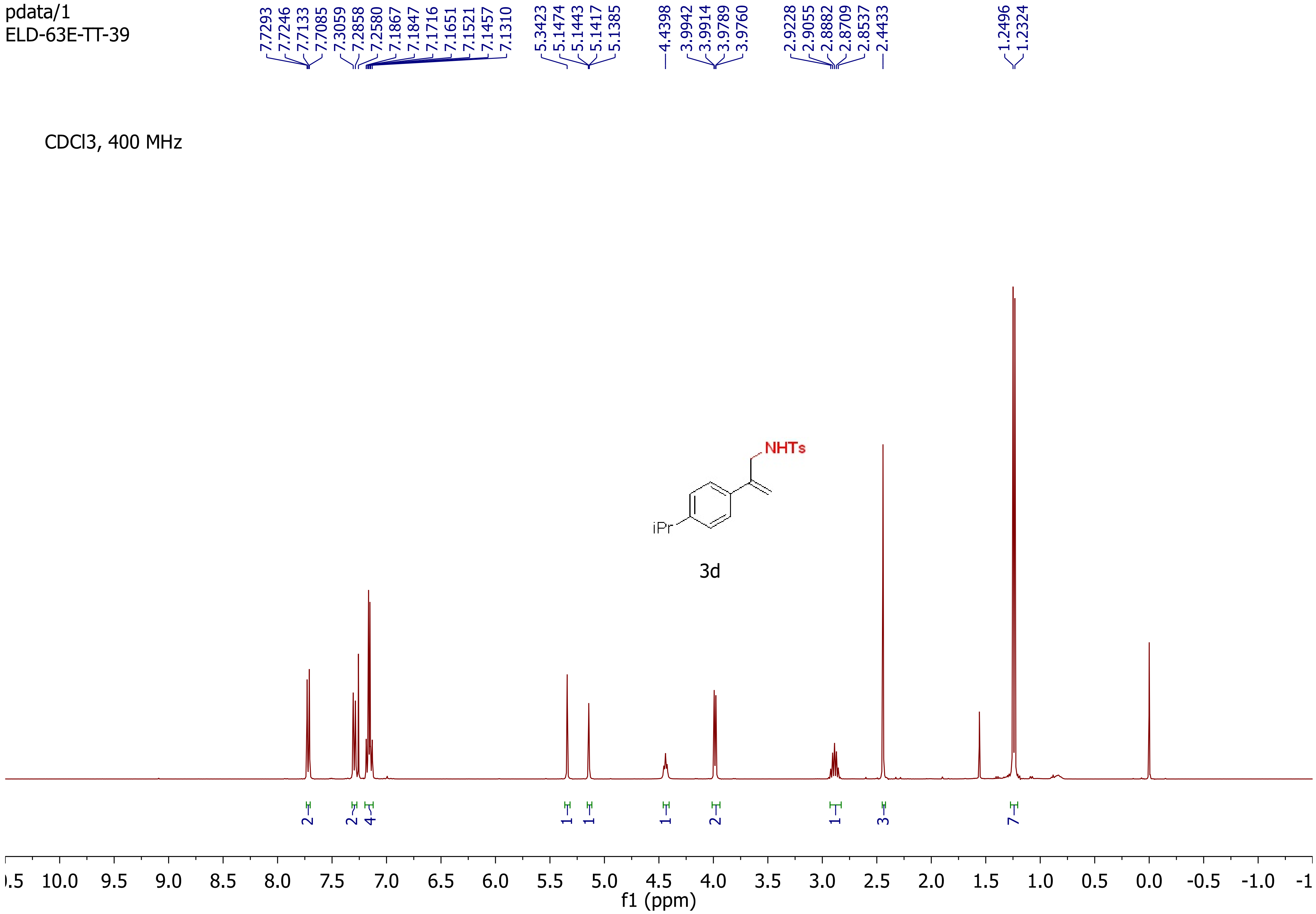
pdata/1
ELD-63E-TT-39
CDCl3, 400 MHz
7.7293
7.7246
7.7133
7.7085
7.3059
7.2858
7.2580
7.1867
7.1847
7.1716
7.1651
7.1521
7.1457
7.1310
5.3423
5.1474
5.1443
5.1417
5.1385
4.4398
3.9942
3.9914
3.9789
3.9760
2.9228
2.9055
2.8882
2.8709
2.8537
2.4433
1.2496
1.2324
NHTs
iPr
3d
f1 (ppm)

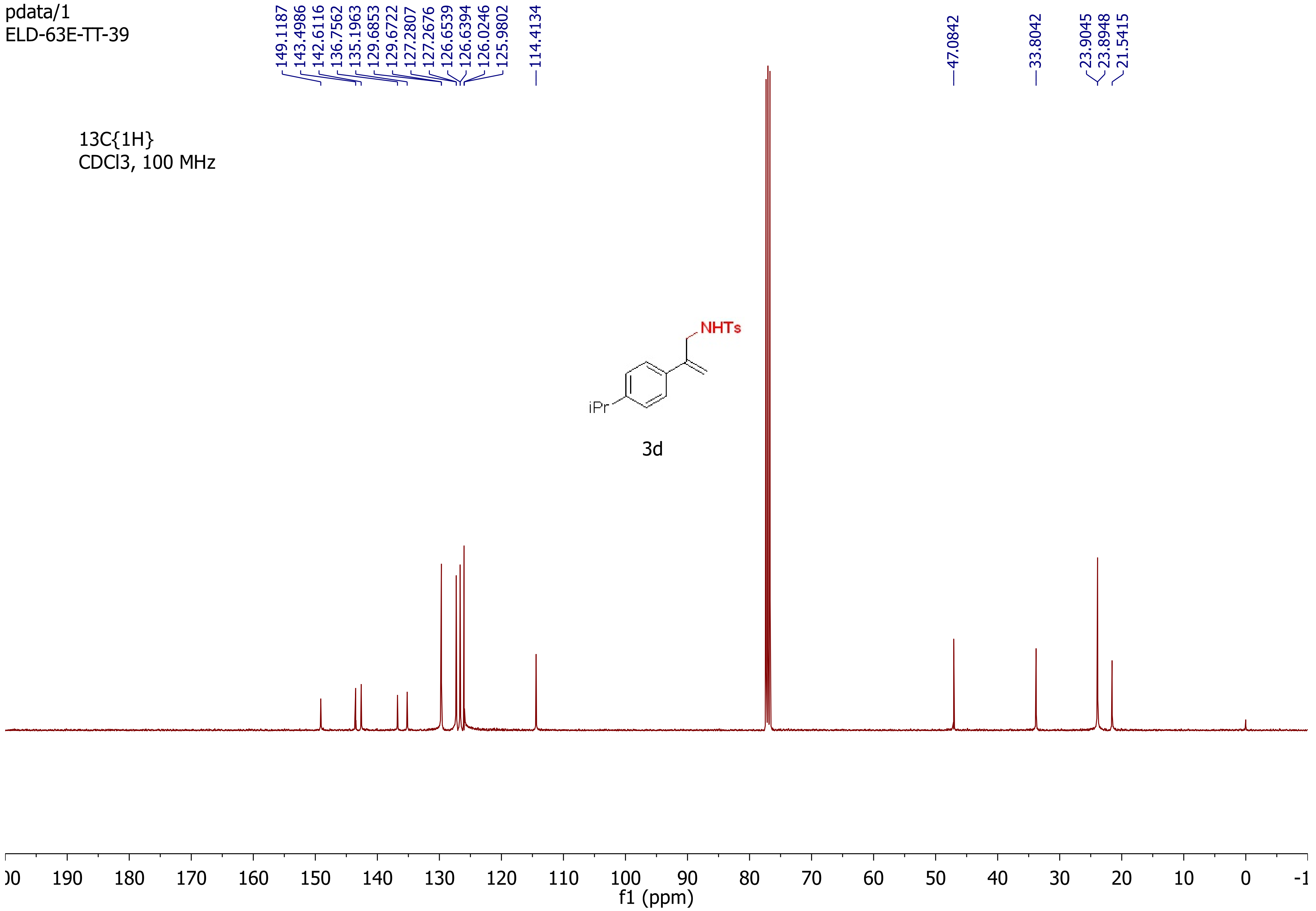
pdata/1
ELD-63E-TT-39
149.1187
143.4986
142.6116
136.7562
135.1963
129.6853
129.6722
127.2807
127.2676
126.6539
126.6394
126.0246
125.9802
114.4134
47.0842
33.8042
23.9045
23.8948
21.5415
13C{1H}
CDCl3, 100 MHz
NHTs
iPr
3d
190
180
170
160
150
140
130
120
110
100
90
80
70
60
50
40
30
20
10
0
f1 (ppm)

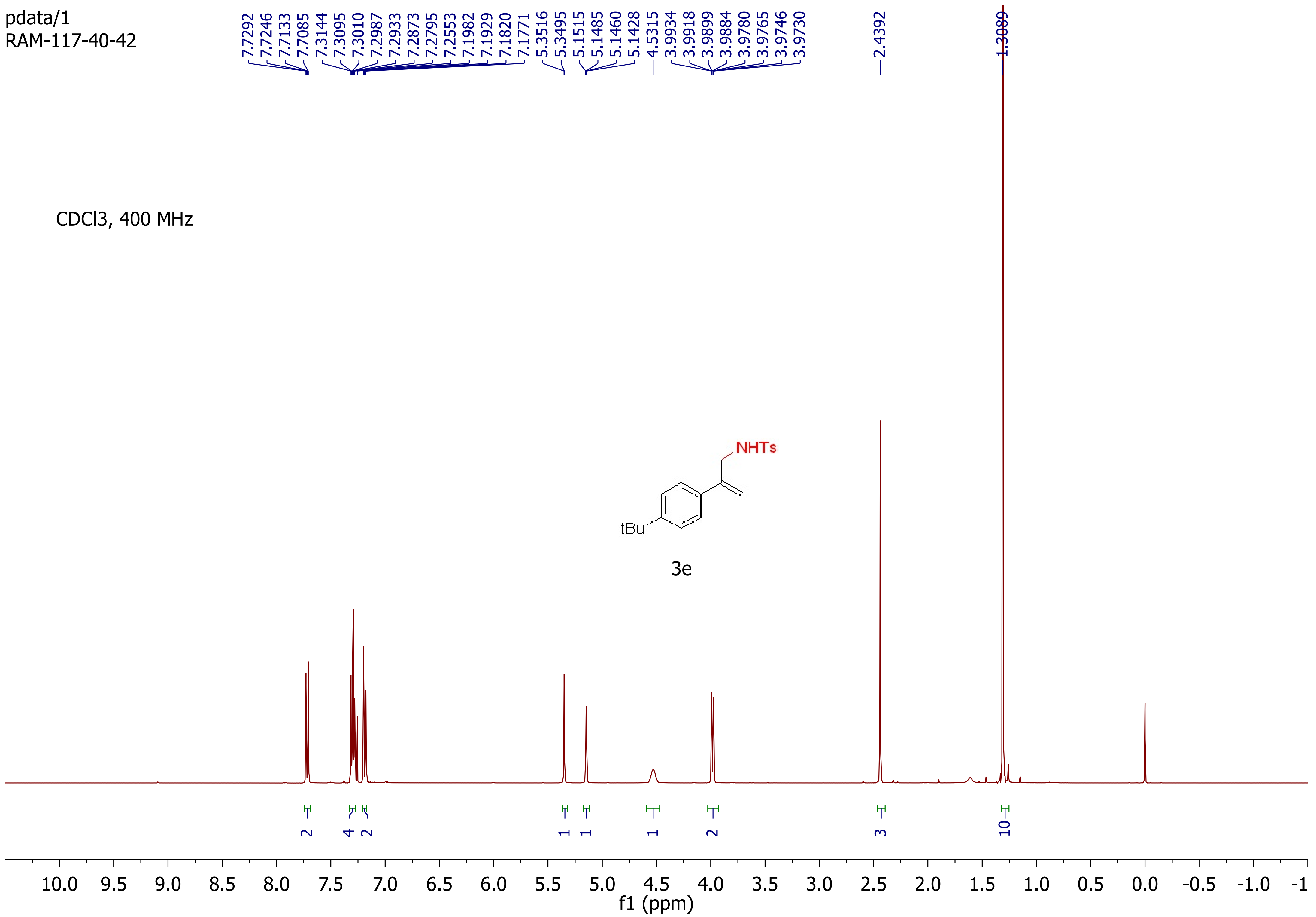
pdata/1
RAM-117-40-42
CDCl3, 400 MHz
7.7292
7.7246
7.7133
7.7085
7.3144
7.3095
7.3010
7.2987
7.2933
7.2873
7.2795
7.2553
7.1982
7.1929
7.1820
7.1771
5.3516
5.3495
5.1515
5.1485
5.1460
5.1428
4.5315
3.9934
3.9918
3.9899
3.9884
3.9780
3.9765
3.9746
3.9730
2.4392
1.3089
NHTs
tBu
3e
2
4
2
1
1
1
2
3
10
f1 (ppm)

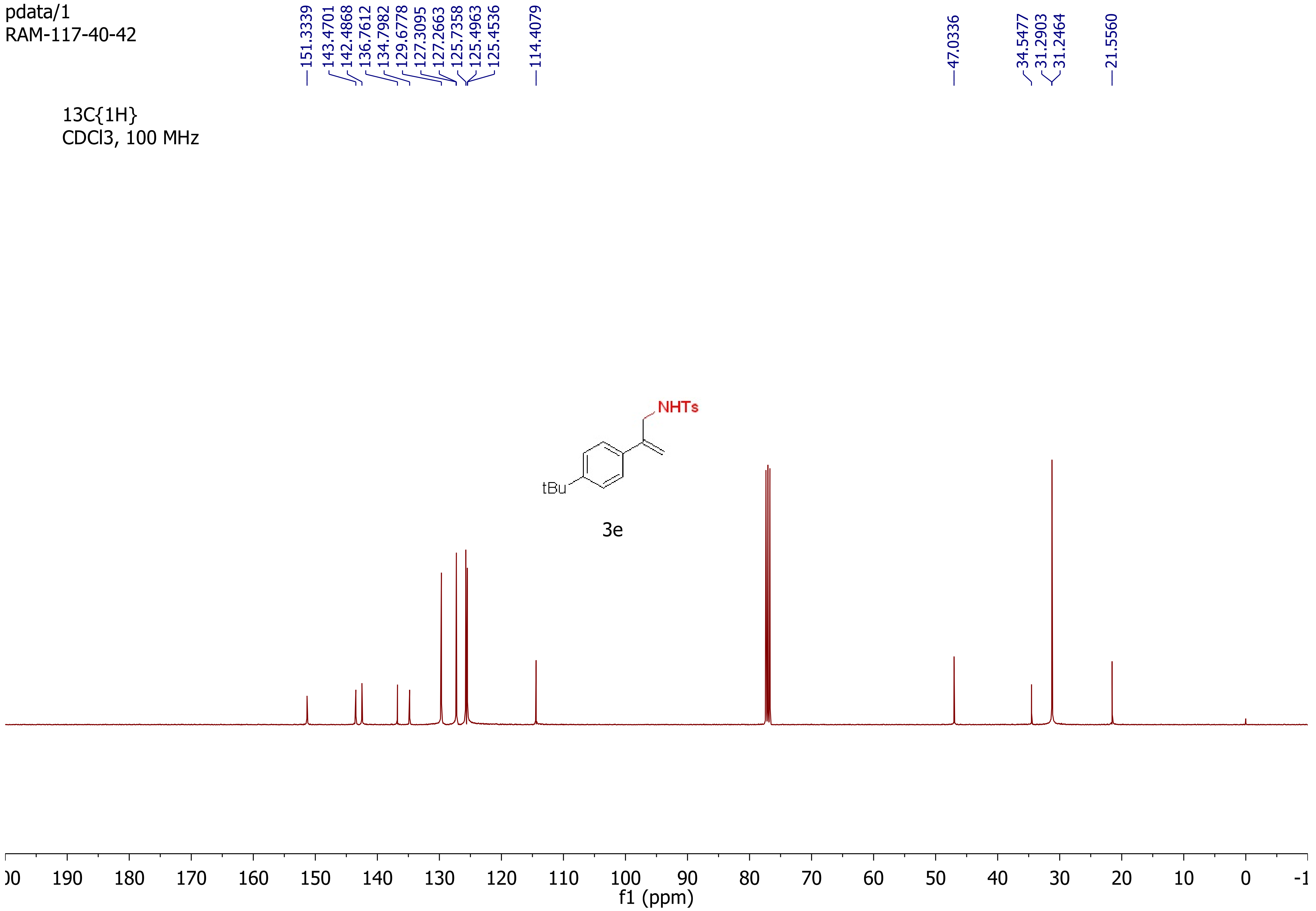
pdata/1
RAM-117-40-42
13C{1H}
CDCl3, 100 MHz
151.3339
143.4701
142.4868
136.7612
134.7982
129.6778
127.3095
127.2663
125.7358
125.4963
125.4536
114.4079
47.0336
34.5477
31.2903
31.2464
21.5560
NHTs
tBu
3e
f1 (ppm)
190
180
170
160
150
140
130
120
110
100
90
80
70
60
50
40
30
20
10
0

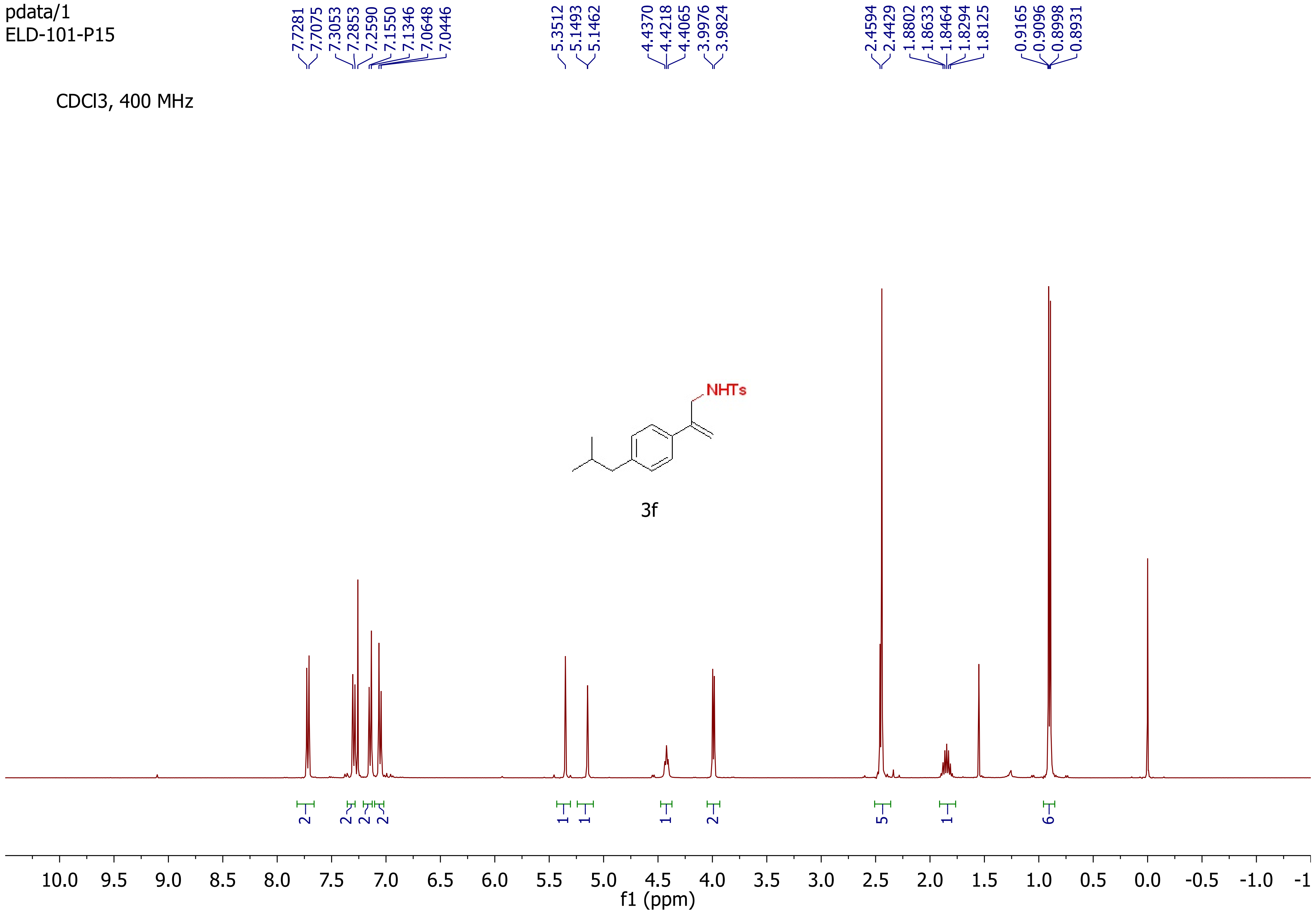
pdata/1
ELD-101-P15
CDCl3, 400 MHz
7.7281
7.7075
7.3053
7.2853
7.2590
7.1550
7.1346
7.0648
7.0446
5.3512
5.1493
5.1462
4.4370
4.4218
4.4065
3.9976
3.9824
2.4594
2.4429
1.8802
1.8633
1.8464
1.8294
1.8125
0.9165
0.9096
0.8998
0.8931
NHTs
3f
2
2
2
2
1
1
1
2
5
1
6
10.0 9.5 9.0 8.5 8.0 7.5 7.0 6.5 6.0 5.5 5.0 4.5 4.0 3.5 3.0 2.5 2.0 1.5 1.0 0.5 0.0 -0.5 -1.0 -1
f1 (ppm)

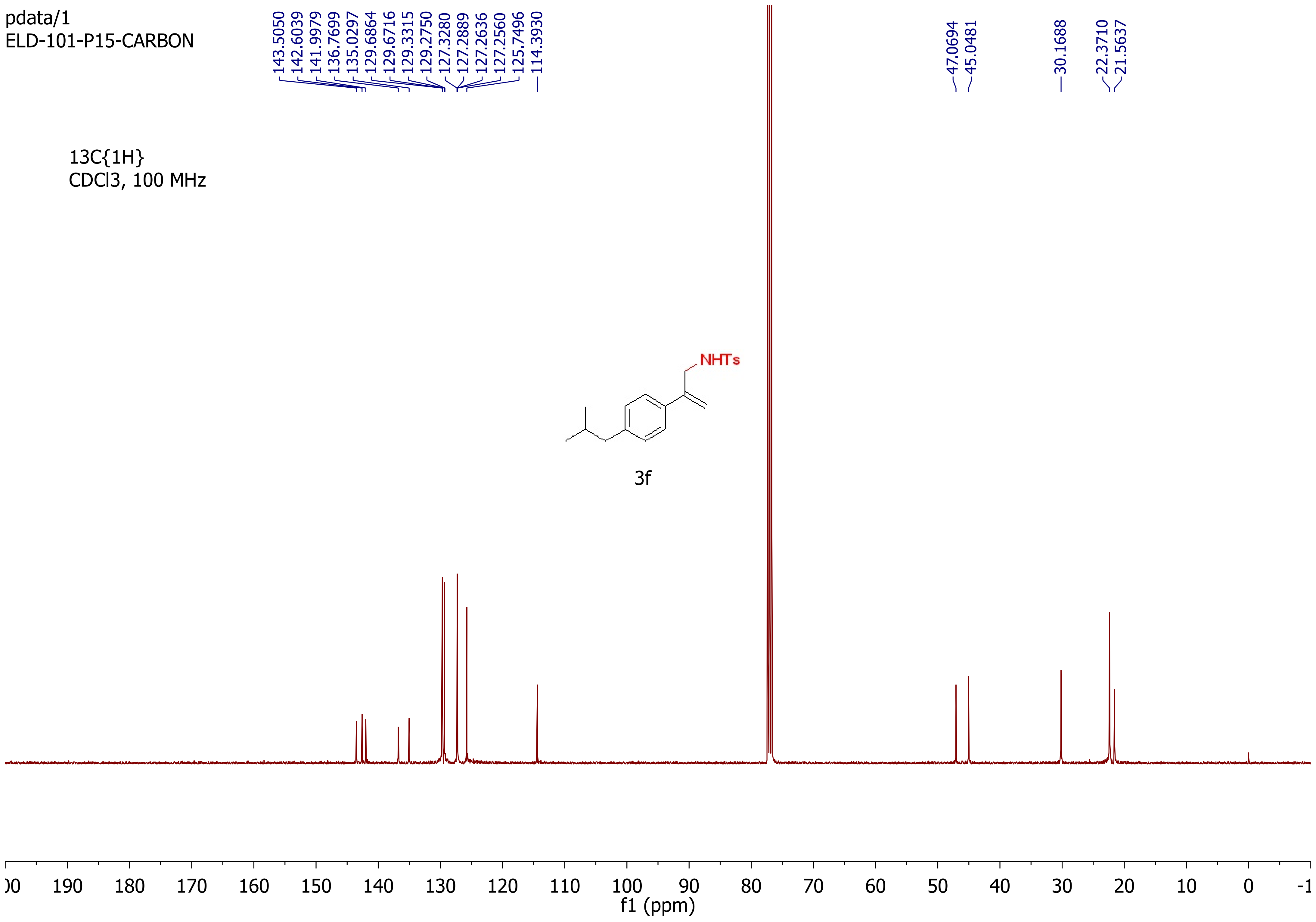
pdata/1
ELD-101-P15-CARBON
143.5050
142.6039
141.9979
136.7699
135.0297
129.6864
129.6716
129.3315
129.2750
127.3280
127.2889
127.2636
127.2560
125.7496
114.3930
47.0694
45.0481
30.1688
22.3710
21.5637
13C{1H}
CDCl3, 100 MHz
NHTs
3f
f1 (ppm)

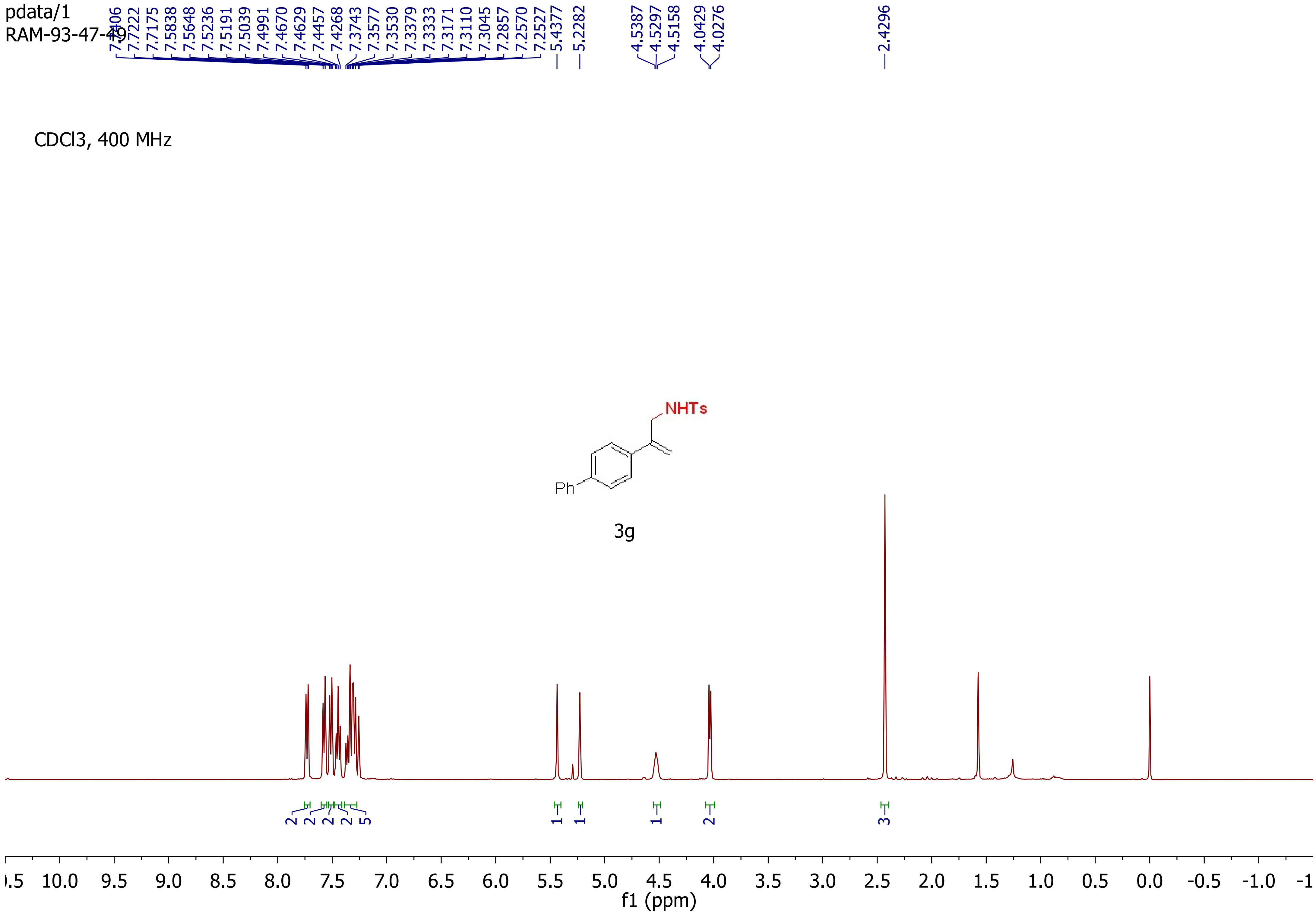

pdata/1
RAM-93-47-49
7.7406
7.7222
7.7175
7.5838
7.5648
7.5236
7.5191
7.5039
7.4991
7.4670
7.4629
7.4457
7.4268
7.3743
7.3577
7.3530
7.3379
7.3333
7.3171
7.3110
7.3045
7.2857
7.2570
7.2527
5.4377
5.2282
4.5387
4.5297
4.5158
4.0429
4.0276
2.4296
CDCl3, 400 MHz
NHTs
Ph
3g
f1 (ppm)

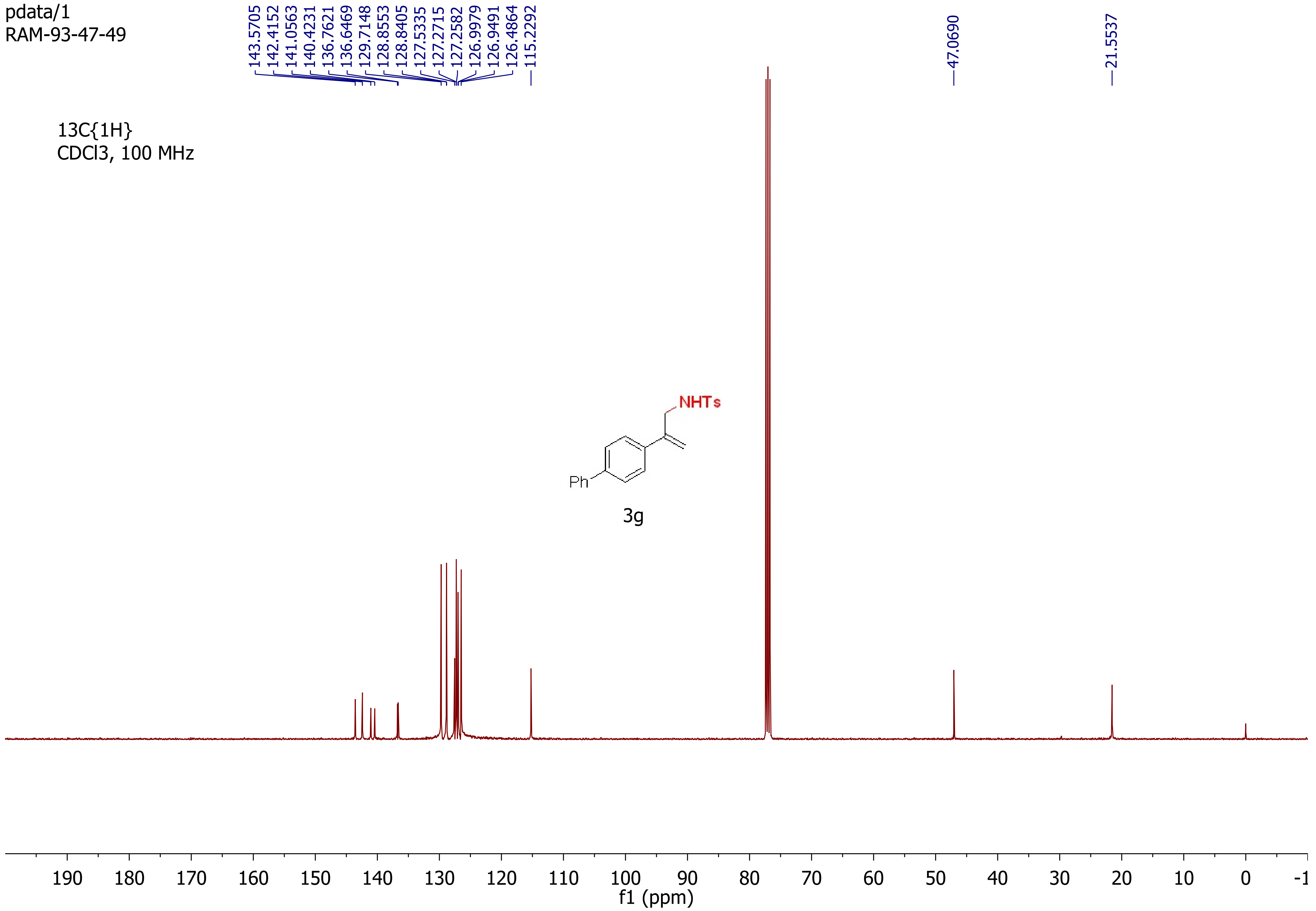
pdata/1
RAM-93-47-49
143.5705
142.4152
141.0563
140.4231
136.7621
136.6469
129.7148
128.8553
128.8405
127.5335
127.2715
127.2582
126.9979
126.9491
126.4864
115.2292
47.0690
21.5537
13C{1H}
CDCl3, 100 MHz
NHTs
Ph
3g
190
180
170
160
150
140
130
120
110
100
90
80
70
60
50
40
30
20
10
0
-1
f1 (ppm)

pdata/1
RAM-92-57

CDCl3, 400 MHz

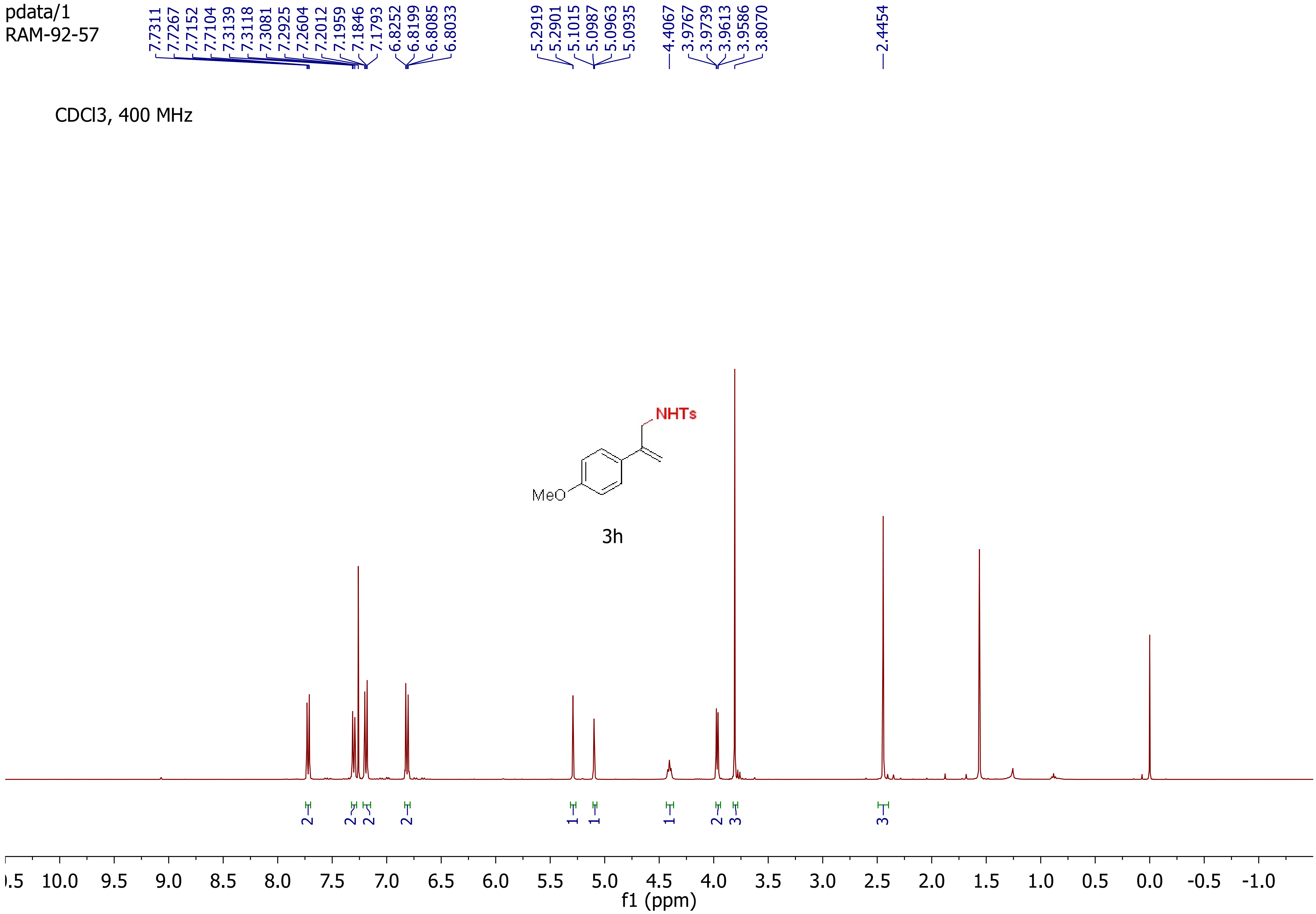

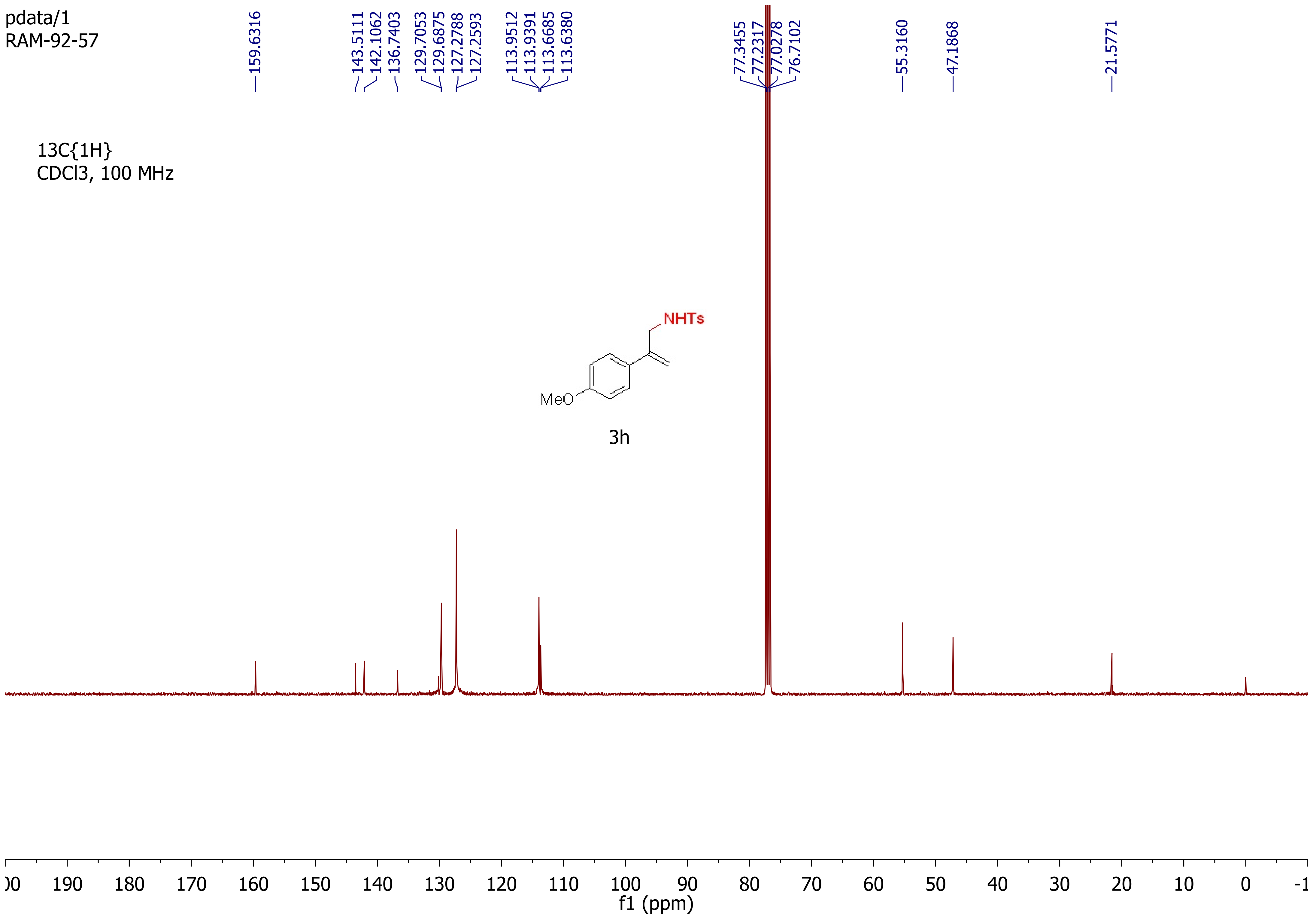

pdata/1
RAM-92-57
159.6316
143.5111
142.1062
136.7403
129.7053
129.6875
127.2788
127.2593
113.9512
113.9391
113.6685
113.6380
77.3455
77.2317
77.0278
76.7102
55.3160
47.1868
21.5771
13C{1H}
CDCl3, 100 MHz
NHTs
MeO
3h
f1 (ppm)

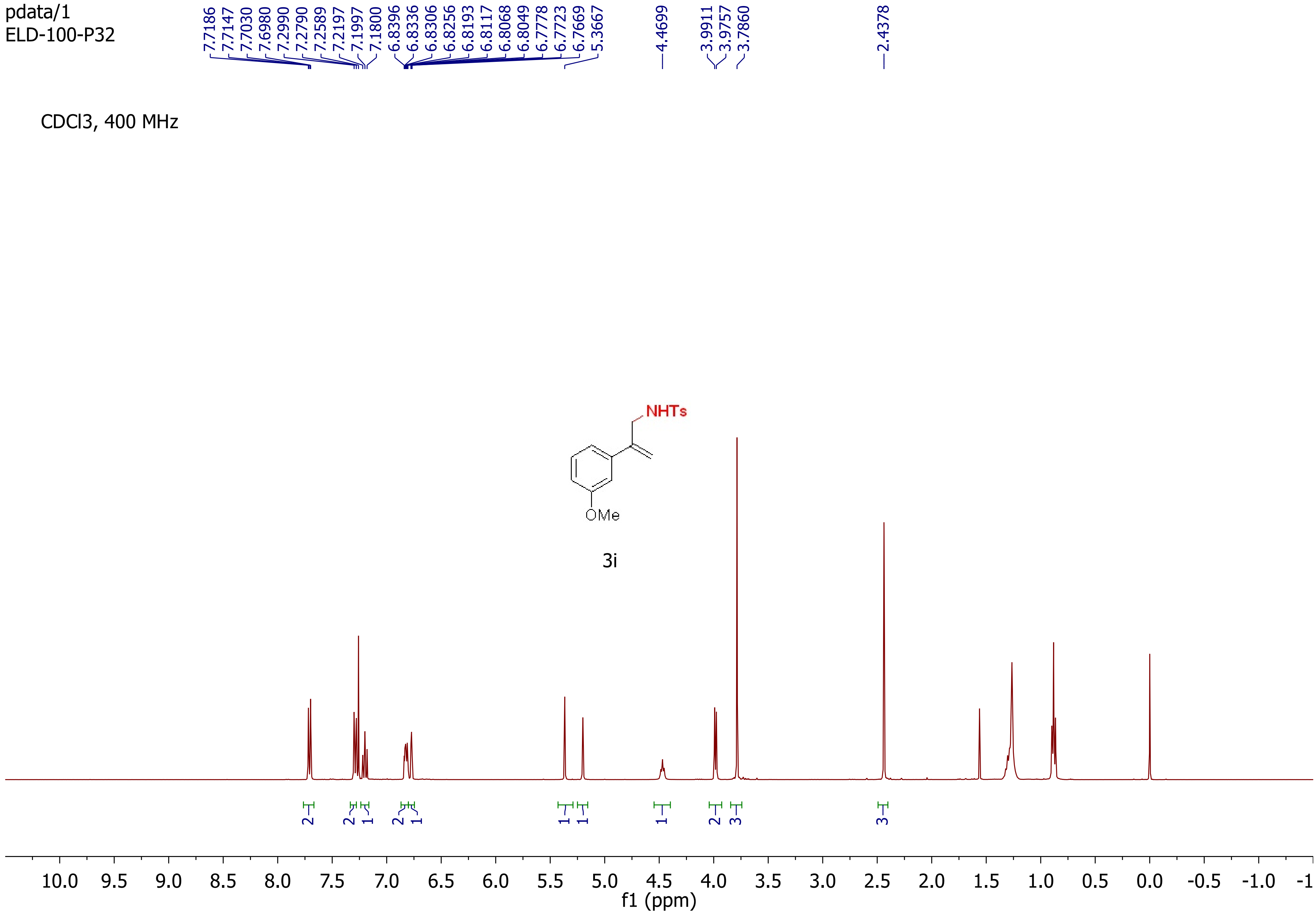
pdata/1
ELD-100-P32
CDCl3, 400 MHz
7.7186
7.7147
7.7030
7.6980
7.2990
7.2790
7.2589
7.2197
7.1997
7.1800
6.8396
6.8336
6.8306
6.8256
6.8193
6.8117
6.8068
6.8049
6.7778
6.7723
6.7669
5.3667
4.4699
3.9911
3.9757
3.7860
2.4378
2
2
1
2
1
1
1
1
2
3
3
10.0
9.5
9.0
8.5
8.0
7.5
7.0
6.5
6.0
5.5
5.0
4.5
4.0
3.5
3.0
2.5
2.0
1.5
1.0
0.5
0.0
-0.5
-1.0
-1
f1 (ppm)
NHTs
OMe

3i

pdata/1
ELD-100-P32-CARBON

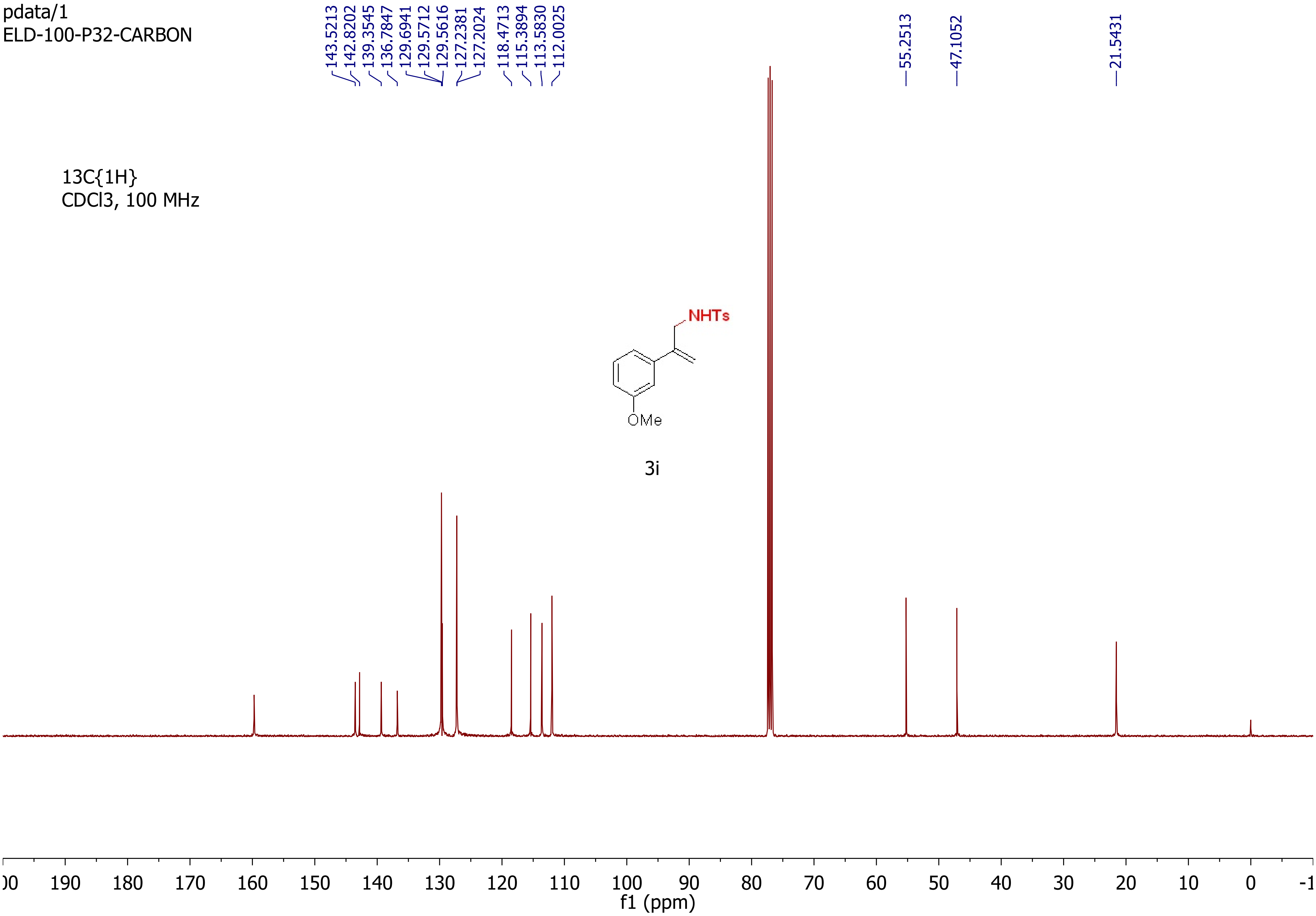

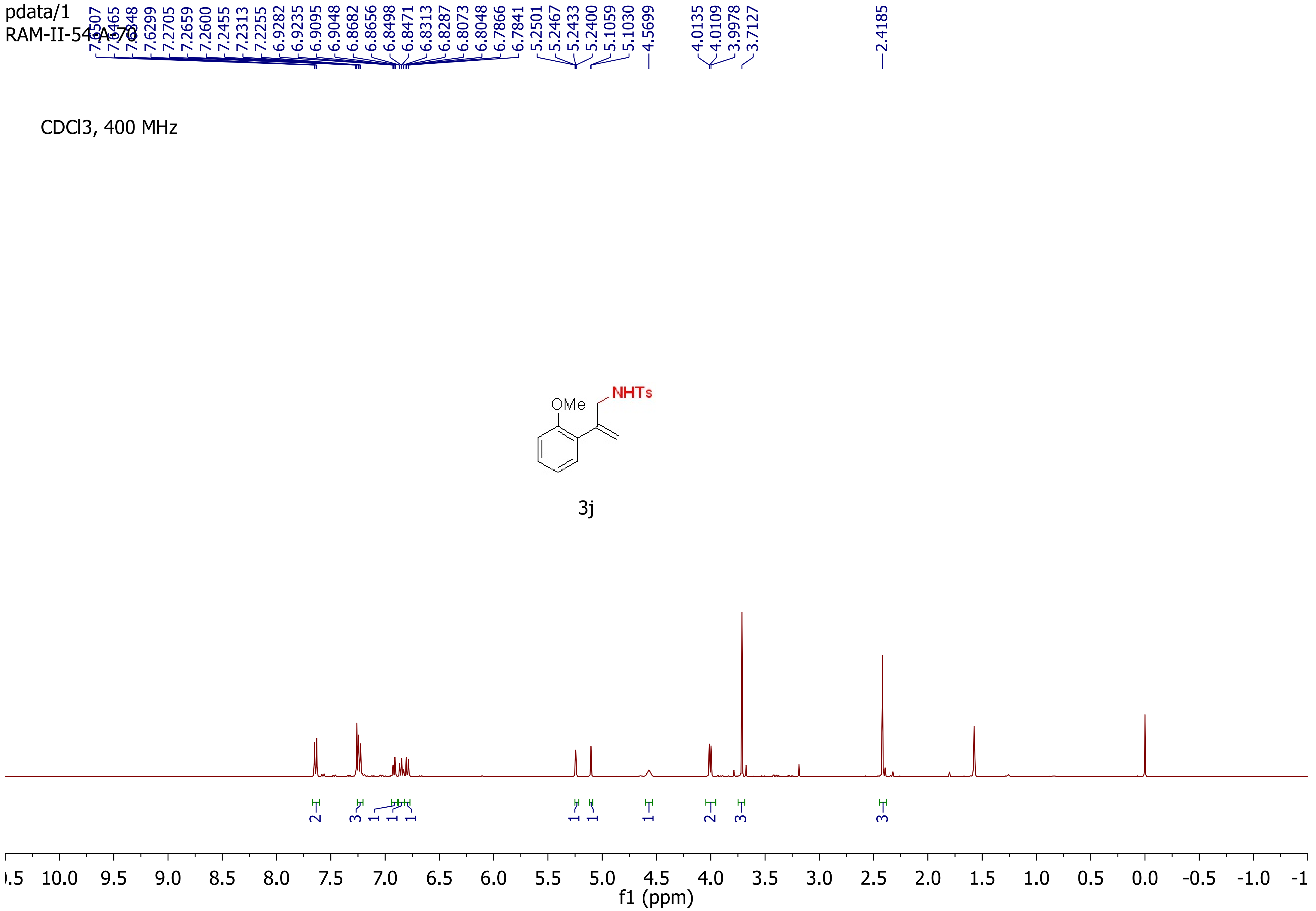
pdata/1
RAM-II-54-A-70
7.6465
7.6299
7.2705
7.2659
7.2600
7.2455
7.2313
7.2255
6.9282
6.9235
6.9095
6.9048
6.8682
6.8656
6.8498
6.8471
6.8313
6.8287
6.8073
6.8048
6.7866
6.7841
5.2501
5.2467
5.2433
5.2400
5.1059
5.1030
4.5699
4.0135
4.0109
3.9978
3.7127
2.4185
CDCl3, 400 MHz
OMe
NHTs
3j
f1 (ppm)

pdata/1
RAM-II-54-A-70

13C{1H}
CDCl3, 100 MHz

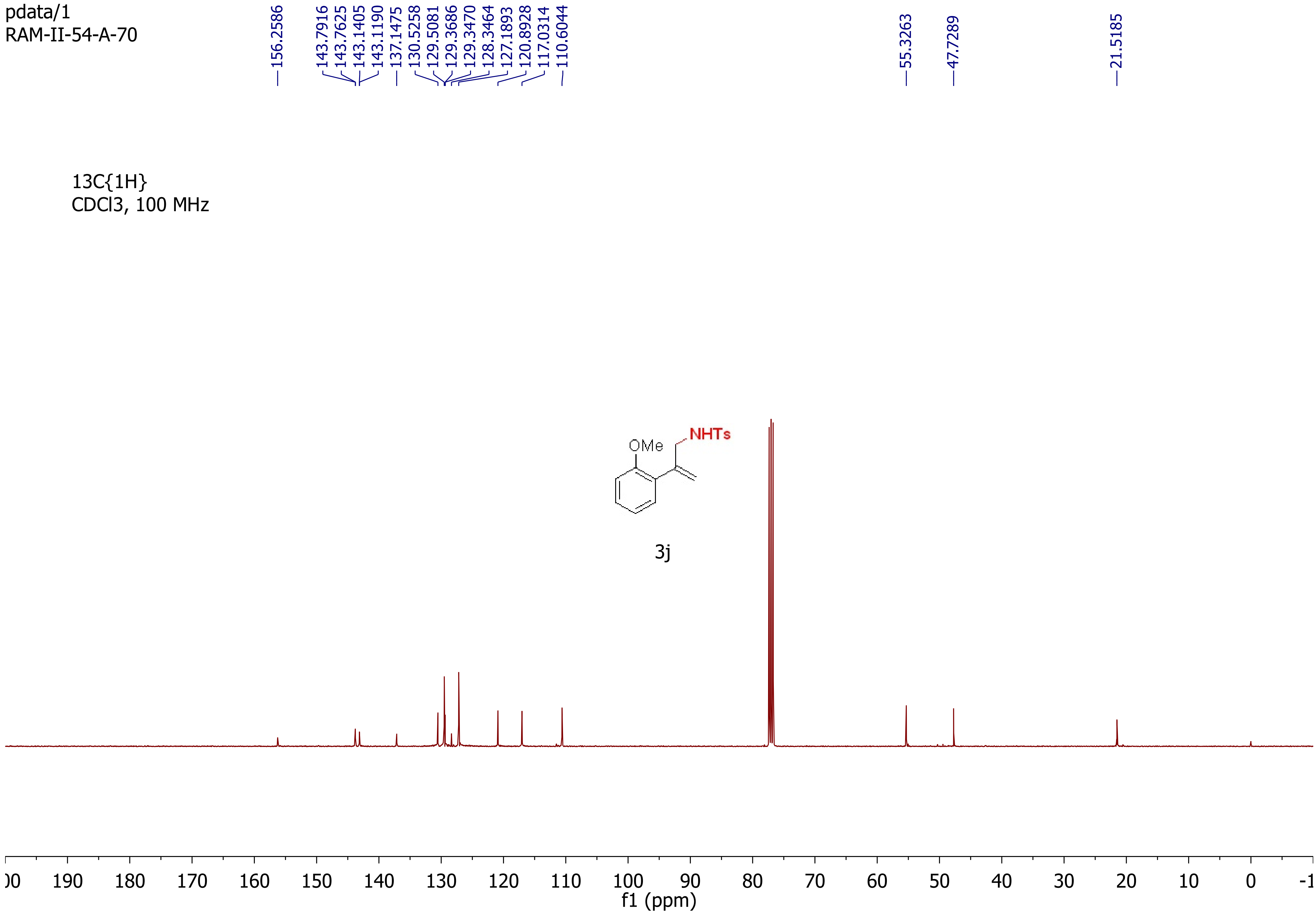

pdata/1
RAM-II-14D-48

CDCl3, 400 MHz

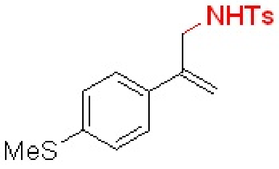

3l

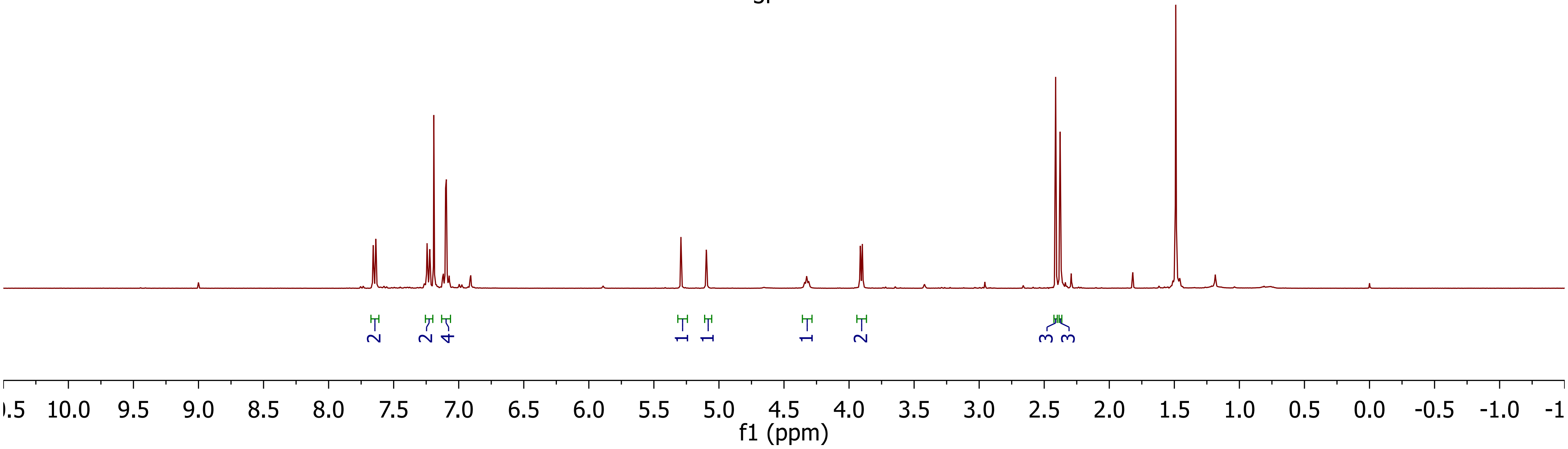

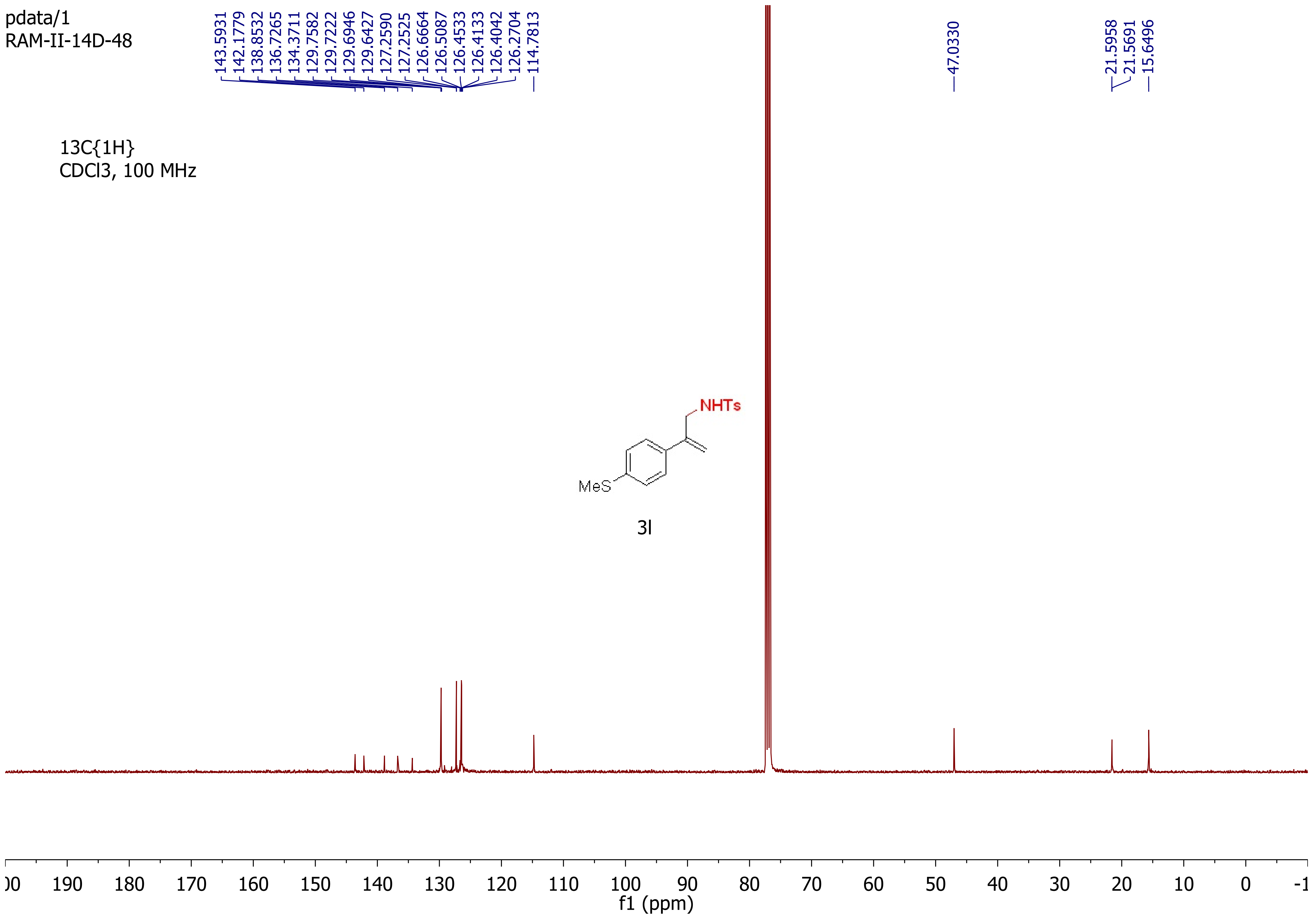
pdata/1
RAM-II-14D-48
13C{1H}
CDCl3, 100 MHz
143.5931
142.1779
138.8532
136.7265
134.3711
129.7582
129.7222
129.6946
129.6427
127.2590
127.2525
126.6664
126.5087
126.4533
126.4133
126.4042
126.2704
114.7813
47.0330
21.5958
21.5691
15.6496
NHTs
MeS
f1 (ppm)

3l

pdata/1
ELD-99-P40

CDCl3, 400 MHz

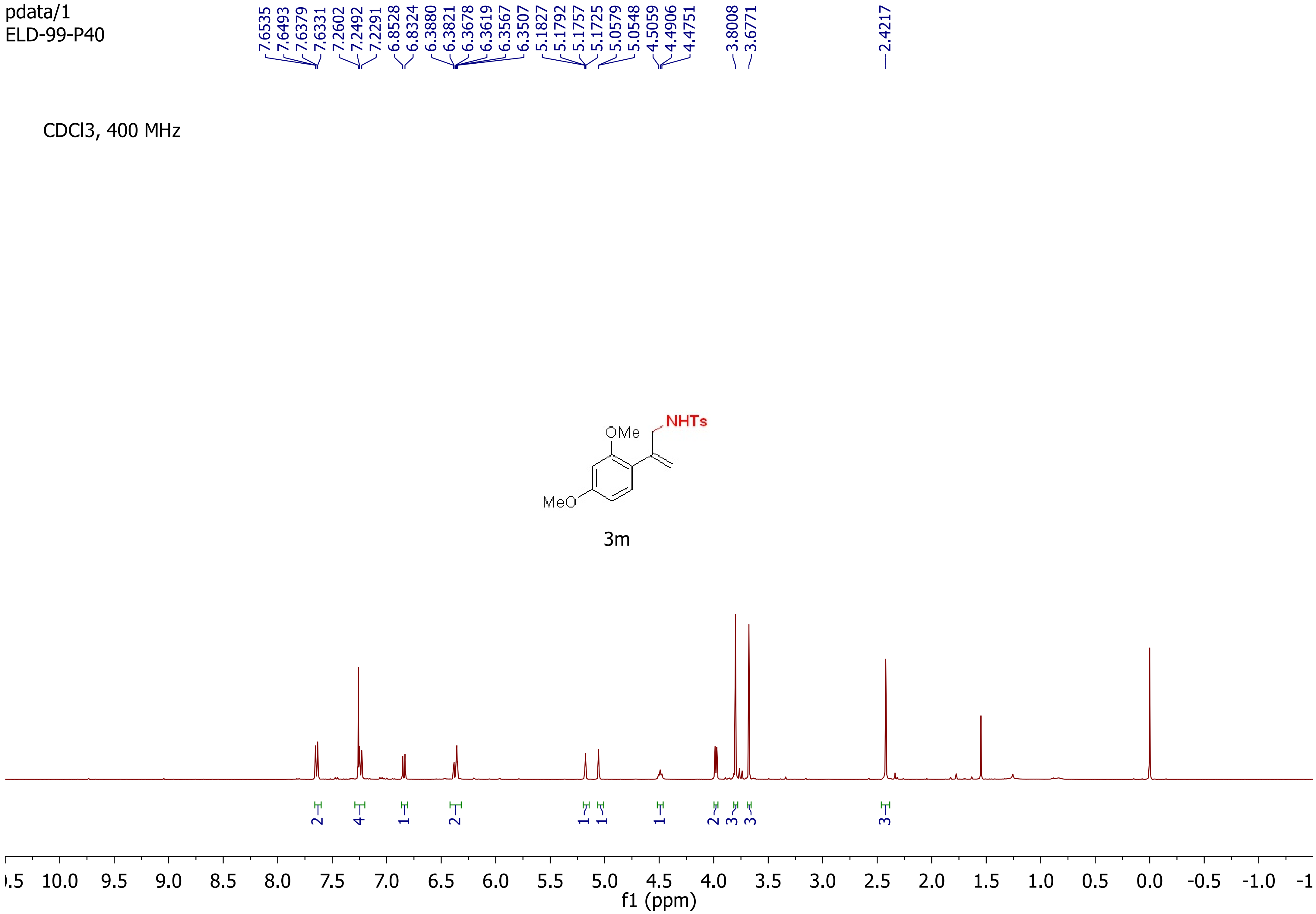

3m

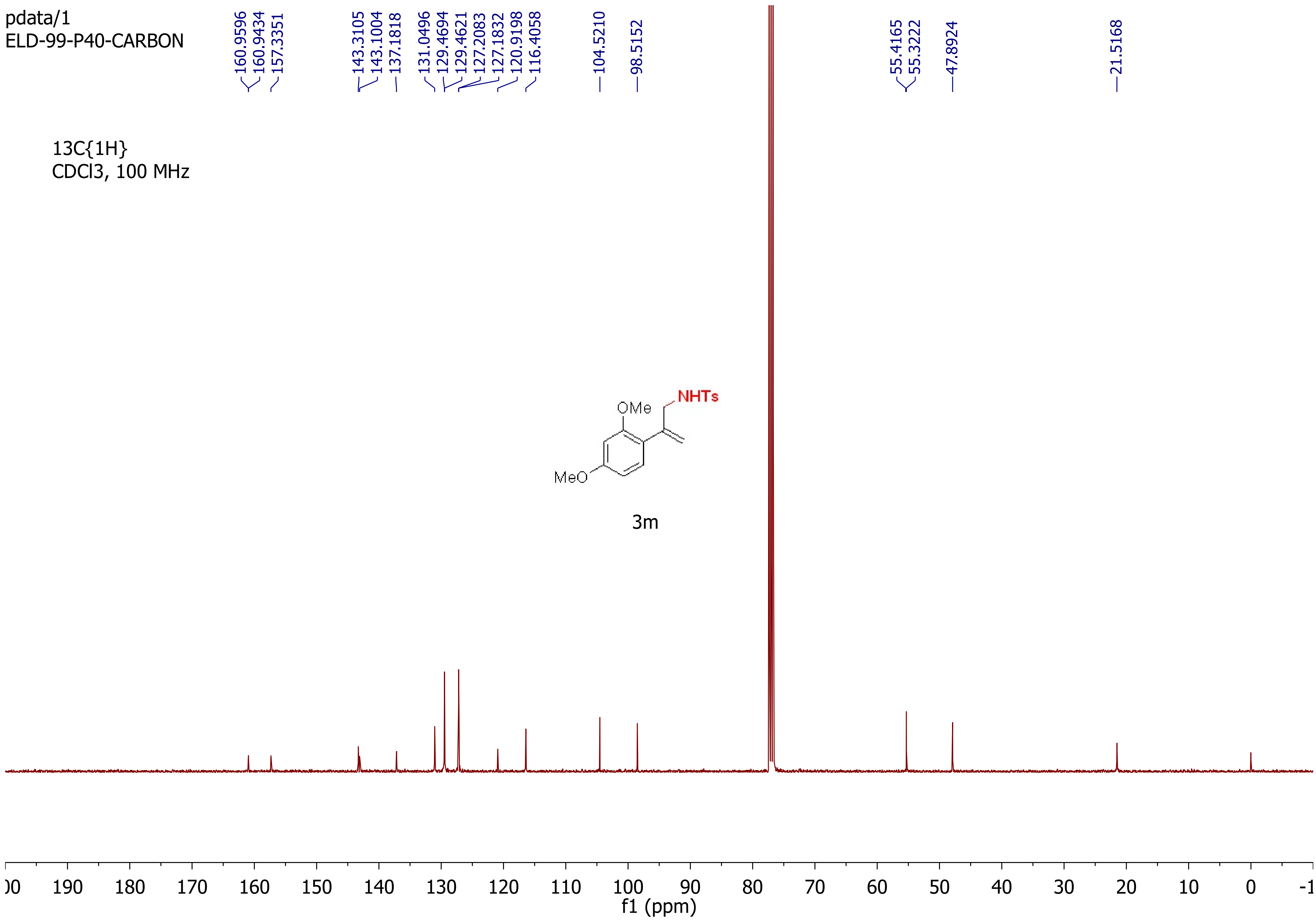
pdata/1
ELD-99-P40-CARBON
13C{1H}
CDCl3, 100 MHz
160.9596
160.9434
157.3351
143.3105
143.1004
137.1818
131.0496
129.4694
129.4621
127.2083
127.1832
120.9198
116.4058
104.5210
98.5152
55.4165
55.3222
47.8924
21.5168
OMe
NHTs
MeO
3m
f1 (ppm)

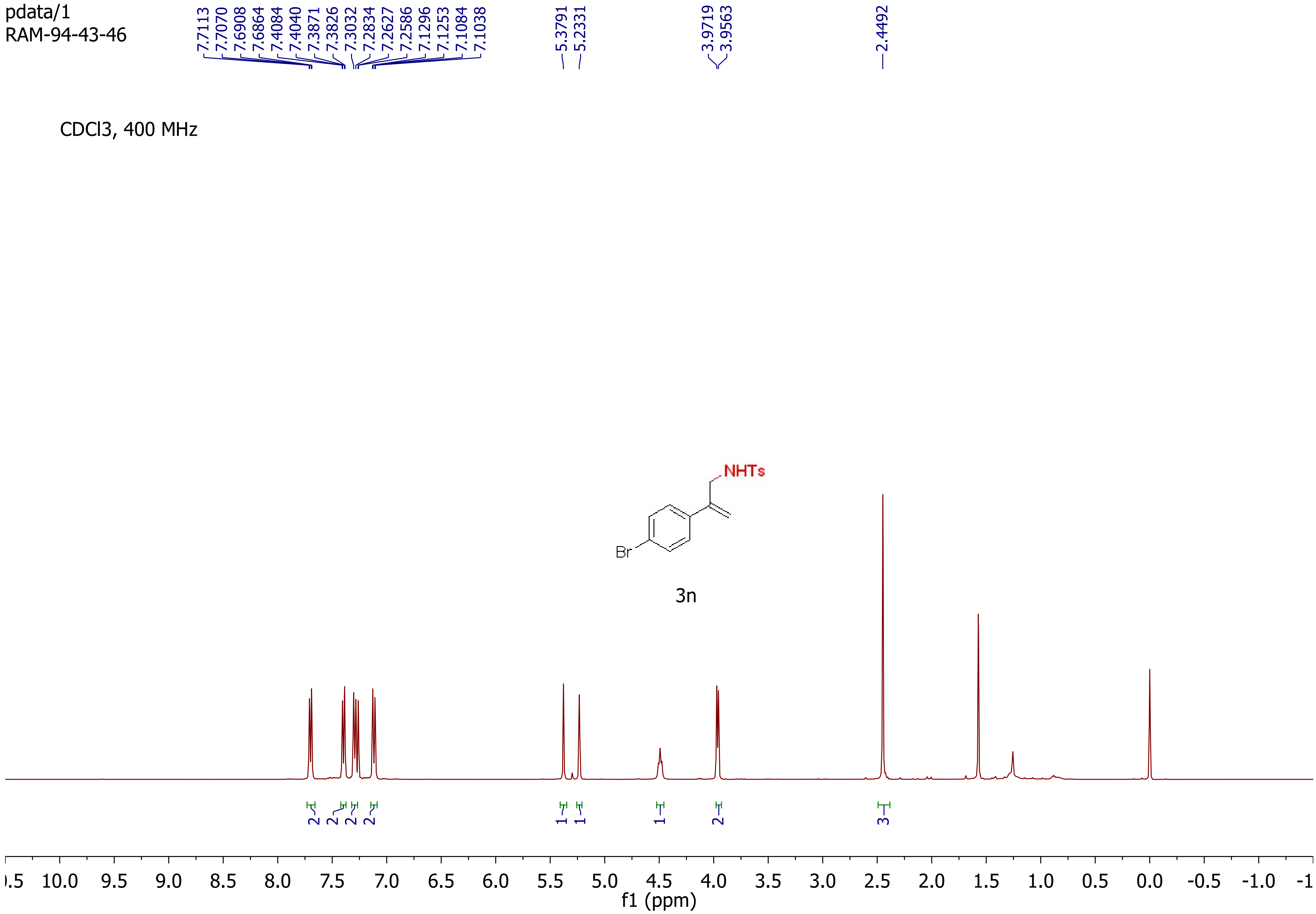
pdata/1
RAM-94-43-46
CDCl3, 400 MHz
7.7113
7.7070
7.6908
7.6864
7.4084
7.4040
7.3871
7.3826
7.3032
7.2834
7.2627
7.2586
7.1296
7.1253
7.1084
7.1038
5.3791
5.2331
3.9719
3.9563
2.4492
NHTs
Br
f1 (ppm)

3n

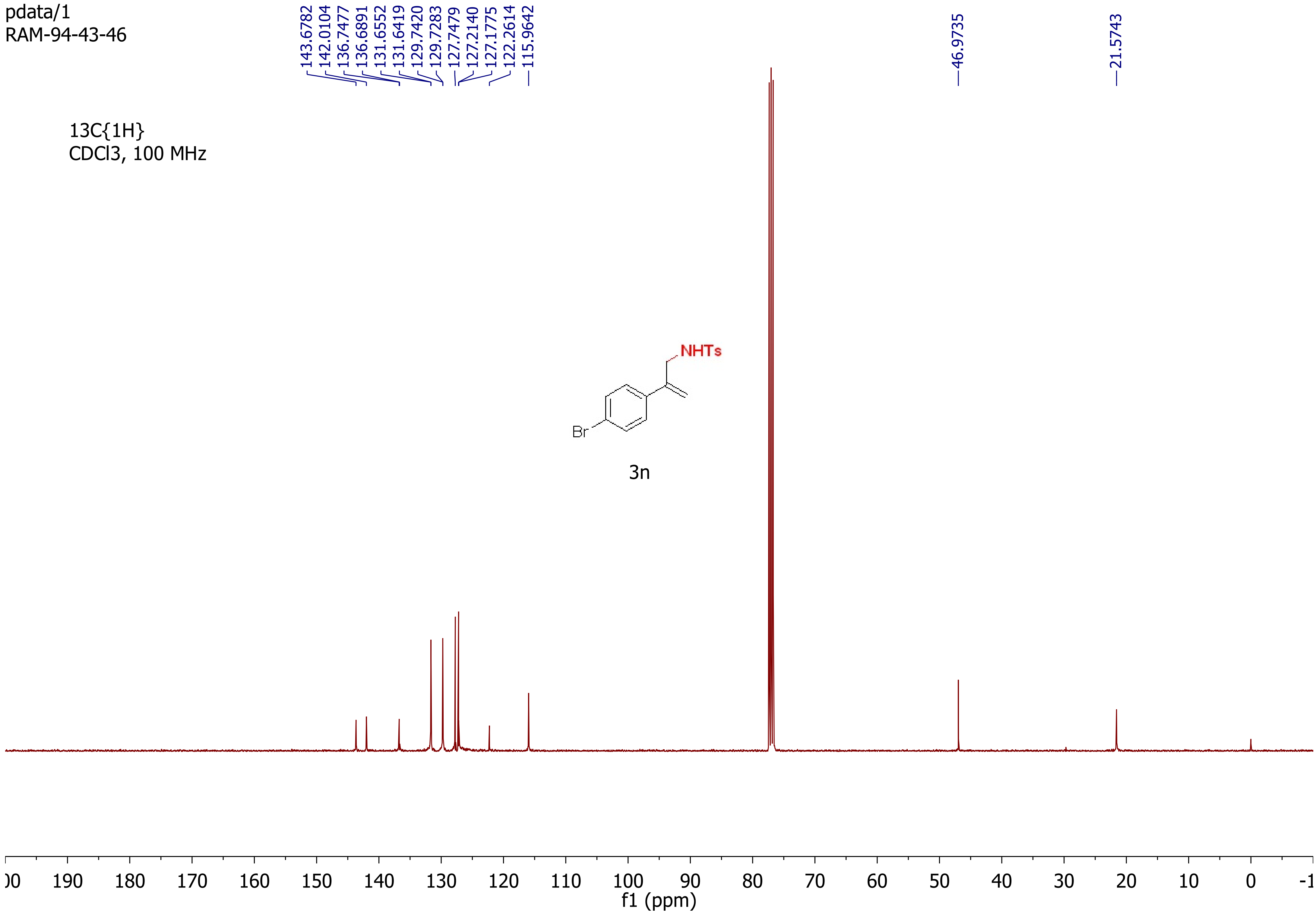
pdata/1
RAM-94-43-46
143.6782
142.0104
136.7477
136.6891
131.6552
131.6419
129.7420
129.7283
127.7479
127.2140
127.1775
122.2614
115.9642
46.9735
21.5743
13C{1H}
CDCl3, 100 MHz
NHTs
Br
3n
190
180
170
160
150
140
130
120
110
100
90
80
70
60
50
40
30
20
10
0
f1 (ppm)

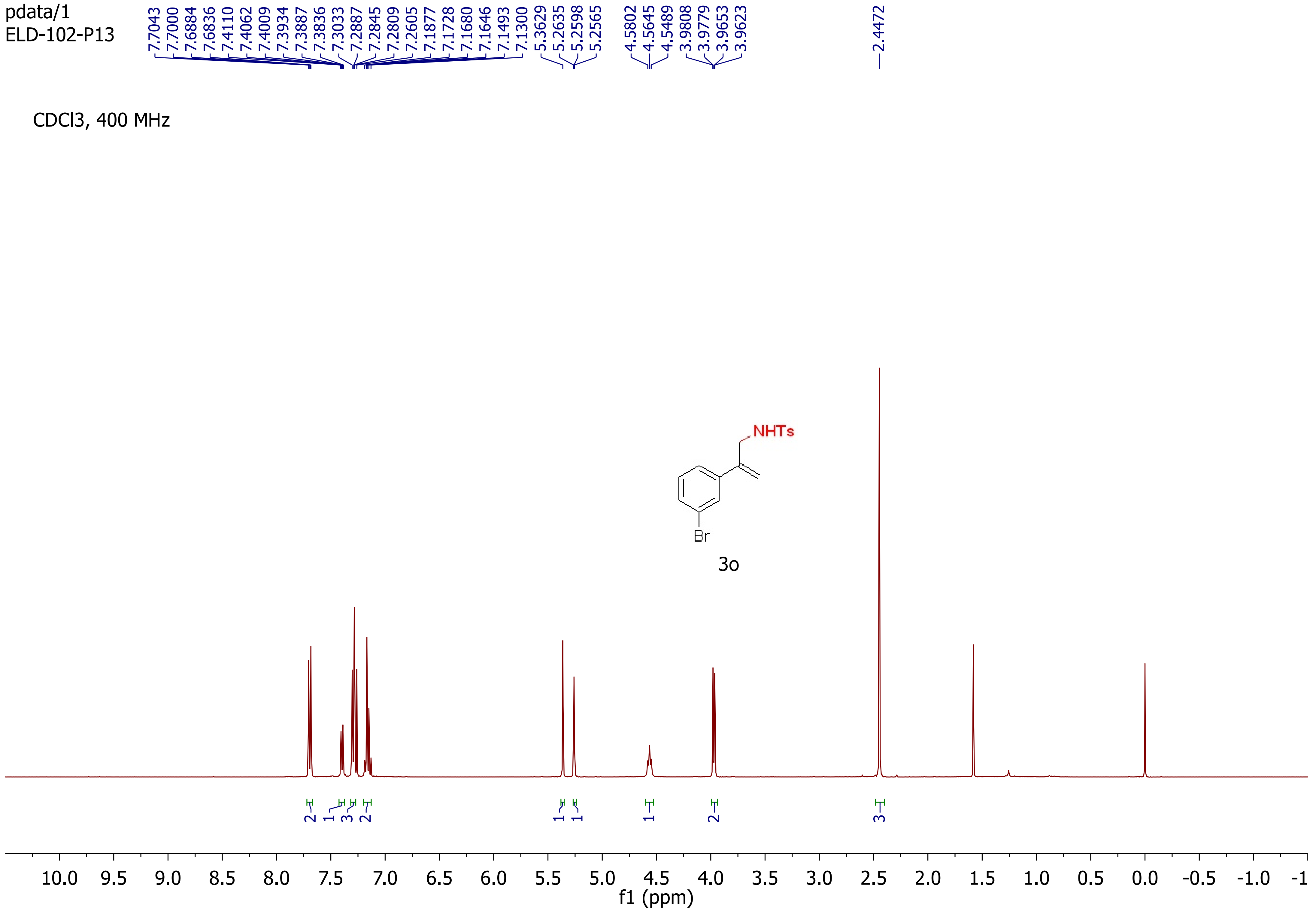
pdata/1
ELD-102-P13
7.7043
7.7000
7.6884
7.6836
7.4110
7.4062
7.4009
7.3934
7.3887
7.3836
7.3033
7.2887
7.2845
7.2809
7.2605
7.1877
7.1728
7.1680
7.1646
7.1493
7.1300
5.3629
5.2635
5.2598
5.2565
4.5802
4.5645
4.5489
3.9808
3.9779
3.9653
3.9623
2.4472
CDCl3, 400 MHz
NHTs
Br
3o
2
1
3
2
1
1
1
2
3
10.0 9.5 9.0 8.5 8.0 7.5 7.0 6.5 6.0 5.5 5.0 4.5 4.0 3.5 3.0 2.5 2.0 1.5 1.0 0.5 0.0 -0.5 -1.0 -1
f1 (ppm)

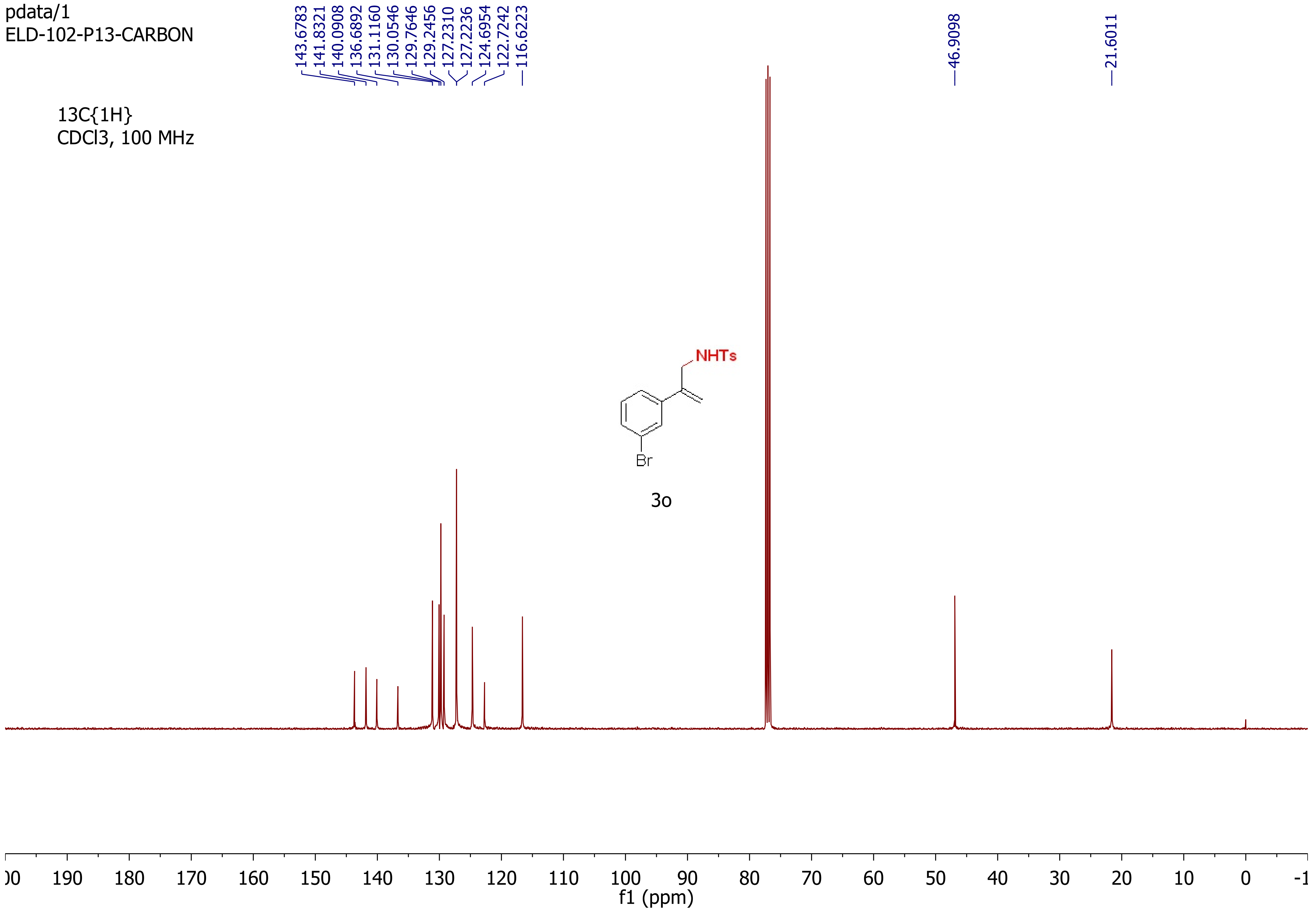
pdata/1
ELD-102-P13-CARBON
13C{1H}
CDCl3, 100 MHz
143.6783
141.8321
140.0908
136.6892
131.1160
130.0546
129.7646
129.2456
127.2310
127.2236
124.6954
122.7242
116.6223
46.9098
21.6011
NHTs
Br
3o
f1 (ppm)

pdata/1
RAM-102-49-50

CDCl3, 400 MHz

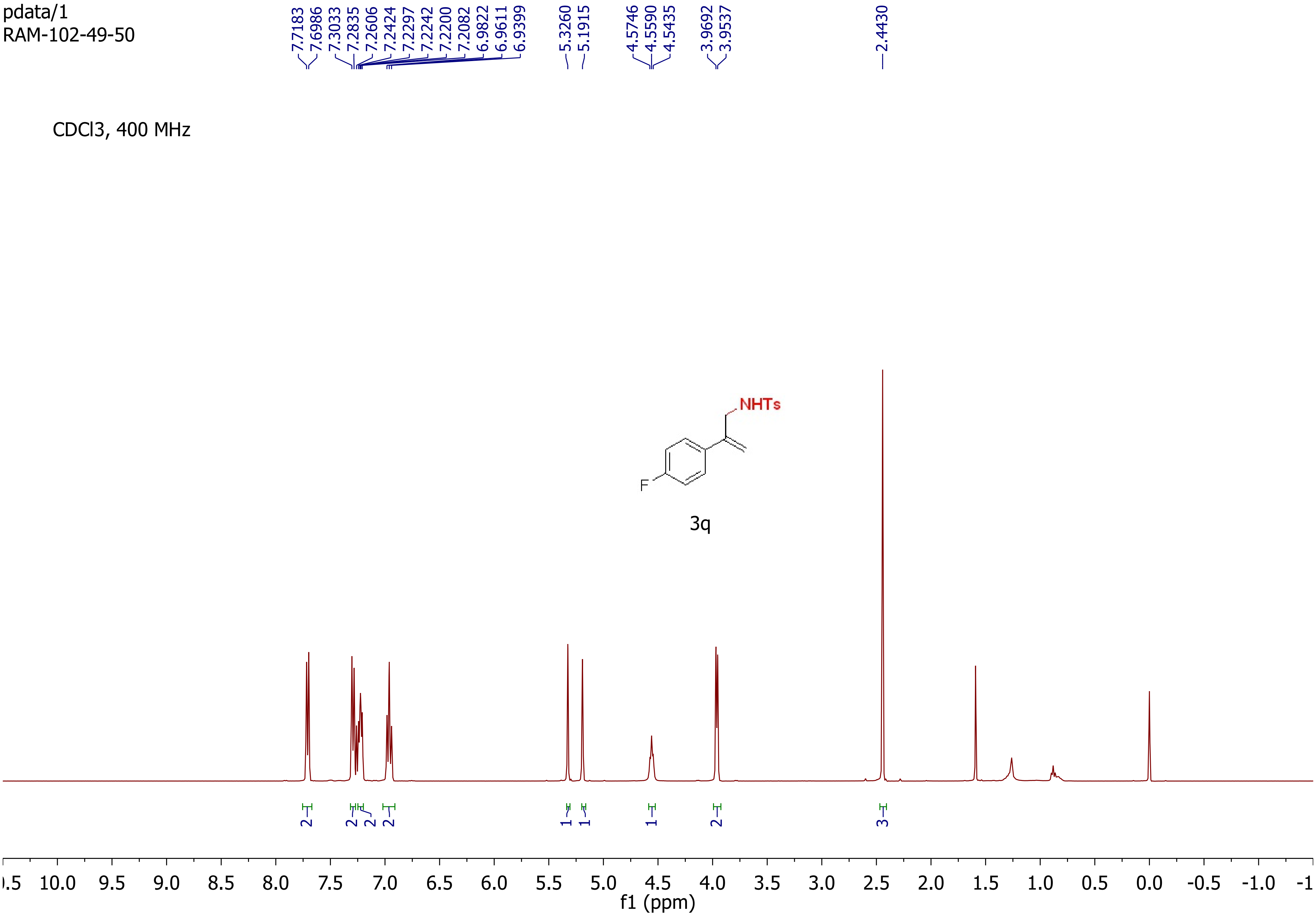

3q

pdata/1
RAM-102-49-50

13C{1H}
CDCl3, 100 MHz

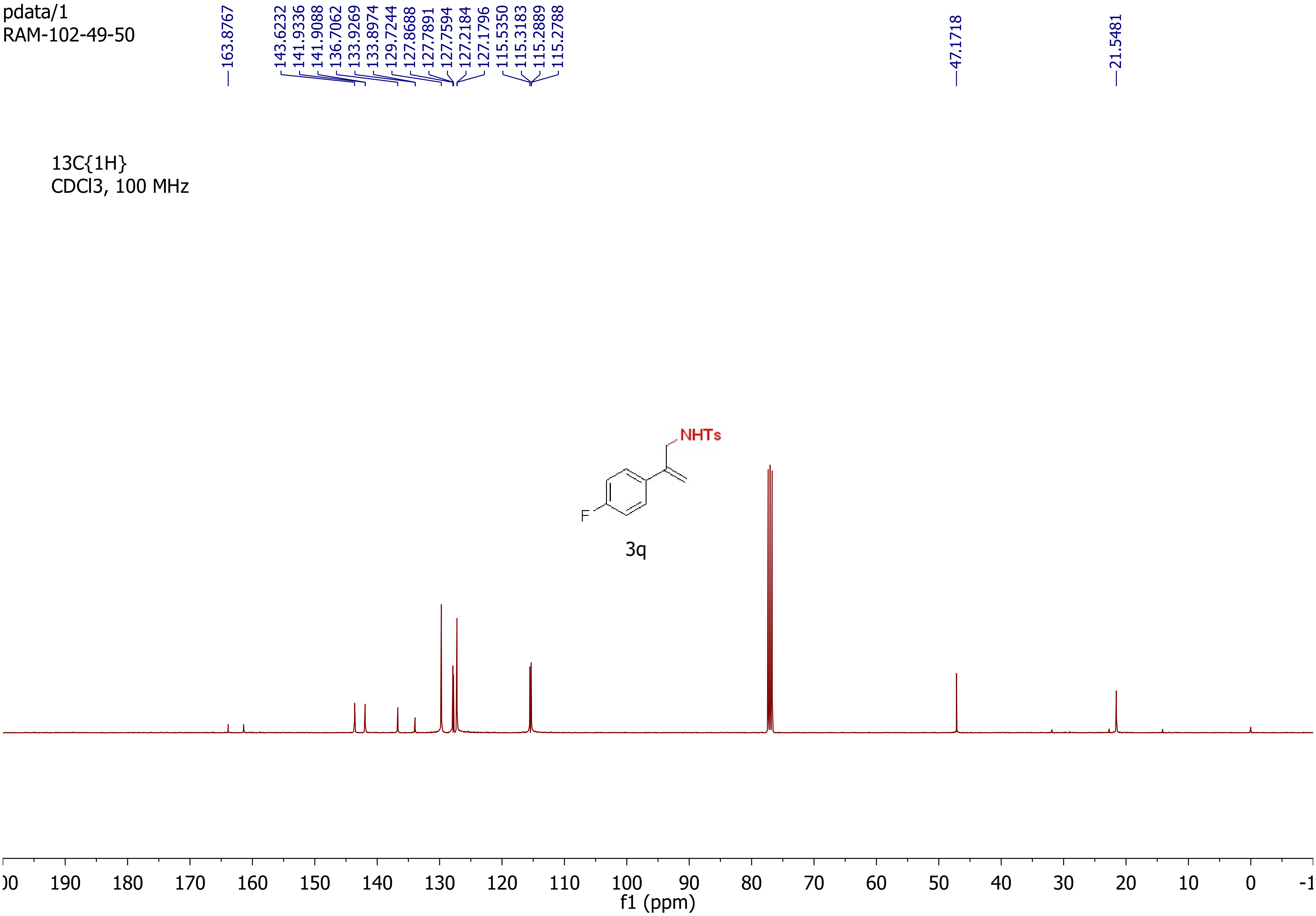

3q

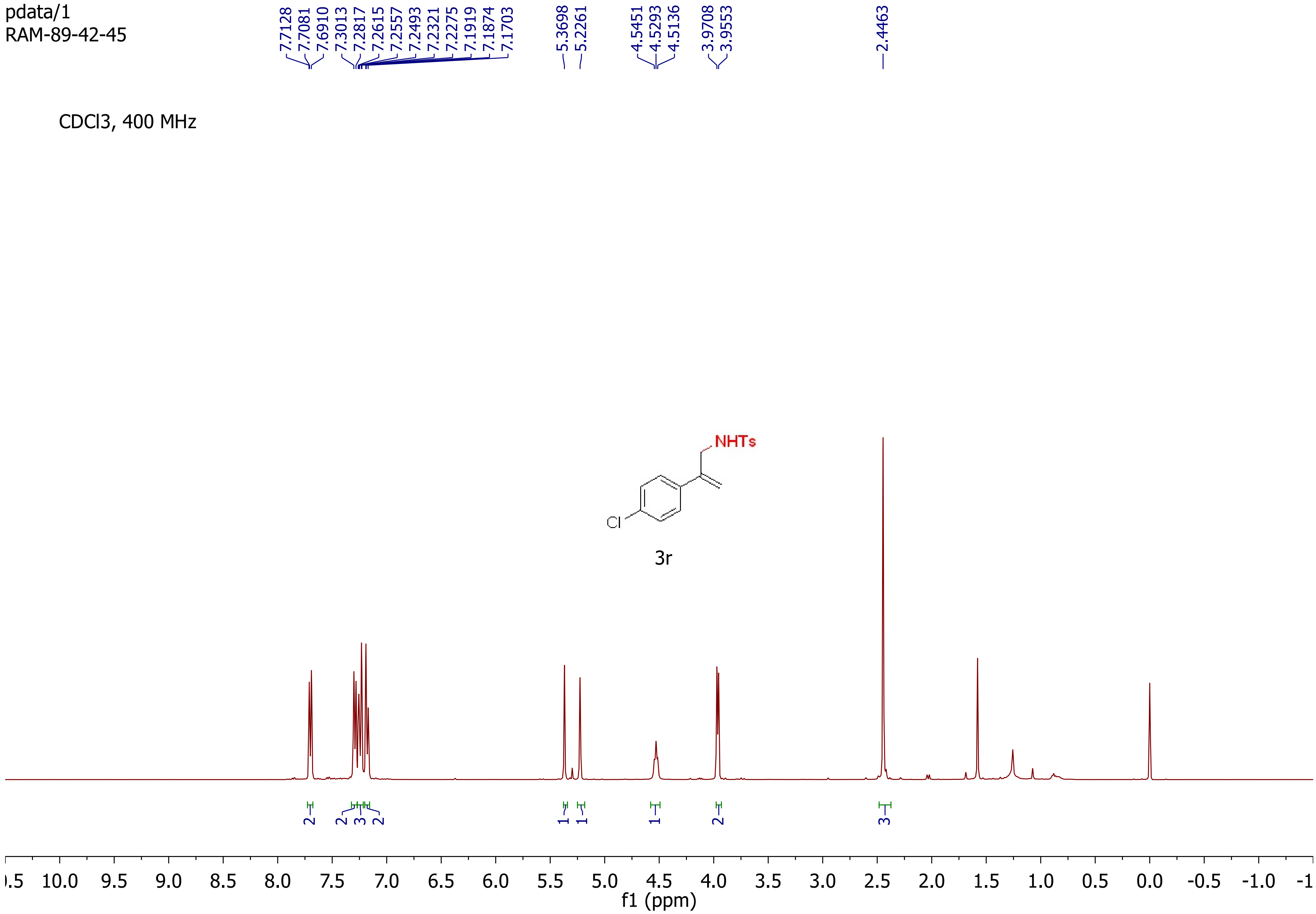
pdata/1
RAM-89-42-45
CDCl3, 400 MHz
7.7128
7.7081
7.6910
7.3013
7.2817
7.2615
7.2557
7.2493
7.2321
7.2275
7.1919
7.1874
7.1703
5.3698
5.2261
4.5451
4.5293
4.5136
3.9708
3.9553
2.4463
NHTs
Cl
3r
2
2
3
2
1
1
1
2
3
10.0
9.5
9.0
8.5
8.0
7.5
7.0
6.5
6.0
5.5
5.0
4.5
4.0
3.5
3.0
2.5
2.0
1.5
1.0
0.5
0.0
-0.5
-1.0
f1 (ppm)

pdata/1
RAM-89-42-45

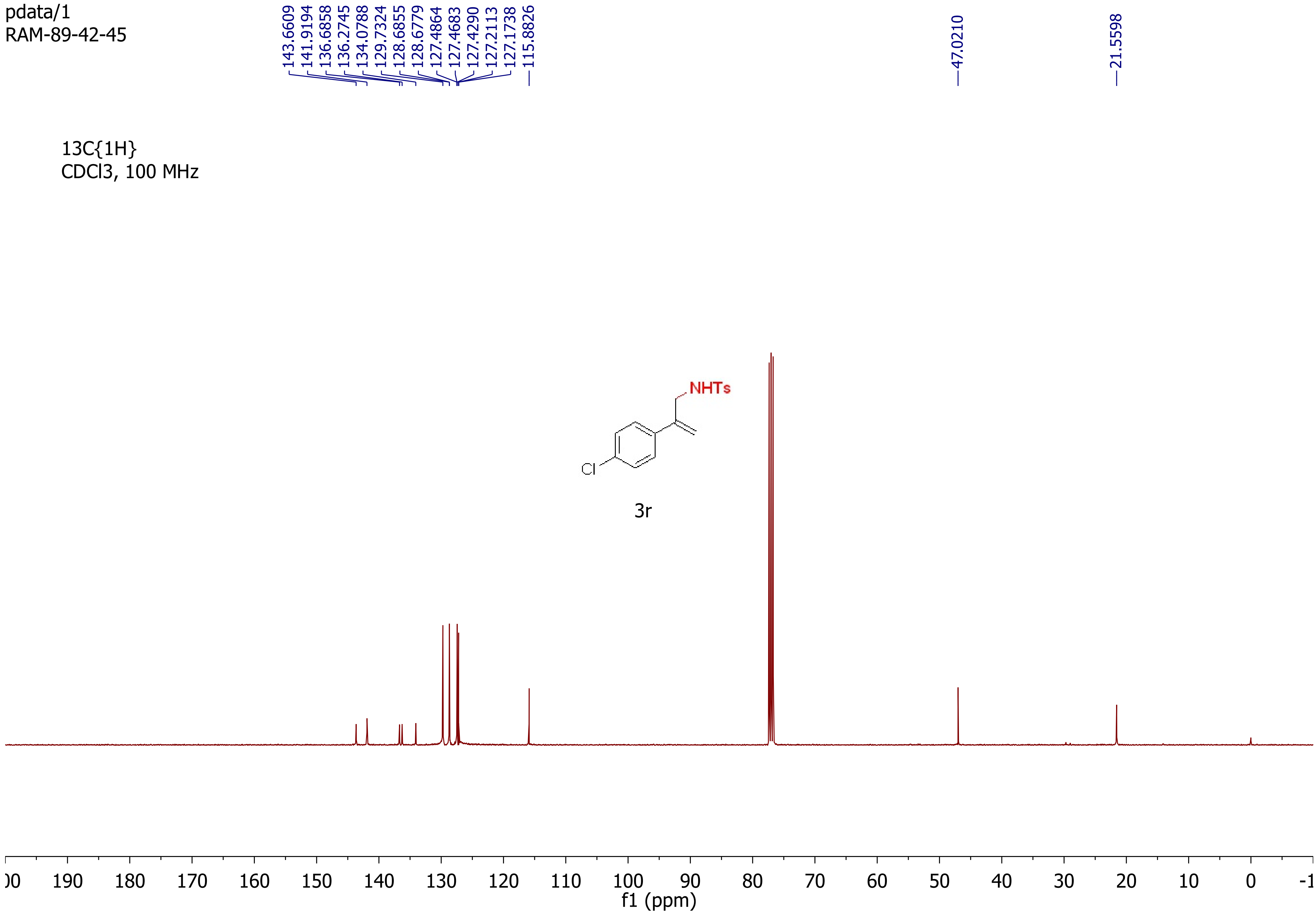

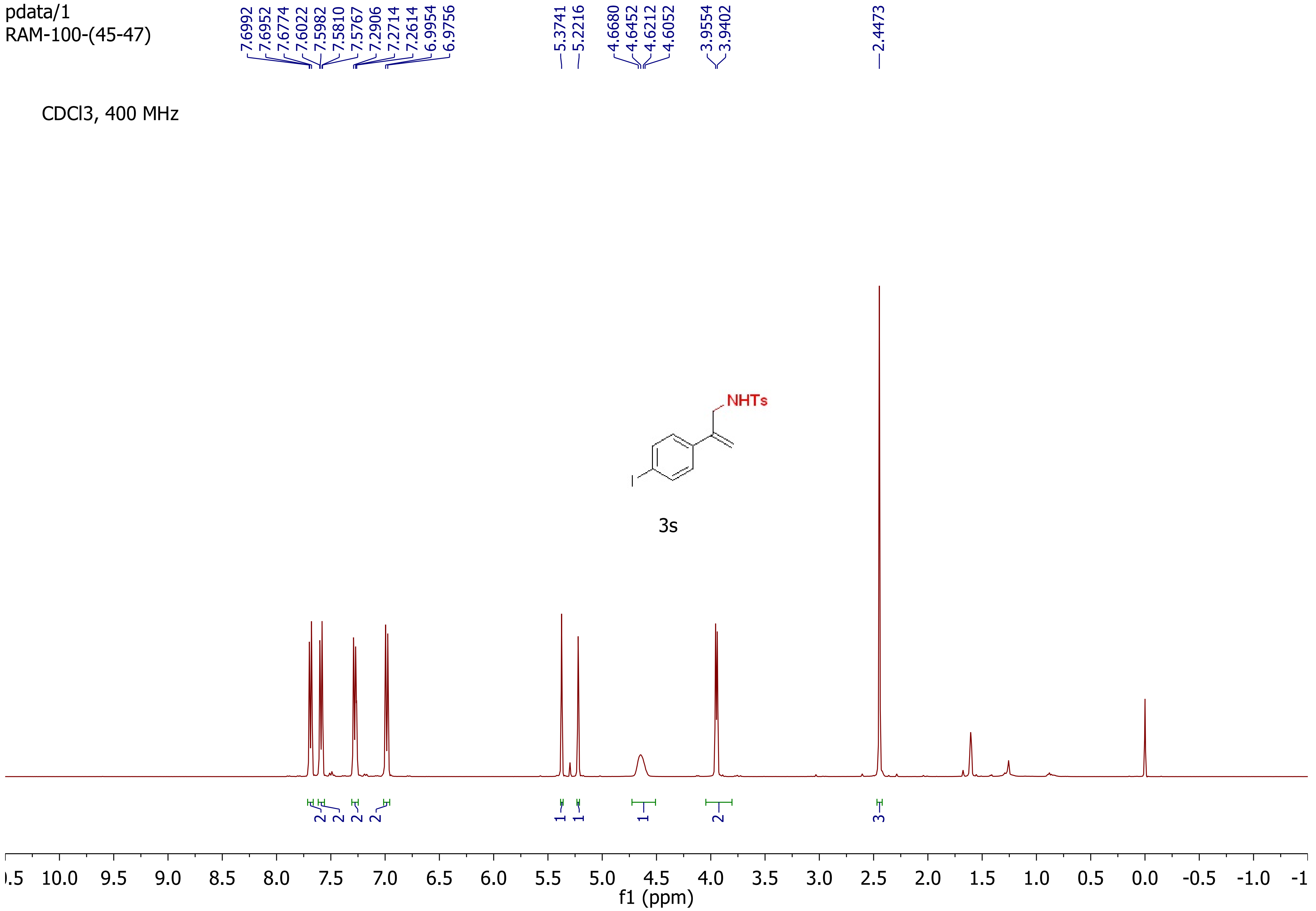

pdata/1
RAM-100-(45-47)
7.6992
7.6952
7.6774
7.6022
7.5982
7.5810
7.5767
7.2906
7.2714
7.2614
6.9954
6.9756
5.3741
5.2216
4.6680
4.6452
4.6212
4.6052
3.9554
3.9402
2.4473
CDCl3, 400 MHz
NHTs
I
3s
2
2
2
2
1
1
1
2
3
f1 (ppm)

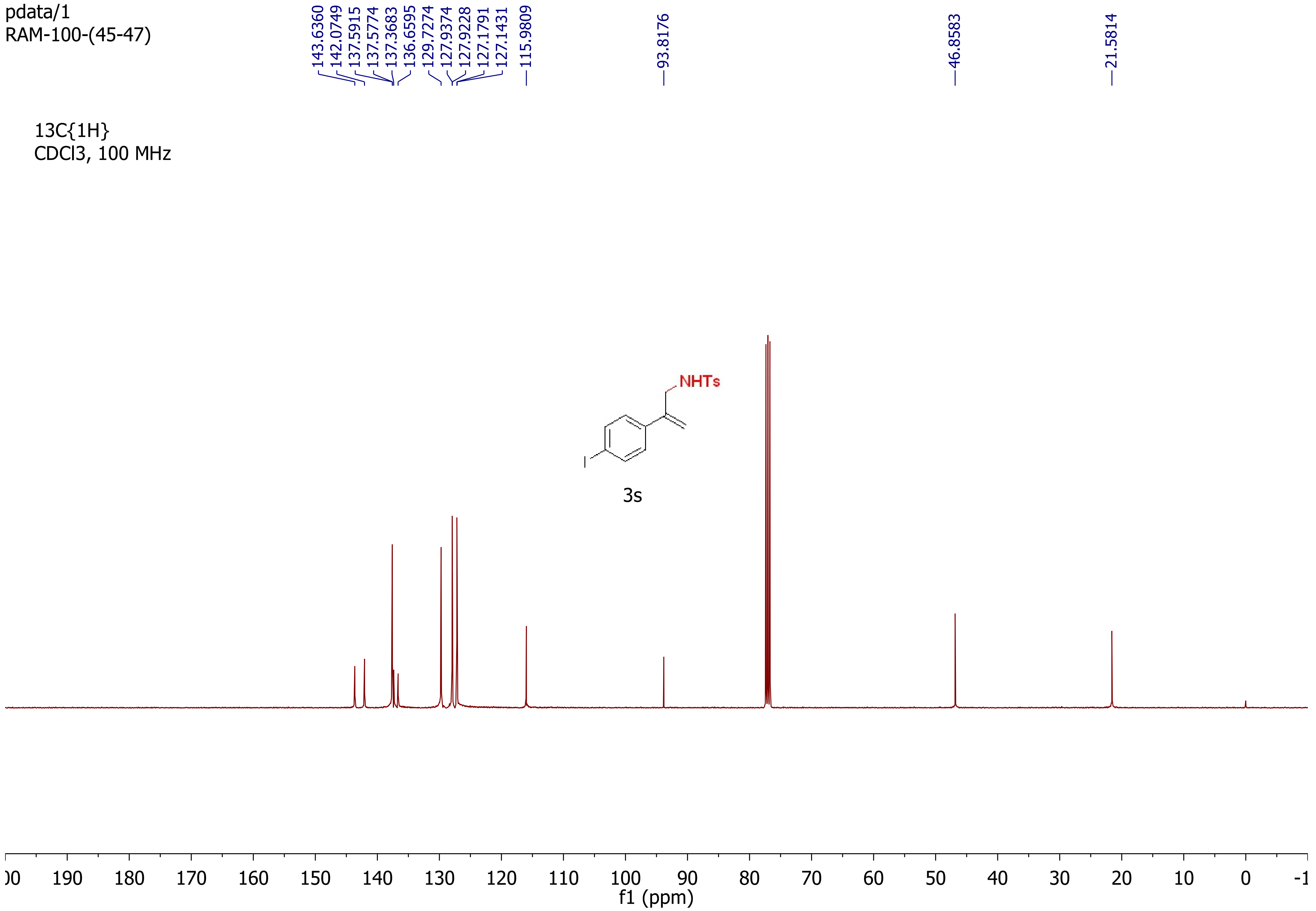
pdata/1
RAM-100-(45-47)
143.6360
142.0749
137.5915
137.5774
137.3683
136.6595
129.7274
127.9374
127.9228
127.1791
127.1431
115.9809
93.8176
46.8583
21.5814
13C{1H}
CDCl3, 100 MHz
NHTs
I
3s
190
180
170
160
150
140
130
120
110
100
90
80
70
60
50
40
30
20
10
0
f1 (ppm)

pdata/1
RAM-101-52

CDCl3, 400 MHz

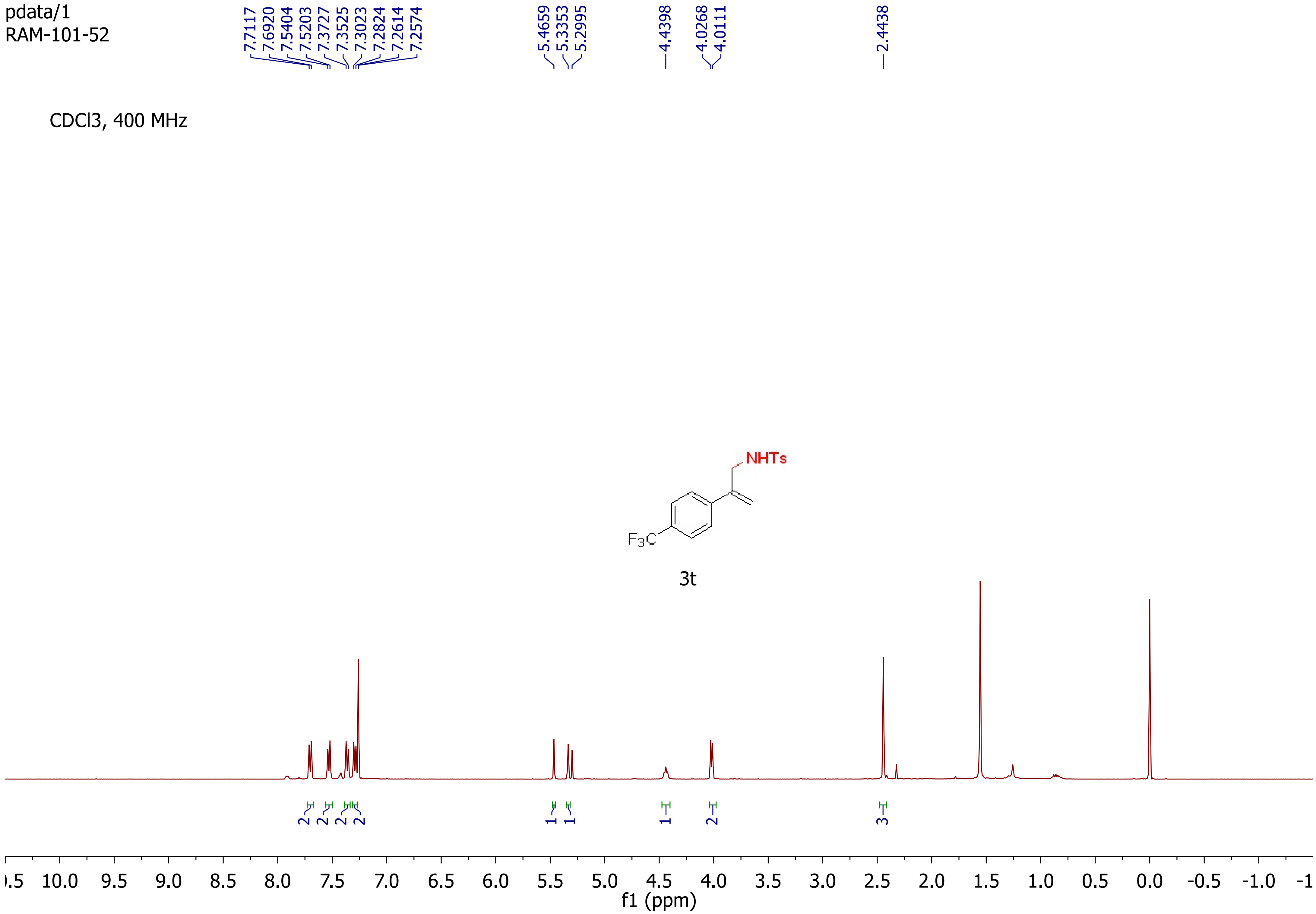

3t

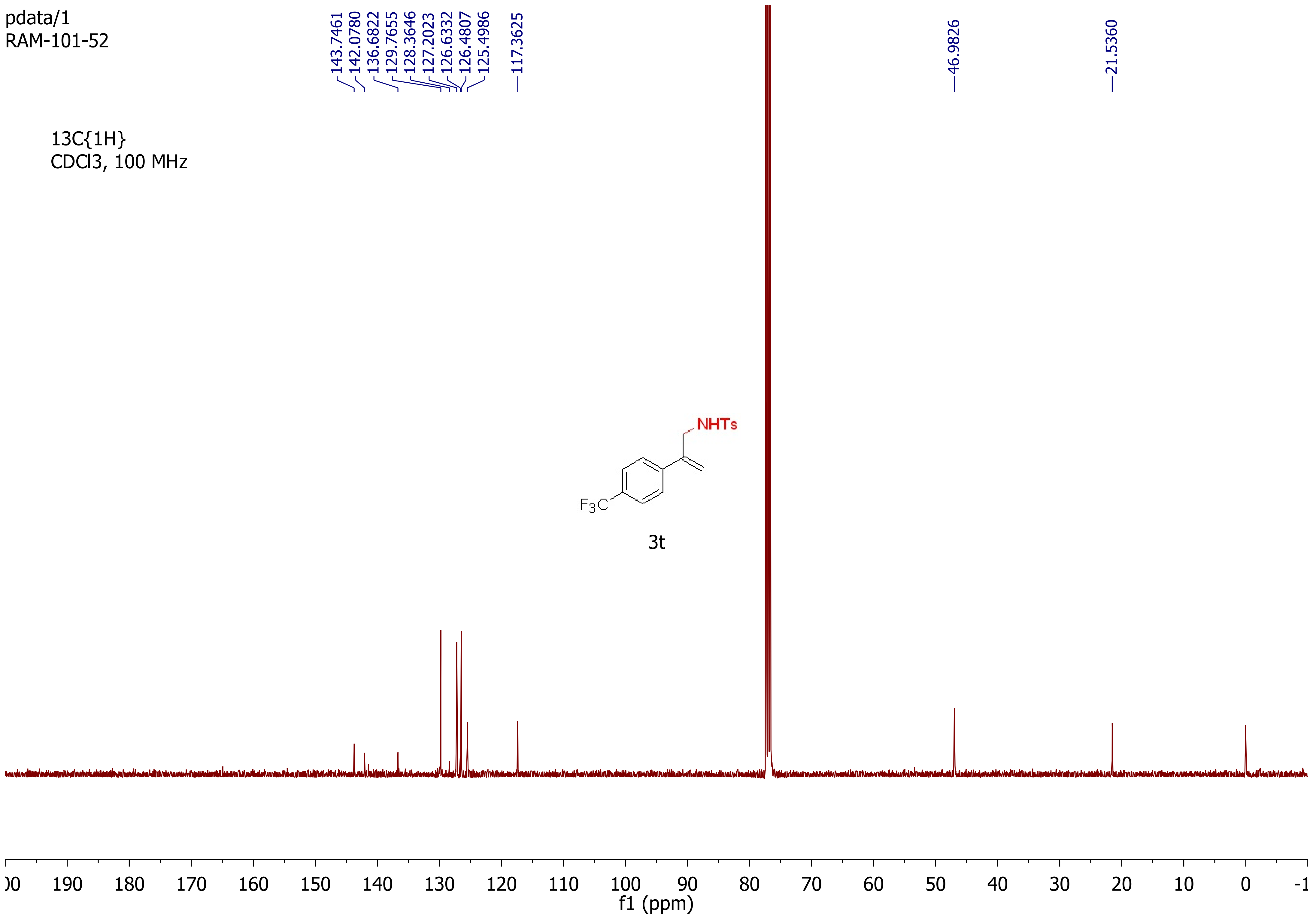
pdata/1
RAM-101-52
13C{1H}
CDCl3, 100 MHz
143.7461
142.0780
136.6822
129.7655
128.3646
127.2023
126.6332
126.4807
125.4986
117.3625
46.9826
21.5360
NHTs
F3C
3t
f1 (ppm)

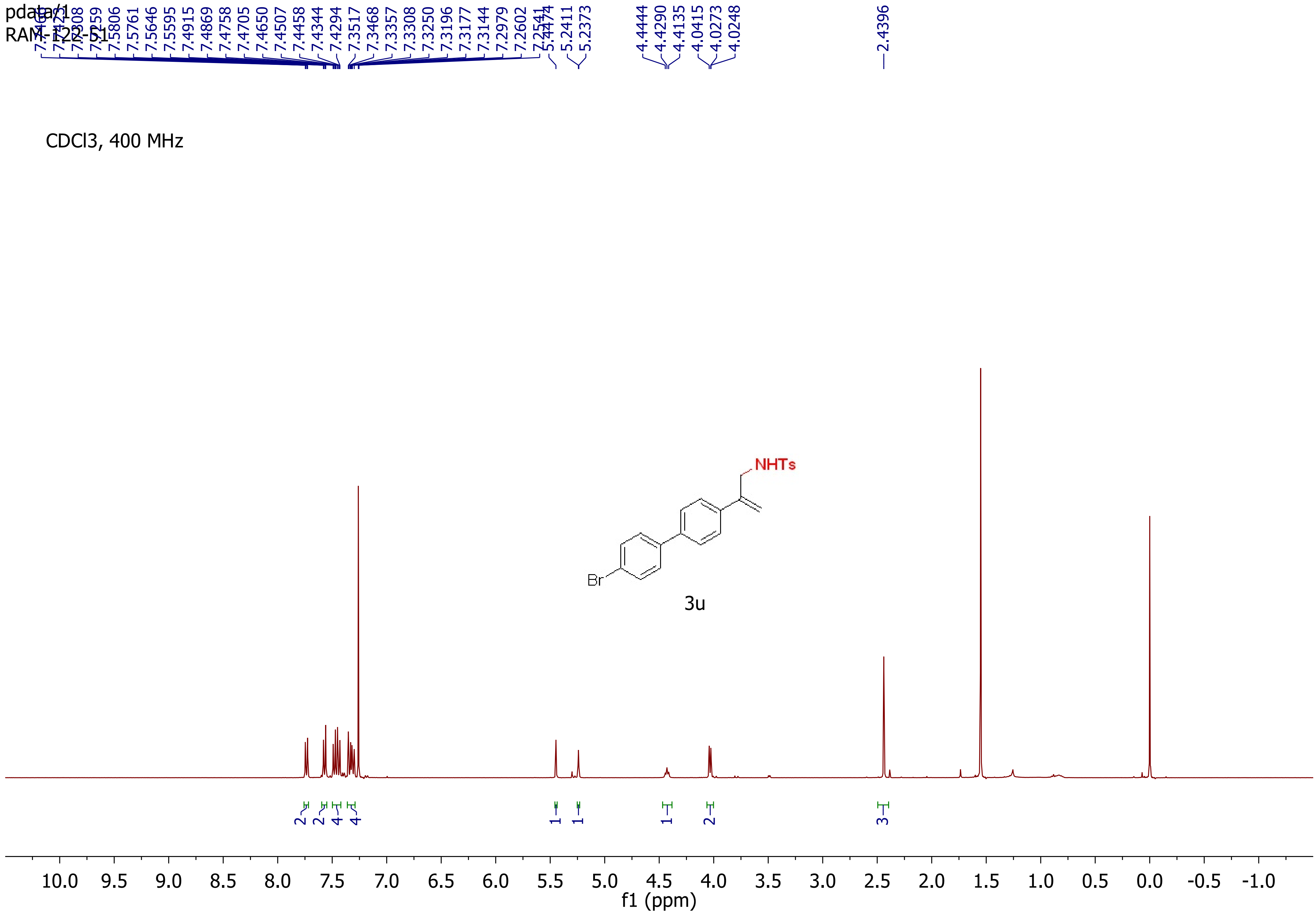
pdata/1
RAM-122-51
7.7308
7.7259
7.5806
7.5761
7.5646
7.5595
7.4915
7.4869
7.4758
7.4705
7.4650
7.4507
7.4458
7.4344
7.4294
7.3517
7.3468
7.3357
7.3308
7.3250
7.3196
7.3177
7.3144
7.2979
7.2602
7.2541
5.4474
5.2411
5.2373
4.4444
4.4290
4.4135
4.0415
4.0273
4.0248
2.4396
CDCl3, 400 MHz
NHTs
Br
3u
2
2
4
4
1
1
1
2
3
10.0
9.5
9.0
8.5
8.0
7.5
7.0
6.5
6.0
5.5
5.0
4.5
4.0
3.5
3.0
2.5
2.0
1.5
1.0
0.5
0.0
-0.5
-1.0
f1 (ppm)

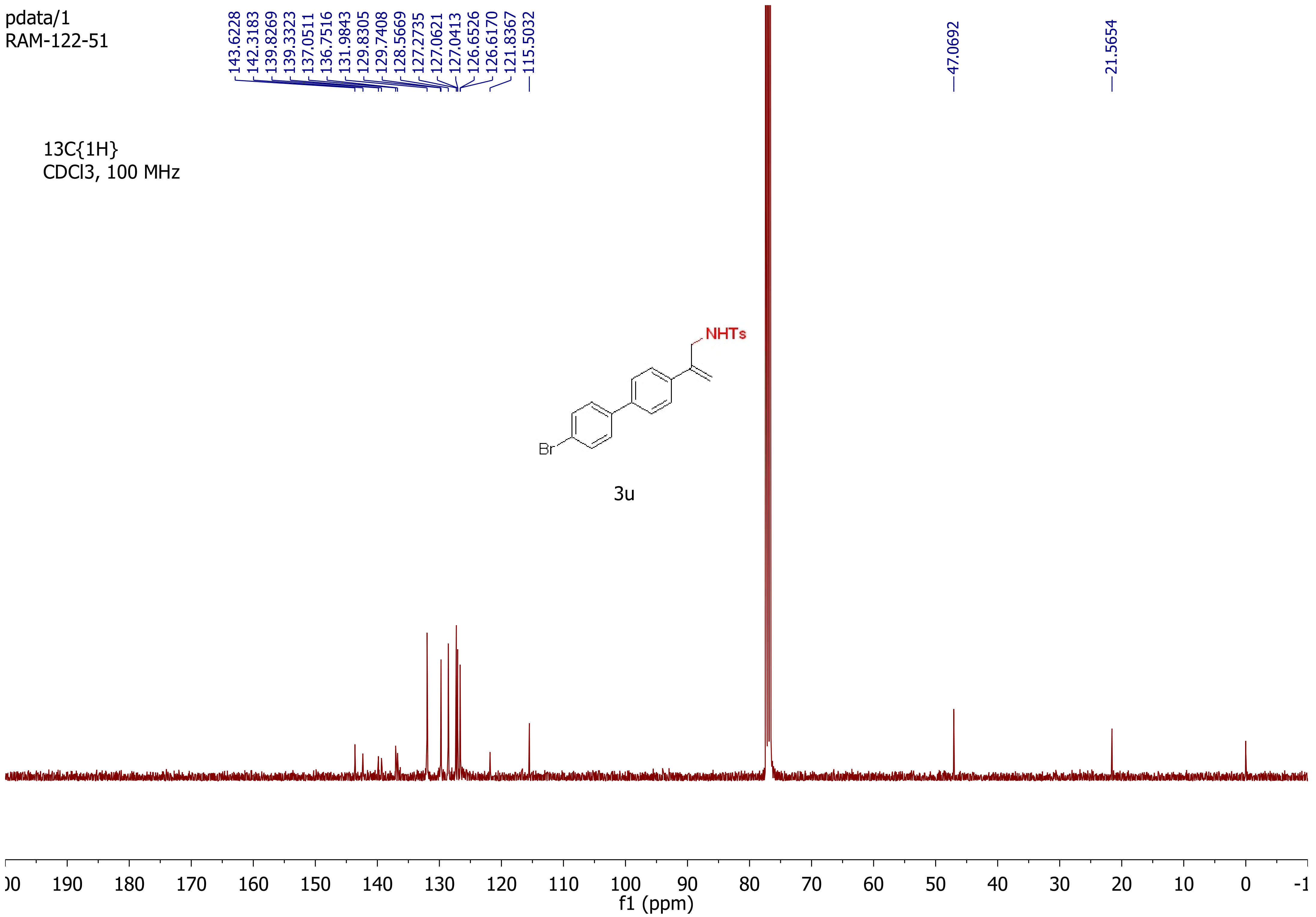
pdata/1
RAM-122-51
13C{1H}
CDCl3, 100 MHz
143.6228
142.3183
139.8269
139.3323
137.0511
136.7516
131.9843
129.8305
129.7408
128.5669
127.2735
127.0621
127.0413
126.6526
126.6170
121.8367
115.5032
47.0692
21.5654
NHTs
Br
3u
f1 (ppm)

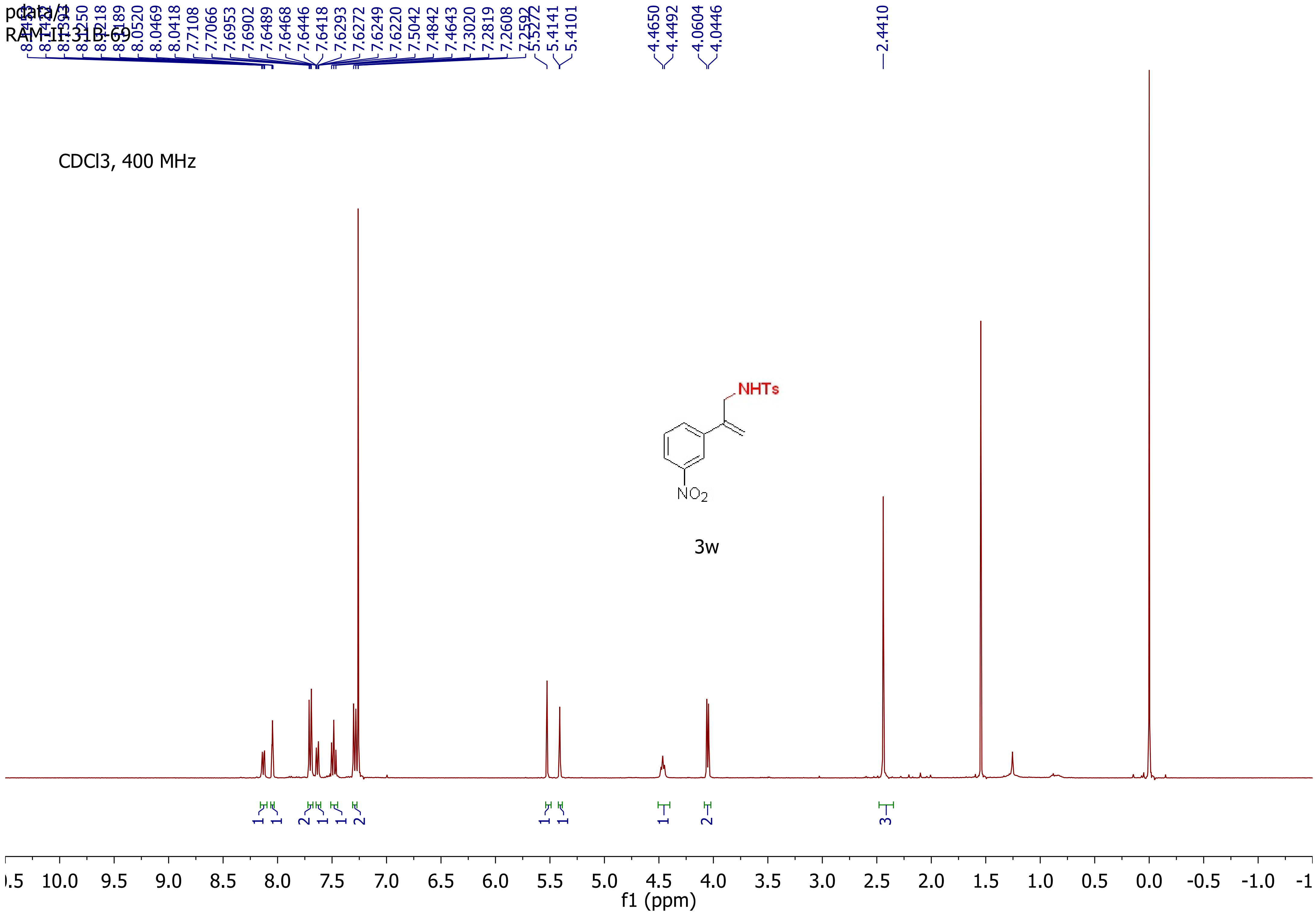

pdata/1
RAM-II-31B-69
8.0520
8.0469
8.0418
7.7108
7.7066
7.6953
7.6902
7.6489
7.6468
7.6446
7.6418
7.6293
7.6272
7.6249
7.6220
7.5042
7.4842
7.4643
7.3020
7.2819
7.2608
7.2592
5.5272
5.4141
5.4101
4.4650
4.4492
4.0604
4.0446
2.4410
CDCl3, 400 MHz
NHTs
NO2
3w
1
1
2
1
1
2
1
1
1
2
3
f1 (ppm)

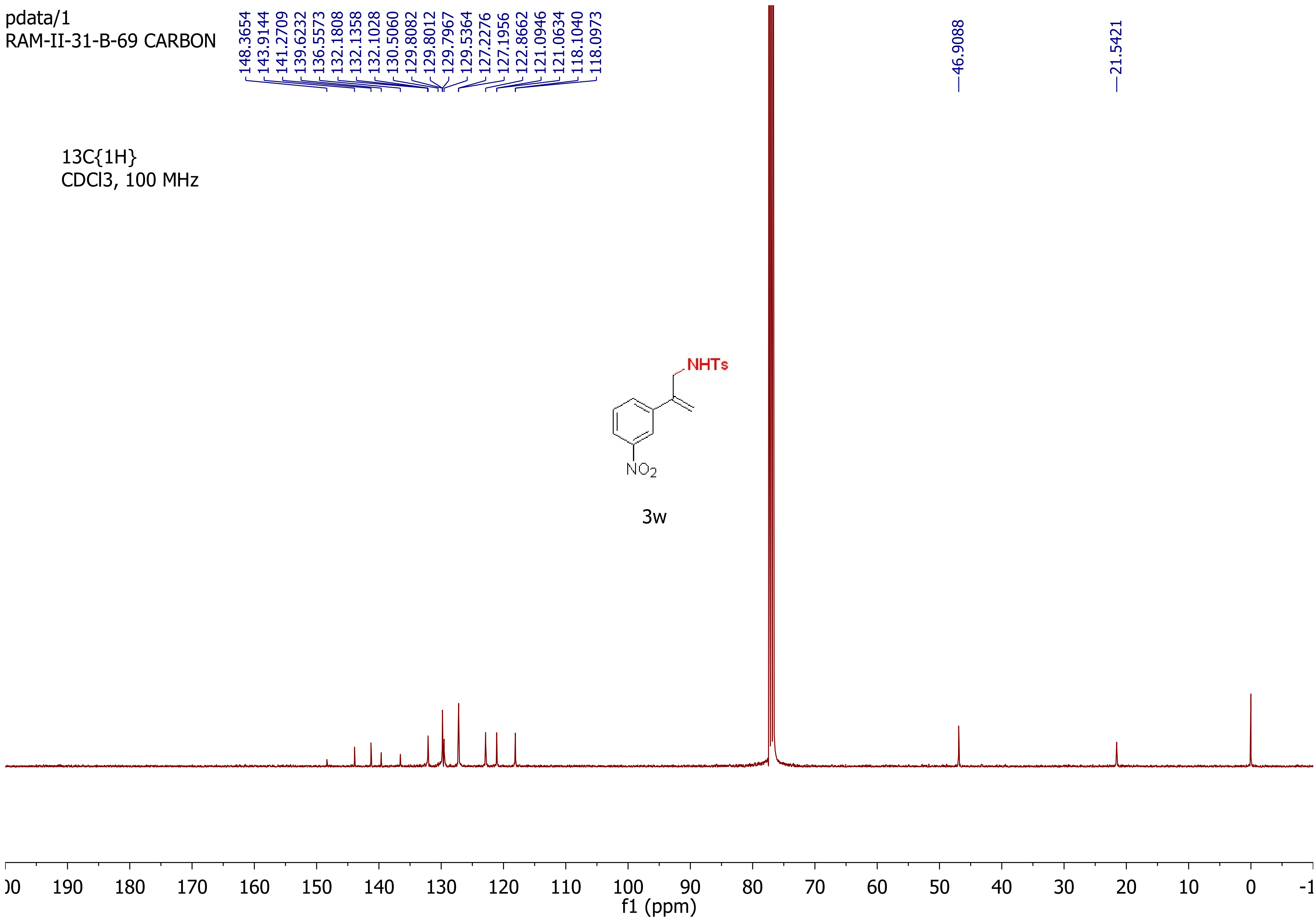

pdata/1
RAM-II-31-B-69 CARBON
148.3654
143.9144
141.2709
139.6232
136.5573
132.1808
132.1358
132.1028
130.5060
129.8082
129.8012
129.7967
129.5364
127.2276
127.1956
122.8662
121.0946
121.0634
118.1040
118.0973
46.9088
21.5421
13C{1H}
CDCl3, 100 MHz
NHTs
NO2
3w
f1 (ppm)

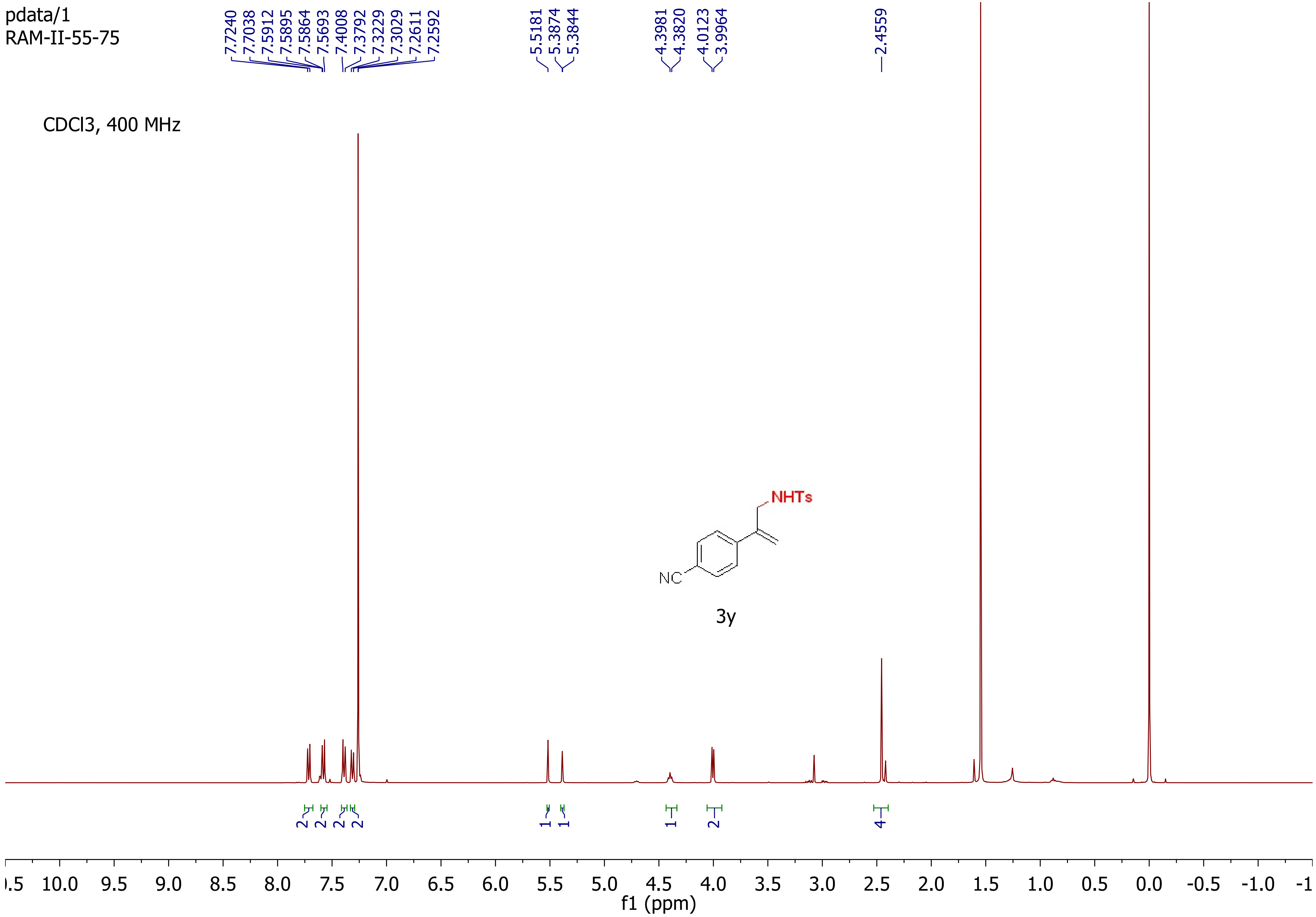
pdata/1
RAM-II-55-75
CDCl3, 400 MHz
7.7240
7.7038
7.5912
7.5895
7.5864
7.5693
7.4008
7.3792
7.3229
7.3029
7.2611
7.2592
5.5181
5.3874
5.3844
4.3981
4.3820
4.0123
3.9964
2.4559
NHTs
NC
3y
2
2
2
2
1
1
1
2
4
10.0
9.5
9.0
8.5
8.0
7.5
7.0
6.5
6.0
5.5
5.0
4.5
4.0
3.5
3.0
2.5
2.0
1.5
1.0
0.5
0.0
-0.5
-1.0
f1 (ppm)

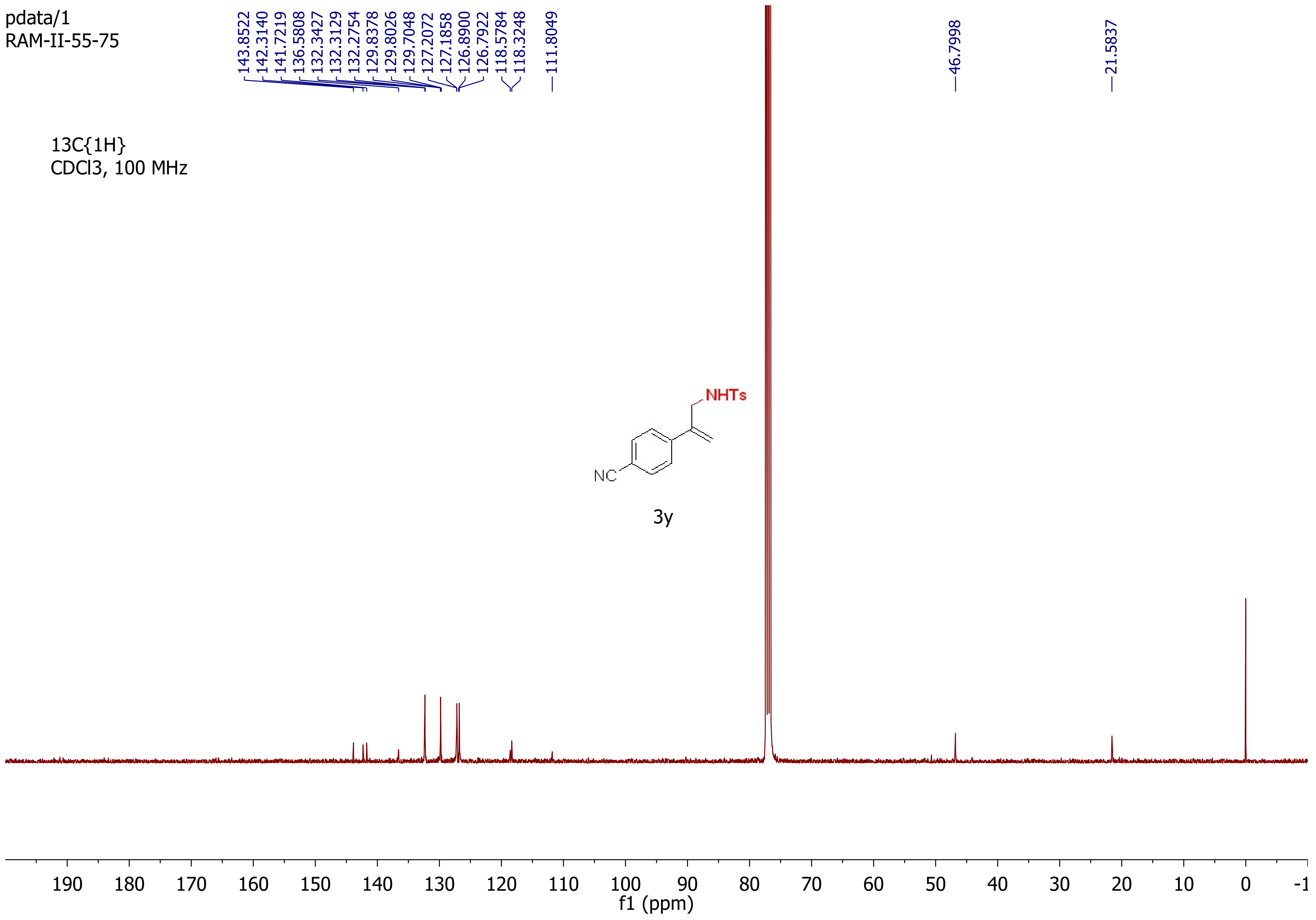

pdata/1
RAM-II-55-75
13C{1H}
CDCl3, 100 MHz
143.8522
142.3140
141.7219
136.5808
132.3427
132.3129
132.2754
129.8378
129.8026
129.7048
127.2072
127.1858
126.8900
126.7922
118.5784
118.3248
111.8049
46.7998
21.5837
NHTs
NC
3y
190
180
170
160
150
140
130
120
110
100
90
80
70
60
50
40
30
20
10
0
-1
f1 (ppm)

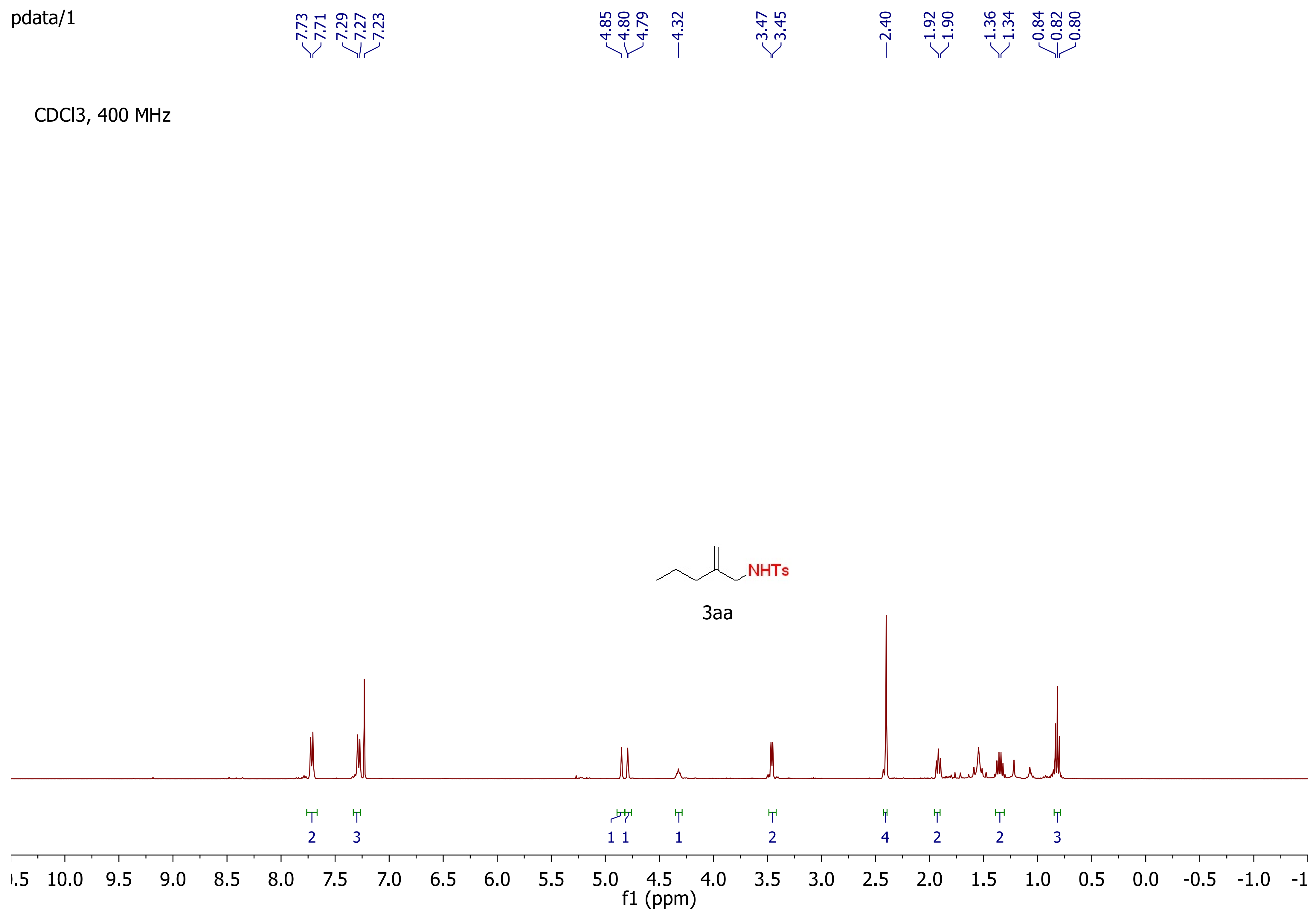
pdata/1
CDCl3, 400 MHz
7.73
7.71
7.29
7.27
7.23
4.85
4.80
4.79
4.32
3.47
3.45
2.40
1.92
1.90
1.36
1.34
0.84
0.82
0.80
NHTs
3aa
2
3
1
1
1
2
4
2
2
3
10.0
9.5
9.0
8.5
8.0
7.5
7.0
6.5
6.0
5.5
5.0
4.5
4.0
3.5
3.0
2.5
2.0
1.5
1.0
0.5
0.0
-0.5
-1.0
f1 (ppm)

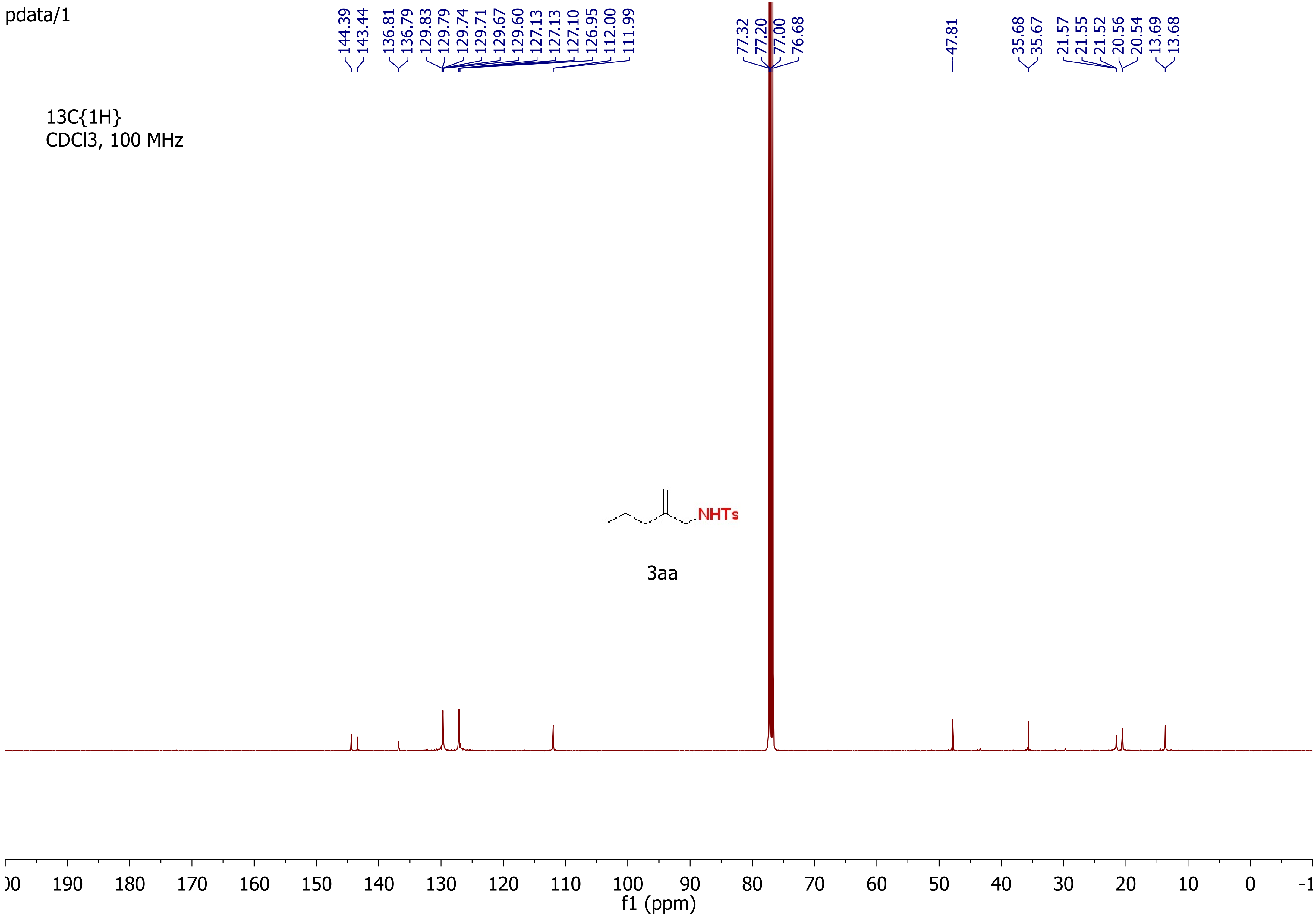
pdata/1
13C{1H}
CDCl3, 100 MHz
144.39
143.44
136.81
136.79
129.83
129.79
129.74
129.71
129.67
129.60
127.13
127.13
127.10
126.95
112.00
111.99
77.32
77.20
77.00
76.68
47.81
35.68
35.67
21.57
21.55
21.52
20.56
20.54
13.69
13.68
NHTs
3aa
f1 (ppm)

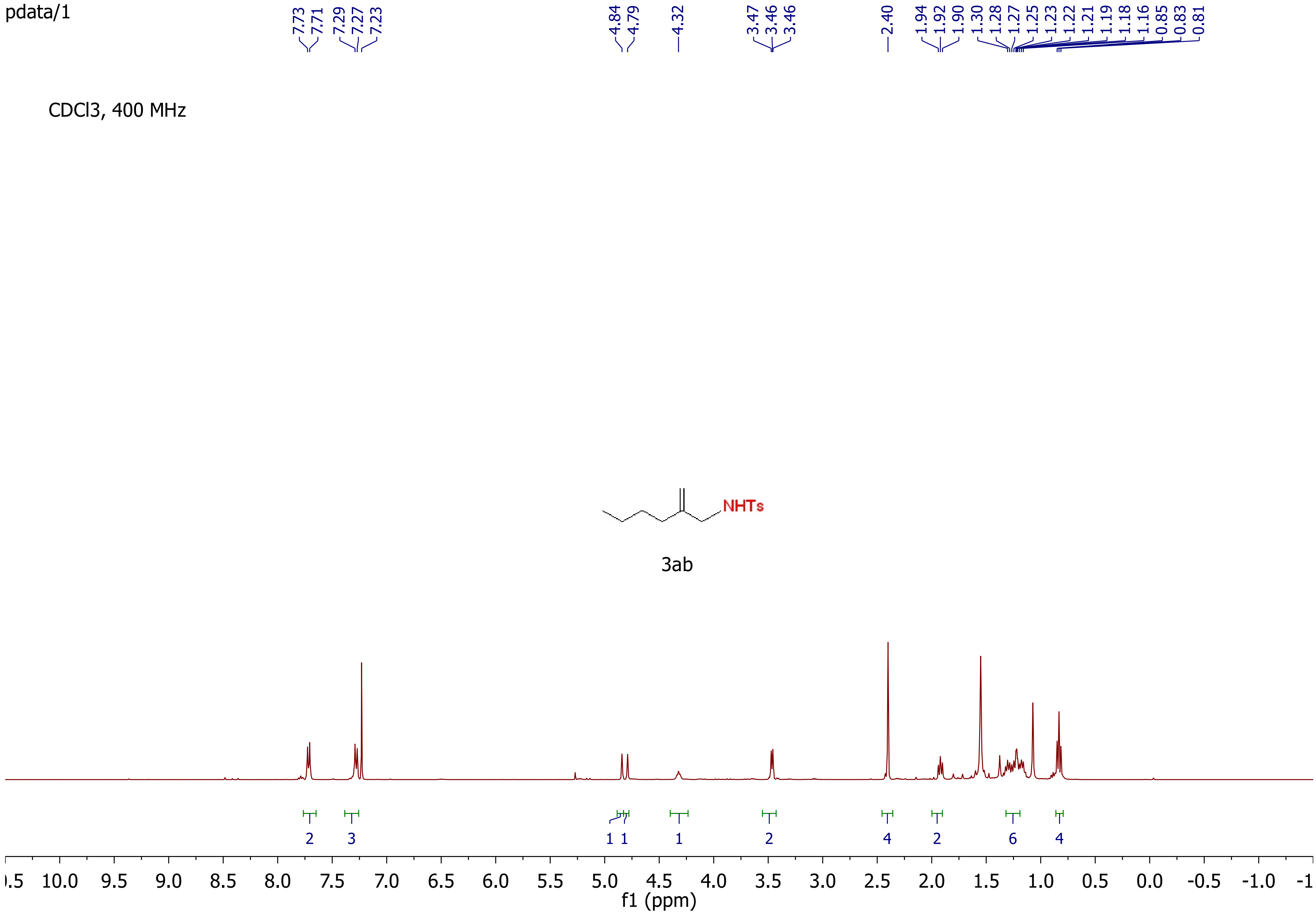
pdata/1
CDCl3, 400 MHz
7.73 7.71 7.29 7.27 7.23
4.84 4.79
4.32
3.47 3.46 3.46
2.40
1.94 1.92 1.90 1.30 1.28 1.27 1.25 1.23 1.22 1.21 1.19 1.18 1.16 0.85 0.83 0.81
NHTs
3ab
2 3 1 1 1 2 4 2 6 4
f1 (ppm)

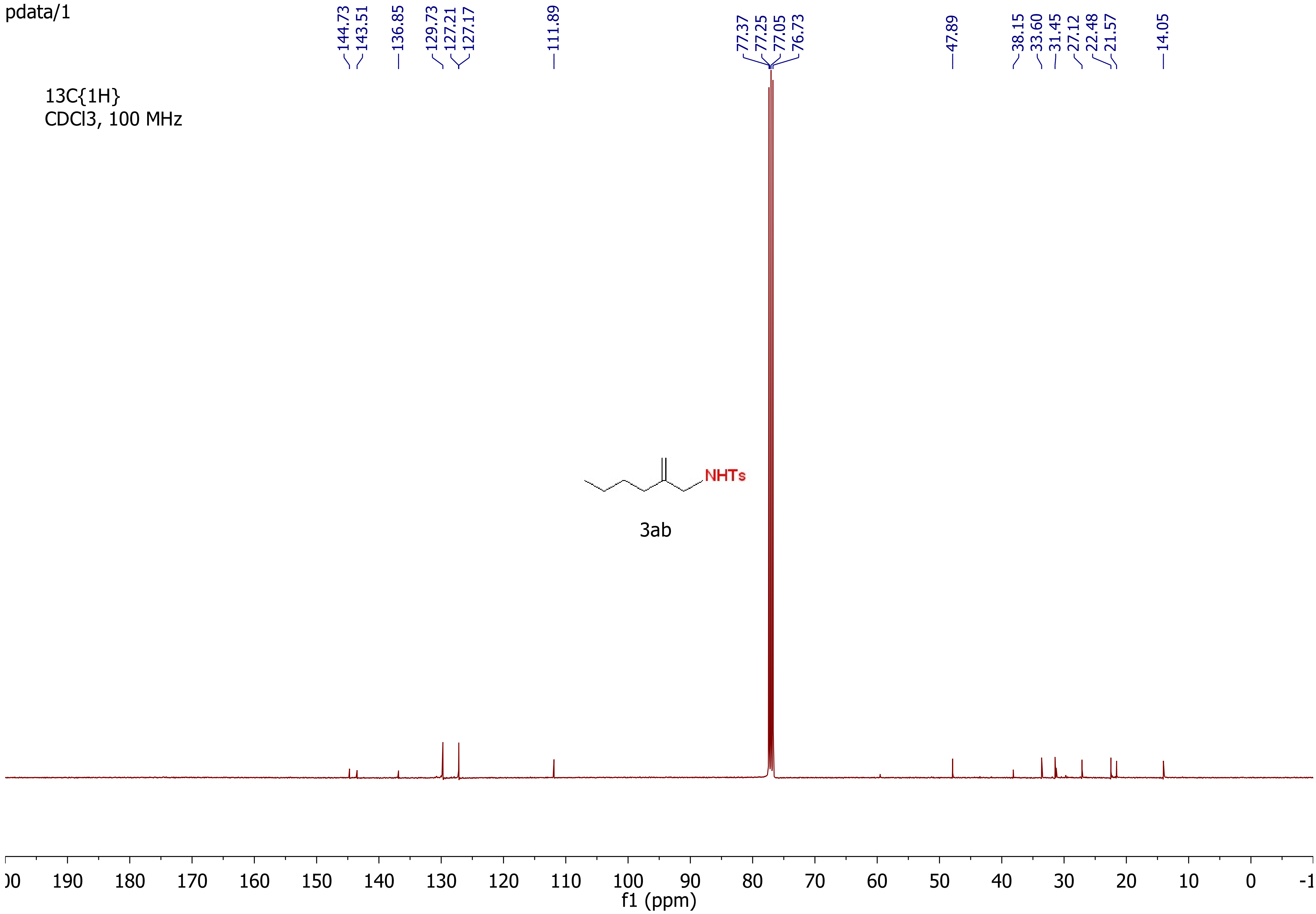

pdata/1
13C{1H}
CDCl3, 100 MHz
144.73
143.51
136.85
129.73
127.21
127.17
111.89
77.37
77.25
77.05
76.73
47.89
38.15
33.60
31.45
27.12
22.48
21.57
14.05
NHTs
3ab
190
180
170
160
150
140
130
120
110
100
90
80
70
60
50
40
30
20
10
0
f1 (ppm)

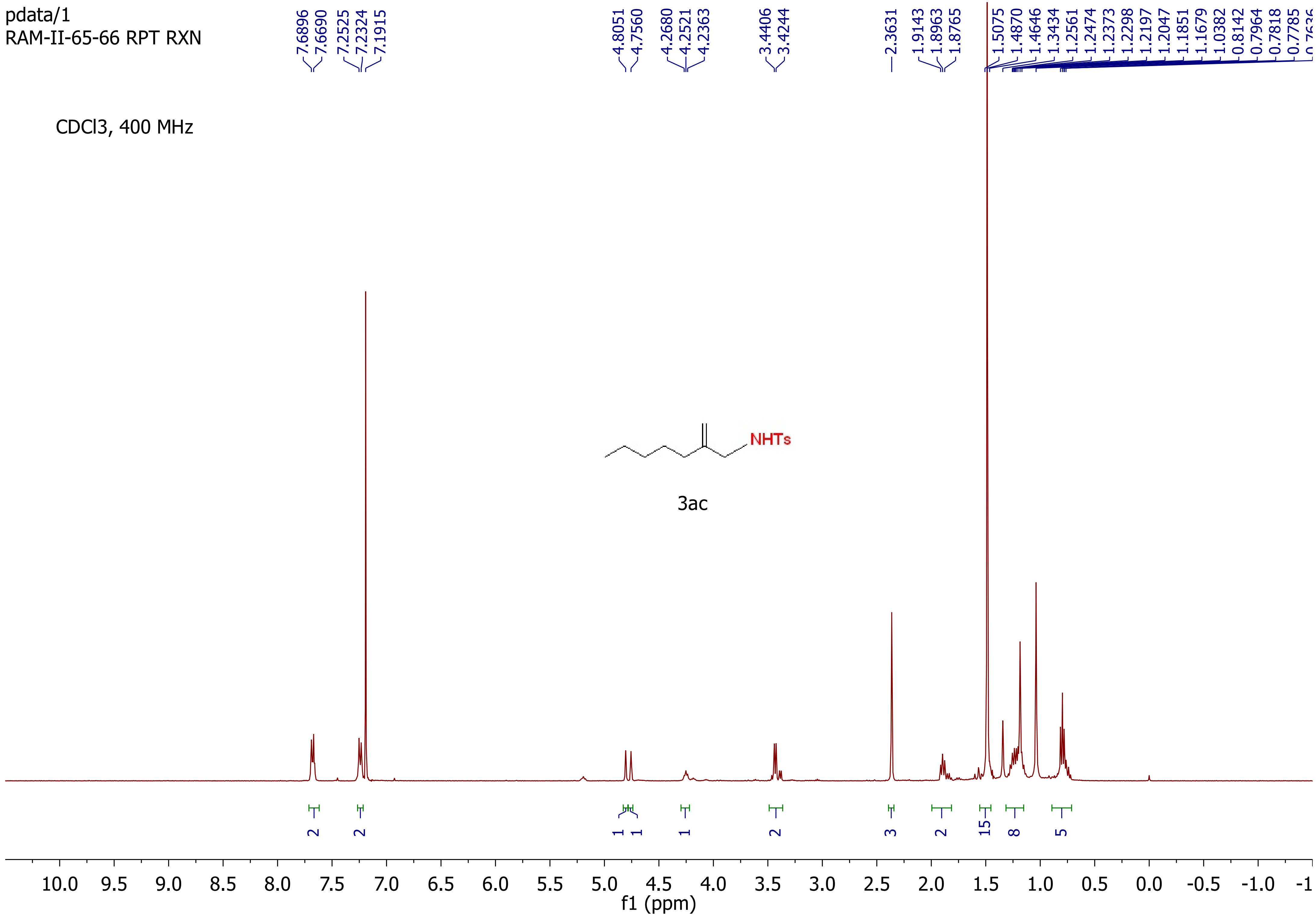

pdata/1
RAM-II-65-66 RPT RXN
CDCl3, 400 MHz
7.6896
7.6690
7.2525
7.2324
7.1915
4.8051
4.7560
4.2680
4.2521
4.2363
3.4406
3.4244
2.3631
1.9143
1.8963
1.8765
1.5075
1.4870
1.4646
1.3434
1.2561
1.2474
1.2373
1.2298
1.2197
1.2047
1.1851
1.1679
1.0382
0.8142
0.7964
0.7818
0.7785
NHTs
3ac
2
2
1
1
1
2
3
2
15
8
5
10.0
9.5
9.0
8.5
8.0
7.5
7.0
6.5
6.0
5.5
5.0
4.5
4.0
3.5
3.0
2.5
2.0
1.5
1.0
0.5
0.0
-0.5
-1.0
f1 (ppm)

pdata/1
RAM-II-65-66 RPT RXN

13C{1H}
CDCl3, 100 MHz

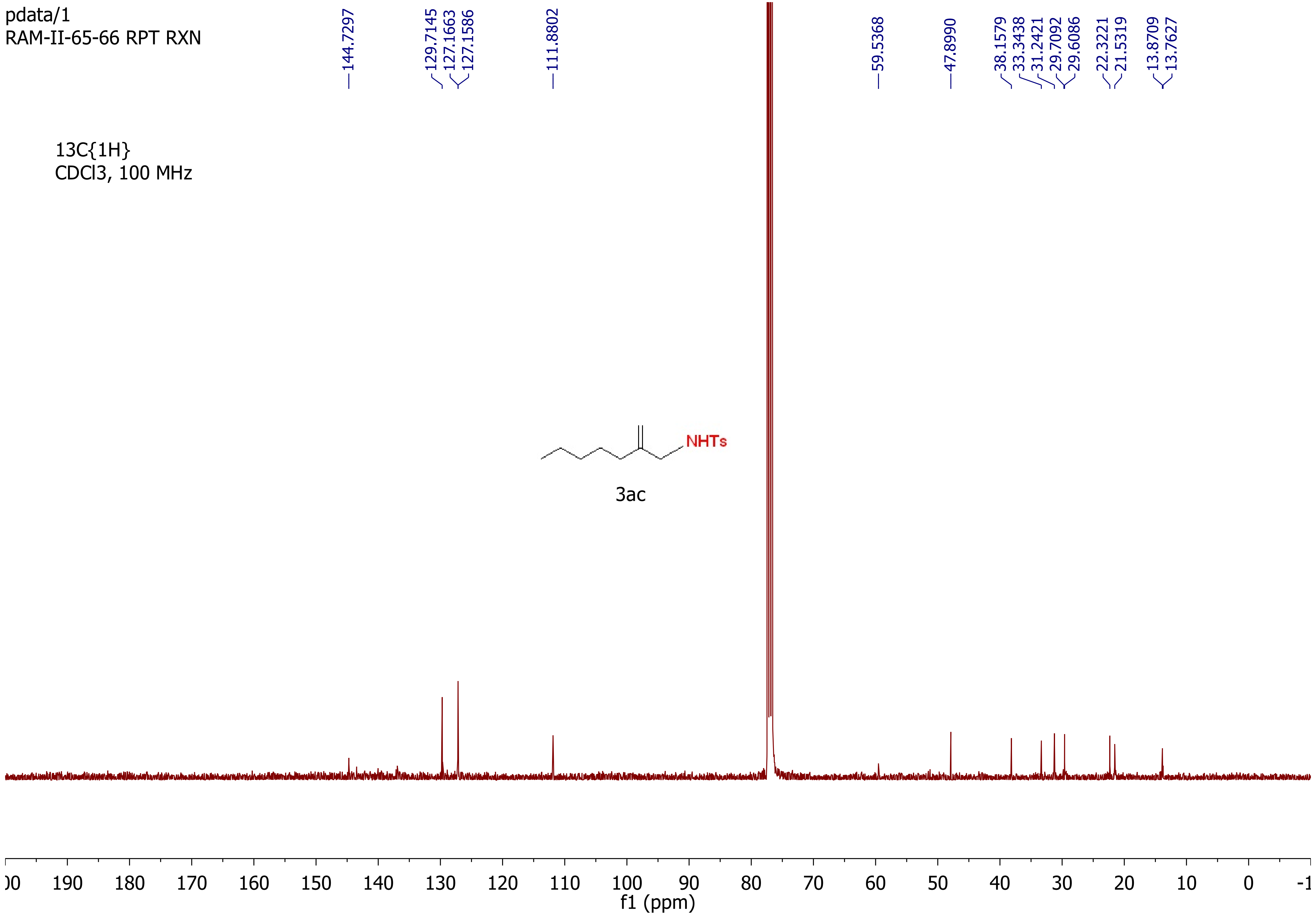

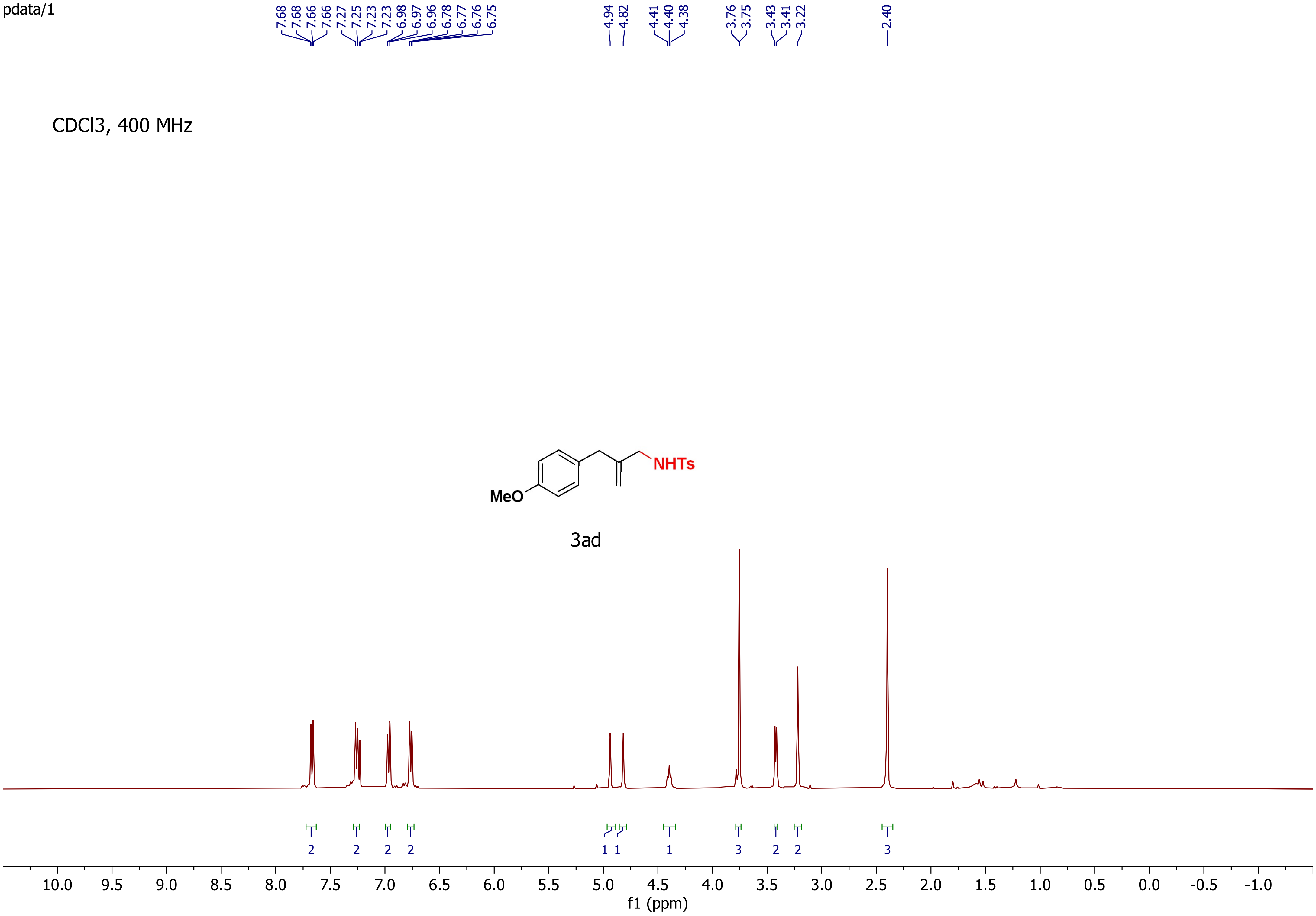
pdata/1
CDCl3, 400 MHz
7.68 7.68 7.66 7.66 7.27 7.25 7.23 7.23 6.98 6.97 6.96 6.78 6.77 6.76 6.75
4.94 4.82 4.41 4.40 4.38 3.76 3.75 3.43 3.41 3.22 2.40
MeO
NHTs
3ad
2 2 2 2 1 1 1 3 2 2 3
10.0 9.5 9.0 8.5 8.0 7.5 7.0 6.5 6.0 5.5 5.0 4.5 4.0 3.5 3.0 2.5 2.0 1.5 1.0 0.5 0.0 -0.5 -1.0
f1 (ppm)

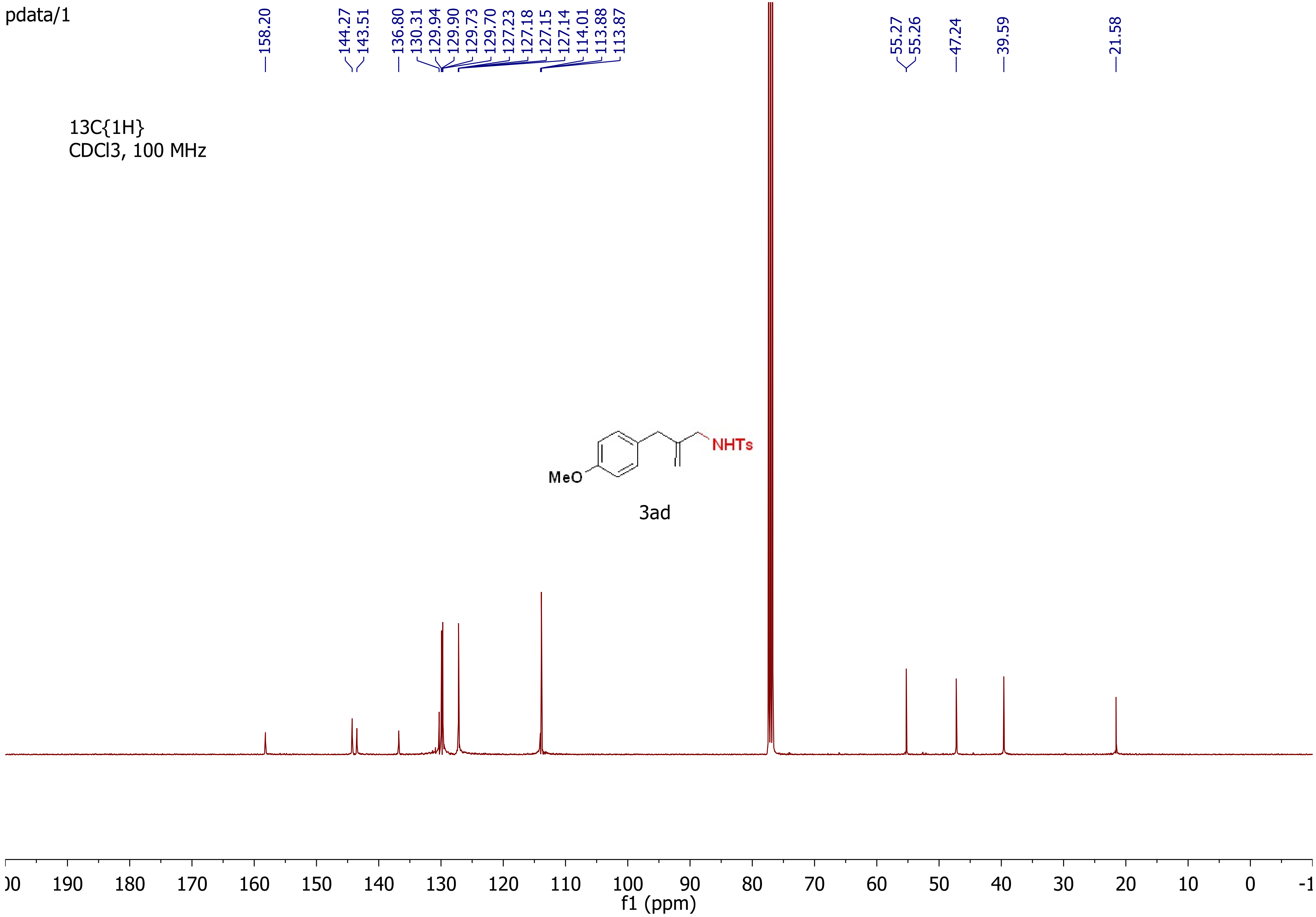
pdata/1
158.20
144.27
143.51
136.80
130.31
129.94
129.90
129.73
129.70
127.23
127.18
127.15
127.14
114.01
113.88
113.87
55.27
55.26
47.24
39.59
21.58
13C{1H}
CDCl3, 100 MHz
MeO
NHTs
3ad
190
180
170
160
150
140
130
120
110
100
90
80
70
60
50
40
30
20
10
0
f1 (ppm)
